# Comparative physiological effects of antipsychotic drugs in children and young people: a network meta-analysis

**DOI:** 10.1016/S2352-4642(24)00098-1

**Published:** 2024-07

**Authors:** Maria Rogdaki, Robert A McCutcheon, Enrico D'Ambrosio, Valentina Mancini, Cameron J Watson, Jack B Fanshawe, Richard Carr, Laurence Telesia, Maria Giulia Martini, Aaron Philip, Barnabas J Gilbert, Gonzalo Salazar-de-Pablo, Marinos Kyriakopoulos, Dan Siskind, Christoph U Correll, Andrea Cipriani, Orestis Efthimiou, Oliver D Howes, Toby Pillinger

**Affiliations:** a Department of Psychosis Studies, Institute of Psychiatry, Psychology and Neuroscience, King's College London, London, UK; b Department of Child and Adolescent Psychiatry, Institute of Psychiatry, Psychology and Neuroscience, King's College London, London, UK; c Social, Genetic and Developmental Psychiatry Centre, Institute of Psychiatry, Psychology and Neuroscience, King's College London, London, UK; d Neuropsychiatry Research and Education Group, Institute of Psychiatry, Psychology and Neuroscience, King's College London, London, UK; e Francis Crick Institute, London, UK; f Department of Psychiatry, University of Oxford, Oxford, UK; g Oxford Precision Psychiatry Lab, NIHR Oxford Health Biomedical Research Centre, Oxford, UK; h Oxford Health NHS Foundation Trust, Warneford Hospital, Oxford, UK; i Department of Translational Biomedicine and Neuroscience, University of Bari Aldo Moro, Bari, Italy; j South London and Maudsley NHS Foundation Trust, London, UK; k Children and Young People Eating Disorder Service, Central and Northwest London NHS Foundation Trust, London, UK; l Great Ormond Street Institute of Child Health, University College London, London, UK; m South West London and St George's Mental Health NHS Trust, London, UK; n Psychiatric Imaging Group, Medical Research Council London Institute of Medical Sciences, Imperial College London, London, UK; o Department of Child and Adolescent Psychiatry, Institute of Psychiatry and Mental Health, Hospital General Universitario Gregorio Marañón School of Medicine, Universidad Complutense, Instituto de Investigación Sanitaria Gregorio Marañón, Centro de Investigación Biomédica en Red de Salud Mental, Madrid, Spain; p 1st Department of Psychiatry, National and Kapodistrian University of Athens, Athens, Greece; q Addiction and Mental Health Service, Metro South Health, Brisbane, QLD, Australia; r Faculty of Medicine, University of Queensland, Brisbane, QLD, Australia; s Zucker Hillside Hospital, Department of Psychiatry, Northwell Health, New York, NY, USA; t Department of Psychiatry and Molecular Medicine, Zucker School of Medicine, Hofstra University, Hempstead, NY, USA; u Department of Child and Adolescent Psychiatry, Charité–Universitätsmedizin Berlin, Berlin, Germany; v Institute of Social and Preventive Medicine and Institute of Primary Health Care, University of Bern, Bern, Switzerland

## Abstract

**Background:**

The degree of physiological responses to individual antipsychotic drugs is unclear in children and adolescents. With network meta-analysis, we aimed to investigate the effects of various antipsychotic medications on physiological variables in children and adolescents with neuropsychiatric and neurodevelopmental conditions.

**Methods:**

For this network meta-analysis, we searched Medline, EMBASE, PsycINFO, Web of Science, and Scopus from database inception until Dec 22, 2023, and included randomised controlled trials comparing antipsychotics with placebo in children or adolescents younger than 18 years with any neuropsychiatric and neurodevelopmental condition. Primary outcomes were mean change from baseline to end of acute treatment in bodyweight, BMI, fasting glucose, total cholesterol, LDL cholesterol, HDL cholesterol, triglycerides, prolactin, heart rate, systolic blood pressure (SBP), and QT interval corrected for heart rate (QTc) for patients receiving either active treatment or placebo. For multigroup trials reporting several doses, we calculated a summary value for each physiological variable for all doses. After transitivity assessment, we fitted frequentist random-effects network meta-analyses for all comparisons in the network. A Kilim plot was used to summarise the results for all treatments and outcomes, providing information regarding the strength of the statistical evidence of treatment effects, using p values. Network heterogeneity was assessed with τ, risk of bias of individual trials was assessed with the Cochrane Collaboration's Tool for Assessing Risk of Bias, and the credibility of findings from each network meta-analysis was assessed with the Confidence in Network Meta-Analysis (CINEMA) app. This study is registered on PROSPERO (CRD42021274393).

**Findings:**

Of 6676 studies screened, 47 randomised controlled trials were included, which included 6500 children (mean age 13·29 years, SD 2·14) who received treatment for a median of 7 weeks (IQR 6–8) with either placebo (n=2134) or one of aripiprazole, asenapine, blonanserin, clozapine, haloperidol, lurasidone, molindone, olanzapine, paliperidone, pimozide, quetiapine, risperidone, or ziprasidone (n=4366). Mean differences for bodyweight change gain compared with placebo ranged from –2·00 kg (95% CI –3·61 to –0·39) with molindone to 5·60 kg (0·27 to 10·94) with haloperidol; BMI –0·70 kg/m^2^ (–1·21 to –0·19) with molindone to 2·03 kg/m^2^ (0·51 to 3·55) with quetiapine; total cholesterol –0·04 mmol/L (–0·39 to 0·31) with blonanserin to 0·35 mmol/L (0·17 to 0·53) with quetiapine; LDL cholesterol –0·12 mmol/L (–0·31 to 0·07) with risperidone or paliperidone to 0·17 mmol/L (–0·06 to 0·40) with olanzapine; HDL cholesterol 0·05 mmol/L (–0·19 to 0·30) with quetiapine to 0·48 mmol/L (0·18 to 0·78) with risperidone or paliperidone; triglycerides –0·03 mmol/L (–0·12 to 0·06) with lurasidone to 0·29 mmol/L (0·14 to 0·44) with olanzapine; fasting glucose from –0·09 mmol/L (–1·45 to 1·28) with blonanserin to 0·74 mmol/L (0·04 to 1·43) with quetiapine; prolactin from –2·83 ng/mL (–8·42 to 2·75) with aripiprazole to 26·40 ng/mL (21·13 to 31·67) with risperidone or paliperidone; heart rate from –0·20 bpm (–8·11 to 7·71) with ziprasidone to 12·42 bpm (3·83 to 21·01) with quetiapine; SBP from –3·40 mm Hg (–6·25 to –0·55) with ziprasidone to 10·04 mm Hg (5·56 to 14·51) with quetiapine; QTc from –0·61 ms (–1·47 to 0·26) with pimozide to 0·30 ms (–0·05 to 0·65) with ziprasidone.

**Interpretation:**

Children and adolescents show varied but clinically significant physiological responses to individual antipsychotic drugs. Treatment guidelines for children and adolescents with a range of neuropsychiatric and neurodevelopmental conditions should be updated to reflect each antipsychotic drug's distinct profile for associated metabolic changes, alterations in prolactin, and haemodynamic alterations.

**Funding:**

UK Academy of Medical Sciences, Brain and Behaviour Research Foundation, UK National Institute of Health Research, Maudsley Charity, the Wellcome Trust, Medical Research Council, National Institute of Health and Care Research Biomedical Centre at King's College London and South London and Maudsley NHS Foundation Trust, the Italian Ministry of University and Research, the Italian National Recovery and Resilience Plan, and Swiss National Science Foundation.

**Research in context**

**Evidence before this study**

We searched PubMed using the search string “antipsychotic AND (children OR adolescent OR youth) AND (weight OR BMI OR cholesterol OR glucose OR prolactin OR blood pressure OR heart rate OR QTc)” from database inception to Dec 22, 2023, limiting the results to meta-analyses but without language restrictions. Selection criteria were network meta-analyses of randomised controlled trials examining antipsychotic treatment in children and adolescents (ie, aged <18 years), in which outcomes were change in physiological variables. Of 95 retrieved meta-analyses, only two examined the association between antipsychotic drug treatment, change in bodyweight, and prolactin. Both were limited to children and adolescents with a diagnosis of schizophrenia and concluded that antipsychotic treatment was associated with bodyweight gain and increased prolactin. No network meta-analysis examined the physiological effects of antipsychotic drugs on children and adolescents across a broader spectrum of neuropsychiatric and neurodevelopmental diagnoses.

**Added value of this study**

To our knowledge, our network meta-analysis is the first to comprehensively map physiological effects of antipsychotic drugs on children and adolescents with neuropsychiatric and neurodevelopmental disorders. The data conclusively show varied but clinically significant alterations of physiological parameters in the paediatric population undergoing antipsychotic treatment, regardless of demographics, baseline physiology, or diagnosis. With the inclusion of randomised controlled trials and a high degree of consistency across network meta-analysis, the findings can be considered robust and generalisable across the paediatric population.

**Implications of all the available evidence**

The probability and severity of alterations of physiological parameters associated with particular antipsychotic drugs can now form a part of evidence-based prescribing decisions for children and adolescents. Transparency when communicating the benefits and risks of antipsychotic treatment will inform the best choice of antipsychotic drug that aligns with the preferences of young people. These findings must be considered in future updates of antipsychotic treatment guidelines for paediatric patients. Future meta-analyses of alterations of physiological parameters during maintenance antipsychotic therapy in young people are indicated to further improve prescribing practice, and by incorporating them into digital tools, they have the potential to facilitate personalised antipsychotic treatment options.

## Introduction

During the past 30 years, there has been an increase in rates of antipsychotic drug prescriptions for children and adolescents younger than 18 years in high-income countries, with an estimated prescription prevalence of 0·5% in 2019.^1^, ^2^ Compared with individuals older than 18 years, antipsychotic treatment in children and adolescents is associated with an increased risk of side-effects,^3^ including large and rapid weight gain and metabolic dysregulation.^4^ Childhood weight gain and metabolic dysregulation can lead to adult obesity, metabolic syndrome, and cardiovascular disease.^5^ Moreover, long-term exposure to prolactin-increasing antipsychotics is associated with reduced bone mineral density^6^ and increased risk of breast cancer.^7^ Thus, there is an association between the physiological side-effects of antipsychotic drugs and long-term physical health conditions. As most children and adolescents continue treatment into adulthood,^8^ reducing the antipsychotic side-effect burden in this population would improve their short-term and long-term physical health outcomes.

Clinical guidelines in the UK recommend that prescription decisions should be made after discussing the contrasting side-effect profiles of different antipsychotics.^9^, ^10^ In practice, such discussions would be facilitated by a ranking system for treatments based on their relative side-effect profiles; however, the evidence to inform such ranking has yet to be synthesised. Whether some paediatric demographic groups might be more vulnerable to the physiological consequences of antipsychotics, as observed in adults,^11^ is also unclear. Moreover, evidence suggests an increased risk of metabolic dysregulation in antipsychotic-naive individuals (>18 years old) with first-episode psychosis compared with the general population worldwide.^12^, ^13^, ^14^ Susceptibility to antipsychotic-induced metabolic side-effects might therefore be higher among children and young people with schizophrenia than among children and young people with other neuropsychiatric and neurodevelopmental disorders. In children and young people with schizophrenia, whether improvement in psychotic symptoms is associated with increased magnitude of antipsychotic-induced metabolic disturbance, as in adults,^11^ is unknown.

By conducting a network meta-analysis of randomised controlled trials of antipsychotic drugs in children and adolescents with a range of neuropsychiatric and neurodevelopmental conditions, we aimed to investigate the effects of different antipsychotic drugs on physiological variables; explore whether age, sex, ethnicity, baseline bodyweight, or a diagnosis of schizophrenia altered vulnerability to antipsychotic-induced physiological alterations; and, in children and adolescents with schizophrenia, examine the relationship between improvement in psychotic symptoms and severity of antipsychotic-induced metabolic disturbance.

## Methods

### Search strategy and selection criteria

This network meta-analysis is reported in line with PRISMA extension statement guidelines (appendix p 3).^15^ We searched Medline, EMBASE, PsycINFO, Web of Science, and Scopus from database inception until Dec 22, 2023, using the search string “(antipsychotic OR [generic/branded antipsychotic names]) AND (random*) AND (children OR adolescents)”. We included randomised controlled trials published in English comparing antipsychotics with placebo in children and adolescents (ie, aged <18 years) with any neuropsychiatric or neurodevelopmental condition (appendix p 6).

Investigators MGM, LT, AP, VM, RC, JBF, ED’A, and CJW worked in pairs to first remove duplicates among the search results and then to screen for titles and abstracts of the remaining results (appendix p 15). Results deemed pertinent to this meta-analysis underwent full-text screening to select reports of randomised controlled trials comparing antipsychotics with placebo in children or adolescents younger than 18 years with neuropsychiatric or neurodevelopmental disorders (appendix p 6). For search results with unpublished data, requests for those data were made by direct inquiry to relevant clinical trial contact persons; if data could not be obtained, then that study was excluded. Discrepancies throughout the selection process were adjudicated by MR and TP and a final decision was made by TP.

### Data analysis

We extracted outcome data for change from baseline (at treatment initiation) to end of acute treatment, in bodyweight, BMI, fasting glucose, total cholesterol, LDL cholesterol, HDL cholesterol, triglycerides, prolactin, heart rate, SBP, and heart rate-corrected QT interval (QTc) for patients receiving active treatment or placebo. Acute treatment was defined as treatment lasting at least 6 weeks. If 6-week treatment data were not available for a selected study, data for treatment periods closest to 6 weeks were used (if a range of treatment durations were used) or studies were excluded (if treatment data were only available for >12 weeks or <3 weeks).^11^ We collected continuous data expressed as mean (SD), mean (SE), or mean (95% CI). Dose limits were not considered in our analysis because little evidence supports a dose-dependent effect of antipsychotic drugs on metabolic or broader endocrine systems.^16^ For multigroup trials reporting several doses of an antipsychotic drug, a summary value for each physiological variable for all doses was calculated with formulas from the Cochrane Handbook.^17^ As paliperidone is the active metabolite of risperidone, data for these drugs were combined.^11^ We also extracted publication-year, baseline-physiological variable level, treatment duration, name of tested antipsychotic drug or drugs, mean age and sex and ethnicity distributions; total symptom change from baseline (mean [SD]) for children and adolescents with schizophrenia.

Transitivity was assessed by examining the distribution of possible effect modifiers across treatment comparisons (age, sex, ethnicity, and bodyweight).^18^, ^19^

### Network meta-analysis

All analyses were done in R version 4.2.2. We fitted frequentist random-effects network meta-analyses, assuming a common random-effects SD (τ), for all comparisons in the network. We chose a random-effects model to reflect possible methodological differences across the trials (eg, in participants, interventions, or definitions of outcomes).^20^ We fitted our models in R using netmeta version 2.7–0.^21^ Physiological change for each variable and treatment comparison was estimated as mean difference (95% CI) except for QTc, for which we calculated standardised mean difference (SMD; 95% CI) due to different calculations used in its derivation.

High heterogeneity in antipsychotic-induced alterations in prolactin in adults was observed in a previous network meta-analysis, which improved with SMD calculation.^22^ Therefore, we conducted the network meta-analysis for changes in prolactin with both mean difference and SMD. For transparent reporting of effect sizes, we avoid dichotomising results as statistically significant or not and instead present effect sizes with 95% CIs.^23^ Placebo is the reference treatment in all forest plots, and league tables display relative cardiometabolic or endocrine changes for all comparisons between antipsychotics. P-scores, as described by Gerta Rücker and Guido Schwarzer,^24^ are used to rank antipsychotics on the basis of amount of metabolic dysregulation. P-scores range from 0 to 1, with a P-score close to 1 indicating increased metabolic disturbance. A Kilim plot was used to summarise the results for all treatments and outcomes, providing information regarding the strength of the statistical evidence of treatment effects, using p values.^25^

We assessed network heterogeneity with τ. Network consistency was evaluated with a global method (ie, a design-by-treatment inconsistency model) and a local method (ie, a back calculation).^26^

Risk of bias of individual trials was assessed with the Cochrane Collaboration's Tool for Assessing Risk of Bias,^26^ with risk of bias classified as high, moderate, or low (appendix p 13). We incorporated results into the Confidence in Network Meta-Analysis (CINEMA)^27^ app to evaluate the credibility of findings from each network meta-analysis. CINEMA grades confidence in the results of each treatment comparison as high, moderate, low, or very low (appendix p 14). Sensitivity analyses including only trials with children and adolescents diagnosed with schizophrenia were done to evaluate generalisability. This study is registered on PROSPERO (CRD42021274393; appendix p 6).

### Pairwise meta-analysis

Pairwise meta-analyses were informed by at least ten trials, accompanied by assessments of heterogeneity, small trial effects, and publication bias through funnel plots and using Egger's test. We drew quantile–quantile plots to assess whether the random effects in the meta-analyses deviated from a normal distribution (appendix p 9).

### Meta-regression analysis

Using the metafor package version 3·8–1,^28^ we conducted random-effects meta-regressions of placebo-controlled trials to examine the relationship between antipsychotic-associated physiological change (the outcome variable) and baseline bodyweight, age, sex, and ethnicity. We drew quantile–quantile plots to assess whether the random effects in the meta-analyses deviated from a normal distribution (appendix p 14). We also drew residual-versus-fitted plots to visually assess the linearity assumptions of our meta-regressions (appendix p 94). We explored the relationship between antipsychotic-induced metabolic change and psychopathology change via a bivariate meta-analysis in participants with schizophrenia (appendix pp 14–15). Analyses were only done if at least ten trials were available. To account for multiple testing, we calculated false discovery rate (FDR).

### Role of the funding source

The funders of the study had no role in study design, data collection, data analysis, data interpretation, or writing of the report.

## Results

**Figure 1:**
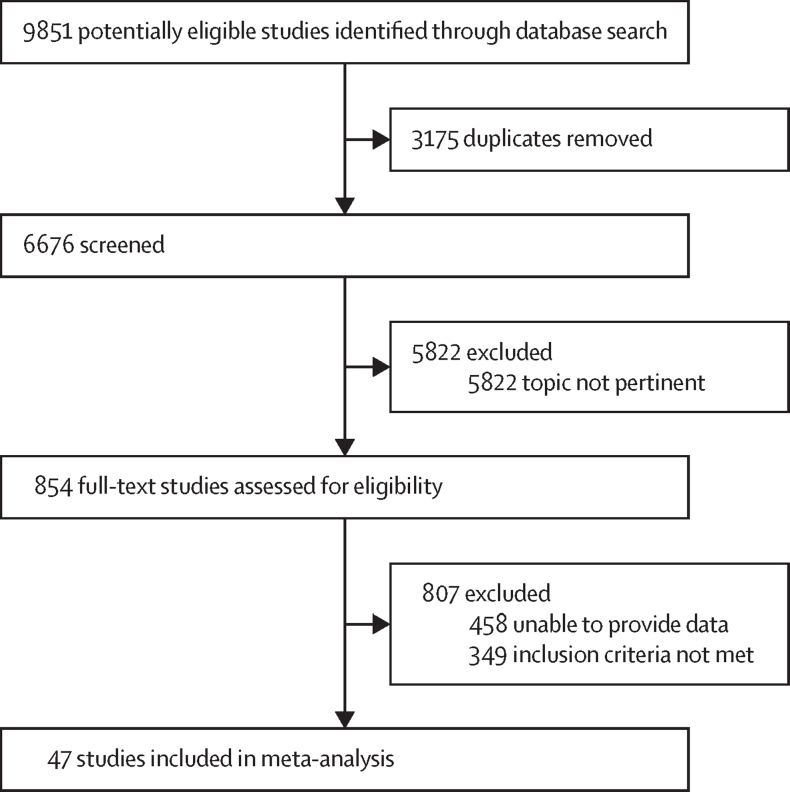
Study selection

**Figure 2:**
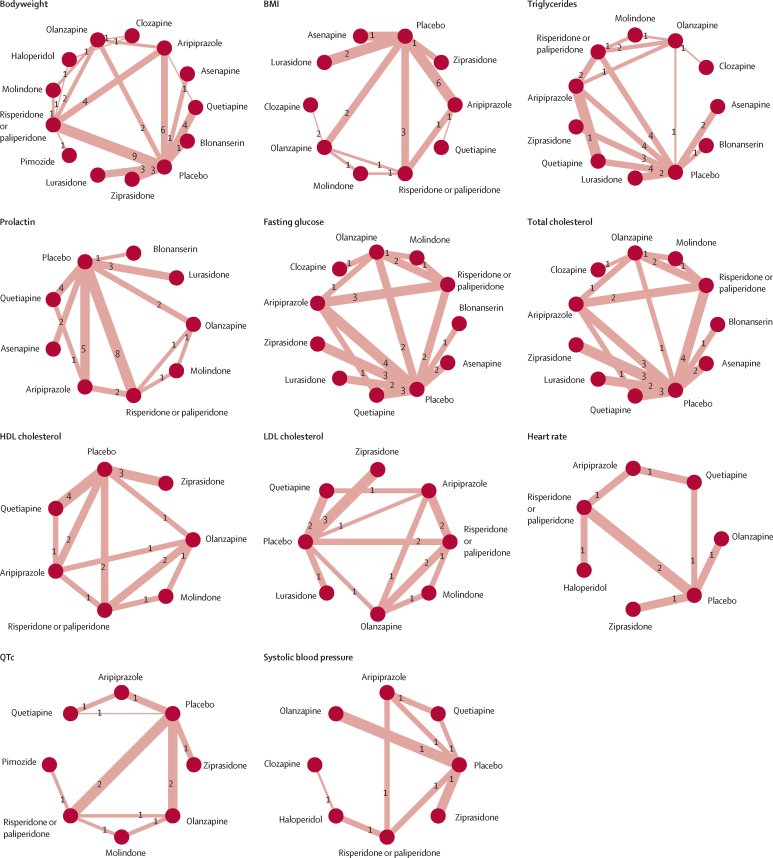
Network graphs of effects of antipsychotic drugs on physiological parameters in children and adolescents Treatments with direct comparisons are linked with a line; the thickness of the line corresponds to the weight of the random effects model comparing the two treatments. Numbers on connecting lines correspond to the number of trials comparing the two treatments. QTc=heart rate-corrected QT interval.

Of 6676 retrieved search results, 47 randomised controlled trials met the inclusion criteria (figure 1). Collectively, these trials examined aripiprazole, asenapine, blonanserin, clozapine, haloperidol, lurasidone, molindone, olanzapine, paliperidone, pimozide, quetiapine, risperidone, and ziprasidone as treatments for schizophrenia, schizophrenia spectrum disorder, bipolar affective disorder, cyclothymia, autism spectrum disorder, conduct disorder, disruptive behavioural disorder, Tourette's syndrome, tic disorders, and anorexia nervosa (figure 2). The overall sample included 6500 children and adolescents, 4366 of whom received antipsychotics and 2134 placebo. Mean age was 13·29 years (SD 2·14). 4075 (62·7%) of 6500 participants were male and 2425 (27·3%) were female. 4472 (68·8%) of 6500 participants were White and 2028 (31·2%) were not White (appendix p 30). Acute treatment duration ranged from 3 weeks to 12 weeks (median 7 weeks, IQR 6–8). Three (6%) of the 47 trials were at high risk of bias (appendix p 28).

Age, sex, bodyweight, and ethnicity of participants were uniformly distributed across treatment comparisons (appendix p 29); as such, we deemed the sample similar enough to synthesise jointly. Data were available for pairwise comparison of the change in weight with placebo and risperidone or paliperidone (appendix pp 29, 30).

We found no clear evidence of small trial effects and publication bias. Although the corresponding contour-enhanced funnel plot had non-significant outcomes (ie, p>0·10), Egger's regression test did not suggest funnel plot asymmetry (p=0·81). Quantile–quantile plots did not show gross deviation from the theoretical normal line (appendix p 31).

**Figure 3:**
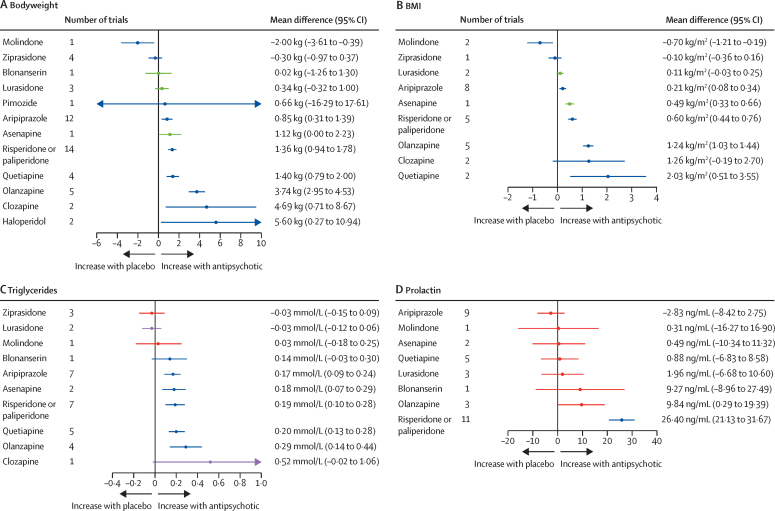
Effect of antipsychotic drugs on bodyweight (A), BMI (B), triglycerides (C), and prolactin (D) relative to placebo Colours indicate CINEMA confidence ratings in the evidence (ie, green is high, blue is moderate, purple is low, and red is very low; appendix pp 33–36). Note that scales differ between panels.

**Figure 4:**
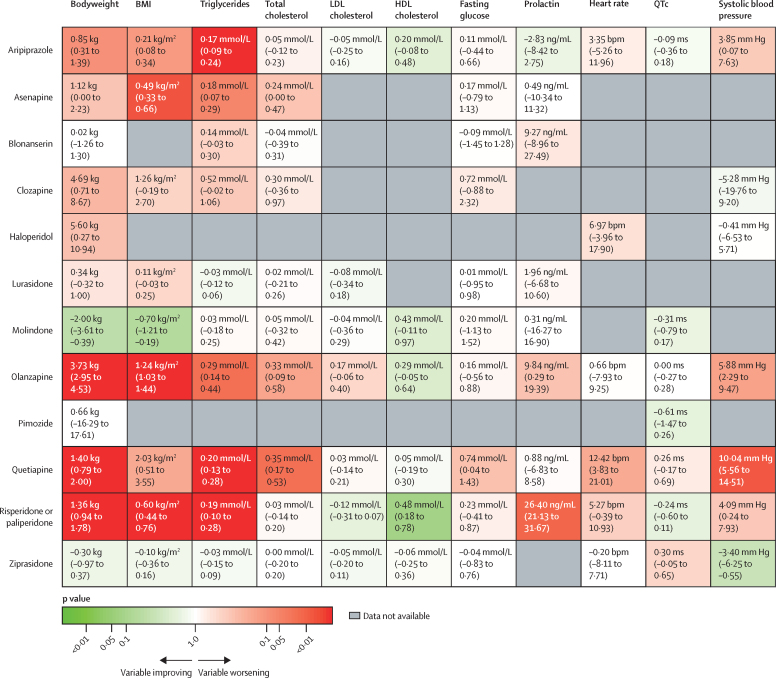
Antipsychotic physiological effects relative to placebo Antipsychotic drugs in this Kilim plot are ranked by mean difference (95% CI) for each variable except QTc, for which data are standardised mean difference (95% CI). Colours correspond to the strength of the statistical evidence supporting the relative effects of a drug versus placebo. For example, deep green indicates strong evidence that a drug is associated with an improvement in that variable compared with placebo (which, for all variables except HDL cholesterol, means a reduction in that variable). Conversely, deep red indicates strong evidence that the drug is associated with a worsening in that variable compared with placebo (which, for all variables except for HDL cholesterol, means an increase in that variable). Colours close to white indicate little evidence on whether the drug performs better or worse than placebo. QTc=heart rate-corrected QT interval.

For change in bodyweight, 41 trials compared 13 different antipsychotics with placebo (3904 participants received active treatment, 1848 placebo; appendix p 33). Bodyweight gain was seen with haloperidol, clozapine, olanzapine, quetiapine, risperidone or paliperidone, asenapine, and aripiprazole. No evidence suggested an effect on bodyweight with pimozide, lurasidone, blonanserin, or ziprasidone. Molindone was associated with a loss in bodyweight (figure 3). Molindone thus ranked highest and haloperidol lowest in terms of effect on bodyweight gain (figure 4; appendix p 44). τ was 0·53 kg, which was considered small in the context of the observed antipsychotic-associated changes. The global test showed network inconsistency (p<0·0001), but the back-calculation method did not (appendix p 47). Certainty of evidence was very low in 15 (19%) of 78 comparisons (appendix p 62).

For BMI, 21 trials compared ten different antipsychotics with placebo (2213 participants received active treatment, 940 placebo; appendix p 34). BMI increased with quetiapine, olanzapine, risperidone or paliperidone, asenapine, and aripiprazole. Some evidence of BMI increase was seen with clozapine and lurasidone, but with increased uncertainty. No clear evidence suggested any effect on BMI with ziprasidone. Molindone was associated with a decrease in BMI (figure 3). Molindone thus ranked highest and quetiapine lowest in terms of effect on BMI increase (figure 4; appendix p 44). τ was 0·00 kg/m^2^. Both global test (p=0·65) and the back-calculation showed no network inconsistency (appendix p 49). On the basis of available data, we deemed that there was no evidence of important heterogeneity or inconsistency in this network meta-analysis. Certainty of evidence was very low in one (2%) of 45 comparisons (appendix p 66).

For triglycerides, 26 trials compared 11 different antipsychotics with placebo (2997 participants received active treatment, 1374 placebo). Triglycerides increased with olanzapine, quetiapine, risperidone or paliperidone, asenapine, and aripiprazole (appendix p 35). We found some evidence of triglyceride increase with clozapine and blonanserin, but with increased uncertainty. No clear evidence suggested any effect on triglyceride with molidone, lurasidone, or ziprasidone (figure 3). Lurasidone thus ranked highest and clozapine lowest in terms of effect on triglycerides (figure 4; appendix p 45). τ was 0·03 mmol/L, which was considered small in the context of the observed antipsychotic-associated changes. The global test showed no inconsistency (p=0·33); however, the back-calculation method showed some network inconsistency (appendix p 51). Certainty of evidence was very low in 22 (40%) of 55 of comparisons (appendix p 69).

For prolactin, 30 trials compared nine different antipsychotics with placebo (3255 participants received active treatment, 1622 placebo). We found very strong evidence of increased prolactin with risperidone or paliperidone. Increased prolactin was also seen with olanzapine. No strong evidence suggested any effect on prolactin with aripiprazole, molindone, asenapine, quetiapine, lurasidone, or blonanserin (figure 3; appendix p 36). Aripiprazole thus ranked highest and risperidone or paliperidone lowest in terms of effect on prolactin (figure 4; appendix p 45). τ was 7·45 ng/mL, which was considered large in the context of the observed antipsychotic-associated changes. The global test showed inconsistency (p<0·0001), although the back-calculation method did not (appendix p 52). On the basis of available data, we deemed that there was evidence of important heterogeneity and inconsistency in this network meta-analysis. Certainty of evidence was very low in 30 (83%) of 36 comparisons (appendix p 72). When SMD was used as the outcome, heterogeneity was lower (appendix p 44). The SMD for increase in prolactin with risperidone or paliperidone relative to placebo was 1·17 (95% CI 0·85–1·49).

**Figure 5:**
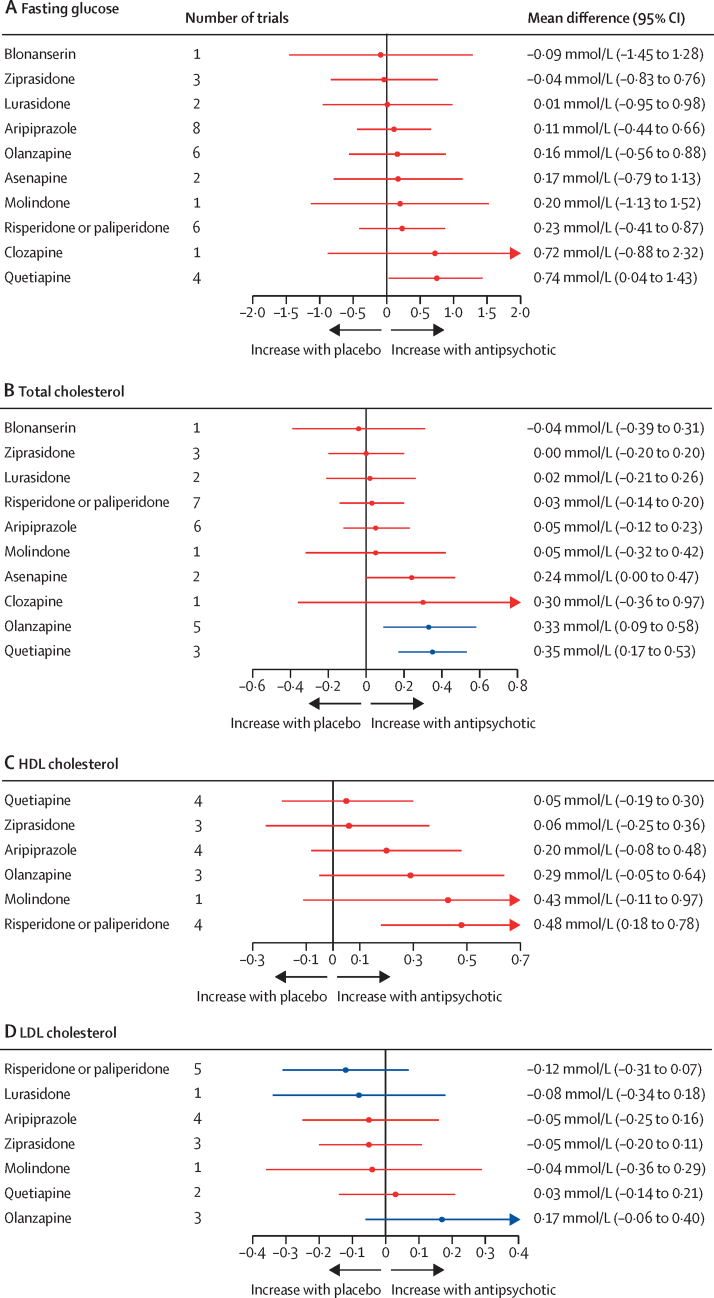
Effect of antipsychotic drugs on fasting glucose (A), total cholesterol (B), HDL cholesterol (C), and LDL cholesterol (D) relative to placebo Colours indicate CINEMA confidence ratings in the evidence (ie, green is high, blue is moderate, purple is low, and red is very low; appendix pp 36–40). Note that scales differ between panels.

For fasting glucose, 25 trials compared 11 different antipsychotics with placebo (3052 participants received active treatment, 1371 placebo). We found strong evidence of increased fasting glucose with quetiapine. Increased fasting glucose was also associated with clozapine, but with increased uncertainty. No strong evidence suggested any change in fasting glucose with blonanserin, ziprasidone, lurasidone, aripiprazole, olanzapine, asenapine, molindone, and risperidone or paliperidone (figure 5; appendix p 36). Ziprasidone thus ranked highest and quetiapine lowest in terms of effect on fasting glucose (figure 4; appendix p 45). τ was 0·69 mmol/L, which was considered large in the context of observed alterations. The global test showed inconsistency (p<0·0001), but the back-calculation method did not (appendix p 54). Certainty of evidence was very low in 55 (100%) of 55 of comparisons (appendix p 74).

For total cholesterol, 25 trials compared 11 different antipsychotics with placebo (3040 participants received active treatment, 1368 placebo; appendix p 38). Increased total cholesterol was associated with quetiapine, olanzapine, and asenapine. Some evidence of total cholesterol change was seen with clozapine, but with increased uncertainty. No strong evidence suggested any change in total cholesterol with blonanserin, ziprasidone, lurasidone, risperidone or paliperidone, aripiprazole, and molindone (figure 5). Blonanserin thus ranked highest and quetiapine lowest in terms of effect on total cholesterol (figure 4; appendix p 45). τ was 0·15 mmol/L, which was considered large in the context of the observed antipsychotic-associated changes. The global test (p<0·0001) and the back-calculation method showed inconsistency (appendix p 55). Certainty of evidence was very low in 51 (93%) of 55 comparisons (appendix p 78).

For HDL cholesterol, 15 trials compared seven different antipsychotics with placebo (1528 participants received active treatment, 686 placebo; appendix p 39). Increased HDL cholesterol was associated with risperidone or paliperidone (figure 5). τ was 0·27 mmol/L, which was considered large in the context of the observed antipsychotic-associated changes. The global test (p<0·0001) and the back-calculation method showed inconsistency (appendix p 57). Certainty of evidence was very low in 21 (100%) of 21 comparisons (appendix p 81).

For LDL cholesterol, 14 trials compared eight different antipsychotics with placebo (1589 participants received active treatment; 672 placebo; appendix p 39). No strong evidence suggested any effect on LDL cholesterol by antipsychotics (figure 5). τ was 0·12 mmol/L, which was considered large in the context of the observed antipsychotic-associated changes. The global test (p=0·0016) and the back-calculation method showed inconsistency (appendix p 58). Certainty of evidence was very low in 23 (82%) of 28 comparisons (appendix p 82).

**Figure 6:**
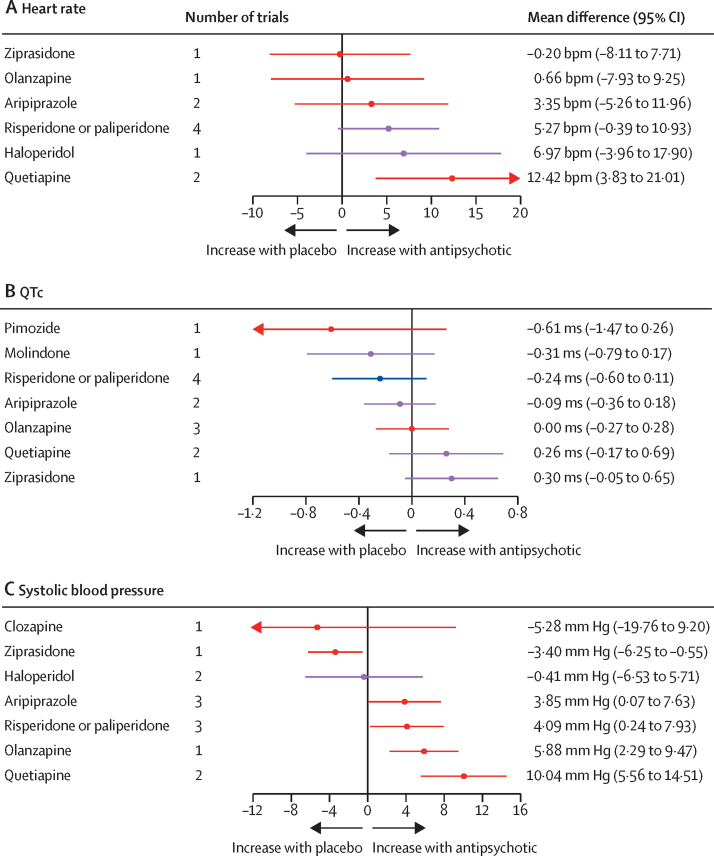
Effect of antipsychotic drugs on heart rate (A), QTc interval (B), and systolic blood pressure (C)relative to placebo Colours indicate CINEMA confidence ratings in the evidence (ie, green is high, blue is moderate, purple is low, and red is very low; appendix pp 40–42). Note that scales differ between panels. QTc=heart rate-corrected QT interval.

For heart rate, eight trials compared seven different antipsychotics with placebo (537 participants received active treatment, 217 placebo; appendix p 40). Increased heart rate was associated with quetiapine. Some evidence of heart rate changes was seen with haloperidol, risperidone or paliperidone, and aripiprazole, but with greater uncertainty. No strong evidence suggested any change in heart rate with olanzapine or ziprasidone (figure 6). Ziprasidone thus ranked highest and quetiapine lowest in terms of effect on heart rate (figure 4; appendix p 46). τ was 3·59 bpm, which was considered moderate in the context of the observed antipsychotic-associated changes. The global test (p=0·071) and the back-calculation method showed no inconsistency (appendix p 59). Certainty of evidence was very low in 17 (81%) of 21 comparisons (appendix p 86).

For QTc, ten trials compared eight different antipsychotics with placebo (788 participants received active treatment, 270 placebo; appendix p 41). Some evidence of increased QTc was seen with ziprasidone and quetiapine, but with a degree of uncertainty. Little evidence was found of QTc increases with olanzapine, aripiprazole, risperidone or paliperidone, molindone, and pimozide (figure 6). Pimozide thus ranked highest and ziprasidone lowest in terms of effect on QTc (figure 4; appendix p 46). τ was 0·071, which was considered small to moderate in the context of observed changes. The global test (p=0·37) and the back-calculation method showed no inconsistency (appendix p 59). Certainty of evidence was very low in 14 (50%) of 28 comparisons (appendix p 84).

For SBP, nine trials compared eight different antipsychotics with placebo (535 participants received active treatment, 189 placebo; appendix p 42). Increases in SBP were seen with quetiapine, olanzapine, risperidone or paliperidone, and aripiprazole. We found evidence of reduced blood pressure with ziprasidone. Little evidence suggested any effect on blood pressure change with haloperidol and clozapine (figure 6). Ziprasidone thus ranked highest and quetiapine lowest in terms of effect on SBP (figure 4; appendix p 46). τ was 0·00 ms. The global test (p=0·48) and the back-calculation method showed no inconsistency (appendix p 60). Certainty of evidence was very low in 17 (61%) of 28 comparisons (appendix p 87).

The results of sensitivity analyses examining antipsychotic-induced changes in physiological variables in children and adolescents with schizophrenia alone were similar to those for the wider population of children with neuropsychiatric and neurodevelopmental conditions (appendix pp 90–92).

With the exception of fasting glucose, quantile–quantile plots for the meta-regression models showed no gross deviation from the theoretical normal line (appendix pp 93, 94). The residual-versus-fitted plots showed no strong evidence of violation of the linearity assumption (appendix p 94). We found some evidence that large antipsychotic-induced increases in prolactin were more prevalent among White participants than non-White participants; however, this result did not hold after correction for multiple comparisons (study number 14, estimate 1·11 ng/mL per 1% increase in proportion of White participants, 95% CI 0·11 to 2·10; p=0·029; p_FDR_=0·20). We found no strong evidence of an association between possible effect modifiers and any other physiological variables (appendix p 95). Insufficient data were available to examine the association between changes in metabolic variables and psychopathology.

## Discussion

To our knowledge, this network meta-analysis is the first to comprehensively examine the physiological effects of antipsychotic drugs on children and adolescents with a range of neuropsychiatric and neurodevelopmental disorders. We found substantial variation in the effect of antipsychotics on bodyweight, BMI, triglycerides, total cholesterol, prolactin, heart rate, and SBP. Antipsychotic-induced effects were large and considered clinically relevant for bodyweight, BMI, fasting glucose, triglycerides, heart rate, and SBP.

In agreement with previous studies,^29^, ^30^, ^31^ weight gain was more common with olanzapine, quetiapine, clozapine, and risperidone or paliperidone than with other treatments. The finding is also consistent with the results of a meta-analysis and meta-regression of weight and BMI increase in children and adolescents exposed to antipsychotic drugs in non-interventional settings.^32^ Furthermore, similar to studies in adults, we estimated that fasting glucose and lipid alterations were greater with quetiapine and olanzapine than with other antipsychotics.^11^ One previous meta-analysis of observational data of acute antipsychotic treatment of children and adolescents with schizophrenia spectrum disorders suggested that quetiapine increased triglycerides.^31^ By using data from randomised controlled trials and showing increased triglyceride concentration by several antipsychotics, we have extended the field's understanding. Quetiapine was associated with the largest increase in heart rate and SBP. Risperidone or paliperidone ranked highest in terms of associated change in prolactin, which is consistent with the conclusion of a previous, smaller meta-analysis in paediatric patients with schizophrenia and schizophrenia spectrum disorders.^33^ Molindone ranked highest in terms of associated bodyweight loss, which is consistent with previous data from adults with schizophrenia.^34^

Aripiprazole, blonanserin, lurasidone, and ziprasidone showed relatively benign physiological side-effect profiles. The most prominent antipsychotic-induced increases in prolactin were seen among White participants. However, this association might have been a chance finding as multiple analyses were done, so the finding warrants further study. By contrast to a similar network meta-analysis in adults with schizophrenia,^11^ we found no evidence that the severity of antipsychotic-induced physiological change was associated with bodyweight, sex, or ethnicity. Furthermore, similar results for children with neuropsychiatric and neurodevelopmental conditions compared with children and adolescents with a diagnosis of schizophrenia suggest uniform susceptibility to antipsychotic physiological effects across the paediatric population.

We included all randomised controlled trials of children and adolescents with a range of neuropsychiatric and neurodevelopmental disorders to improve the generalisability of our findings. No inconsistency was found in our network meta-analyses of changes in weight, BMI, fasting glucose, prolactin, SBP, heart rate, or QTc, supporting the robustness of our results. However, global inconsistency was seen in the network meta-analyses of changes in weight, fasting glucose, and prolactin (although minimal evidence suggested local inconsistency). Local and global inconsistency was also seen in the network meta-analyses of changes in total cholesterol, HDL cholesterol, and LDL cholesterol. Potential reasons include imbalances in the distribution of some effect modifiers across comparisons, small trial effects, and publication bias.

Haloperidol was associated with the greatest degree of weight gain, which contrasts with conclusions from similar network meta-analyses in adults with schizophrenia.^11^ However, our result could be a statistical artefact caused by a single small trial; future studies are warranted.

An important limitation of our network meta-analyses is that—except for analyses of changes in bodyweight, BMI, triglycerides, and QTc—confidence in the evidence was generally very low for more than 50% of treatment comparisons, largely resulting from large imprecision in estimated effects. A similar issue was seen in the network meta-analysis of acute comparative metabolic effects of antipsychotics in adults with schizophrenia, with confidence of outcomes for up to 100% of treatment comparisons regarded as either low or very low.^11^ Although we did not identify demographic or physiological predictors of antipsychotic-induced physiological alterations, such analyses should ideally be done as network meta-analyses of individual participant data—an important focus for future research.^35^ Furthermore, insufficient trial numbers meant we were unable to explore the relationship between physiological changes and improvements in psychopathology in children and adolescents with schizophrenia, so this topic should also be examined in future studies. Future meta-analyses could also focus on specific clinical outcomes such as rates of emergent diabetes rather than simply mean change in metabolic variables. As metabolic outcomes were invariably not available in trials older than 30 years, our data were mainly generated by trials of antipsychotics licensed in the past 35 years. To improve prescribing practice, further work is needed to define the metabolic profile of older drugs in children and adolescents with neuropsychiatric and neurodevelopmental disorders. Trials also did not typically provide information on lifestyle factors (eg, diet and exercise), physical comorbidities, puberty stages, or co-prescribed medication, all of which could have influenced physiological variables. Nevertheless, because of randomisation, we expect no systematic imbalances in such confounding factors between groups in our network meta-analyses. However, we could not check their distributions across treatment comparisons in the network, in which imbalances might jeopardise network transitivity. Finally, we focused on short-term trials and effects. However, as physiological alterations accumulate with time and antipsychotic drugs are often prescribed for long-term treatment,^36^ a network meta-analysis of physiological side-effects of antipsychotic maintenance therapy in children and adolescents is indicated.

Increased BMI and dyslipidaemia in childhood increase the risk of cardiovascular disease in adulthood.^37^ Furthermore, long-term exposure to prolactin-increasing antipsychotics is associated with reduced bone mineral density^6^ and increased risk of breast cancer.^7^ We found that acute treatment for a median duration of 7 weeks (IQR 6–8) with antipsychotics such as quetiapine and olanzapine increased BMI by 1–2 kg/m^2^; furthermore, increases in prolactin were more prominent with risperidone or paliperidone treatment than with other antipsychotic drugs. Risperidone, quetiapine, and olanzapine are three of the four most prescribed antipsychotic drugs for children and adolescents worldwide^2^ and, considering that most children and adolescents will continue their antipsychotic treatment into adulthood,^8^ our findings have clear implications for clinical practice. Aripiprazole, blonanserin, lurasidone, molindone, and ziprasidone can be broadly considered safer treatment options.

Altogether, the results of this network meta-analysis should inform future prescription decisions. However, when choosing an antipsychotic, side-effects such as sedation and extrapyramidal effects should also be considered, as should treatment efficacy.^38^ Our results could also be incorporated alongside other data sources into digital tools that collate side-effect and effectiveness data to facilitate personalised antipsychotic treatment options.^39^ This approach can be used across the paediatric population, regardless of demographics, baseline physiology, or diagnosis.

In conclusion, substantial variation was seen in the effects of antipsychotics on various physiological variables when used for children and adolescents with a range of neuropsychiatric and neurodevelopmental disorders. For metabolic changes, olanzapine, quetiapine, risperidone, paliperidone, and clozapine had the worst side-effect profiles, whereas aripiprazole, blonanserin, ziprasidone, lurasidone, and molindone had relatively benign profiles. For prolactin, risperidone or paliperidone had the worst profile, and aripiprazole had the best profile. For haemodynamic and QTc effects, quetiapine had a broadly poor profile and no antipsychotic had a consistently benign profile across variables. These findings should inform future updates to clinical treatment guidelines. However, we caution that choice of antipsychotic should be made on an individual basis, considering clinical circumstances, benefits and risks of individual treatment options, and the preferences of the child or adolescent, and their carer and clinicians.

### Contributors

### Data sharing

The summary data used in analyses (change in a given physiological parameter expressed as mean [SD]) can be provided from the corresponding author on request and without restriction. These data will be available from date of publication.

## Declaration of interests

RAM has participated in speaker meetings for Otsuka, Karuna, and Janssen and in advisory boards for Viatris, Boehringer Ingelheim, and Karuna. ED’A has received lecture fees from Lundbeck. GS-d-P has participated in advisory and speaker meetings for Jansen and Menarini. OE has received honoraria and consulting fees from Biogen, paid to his institution. AC has received research, educational, and consultancy fees from the Italian Network for Paediatric Trials, the Cariplo Foundation, Lundbeck, and Angelini Pharma and is the chief investigator of a randomised controlled trial of seltorexant for adolescents with depression that is sponsored by Janssen. CUC has received consultancy fees as an advisor from Alkermes, Angelini, Boehringer Ingelheim, Cardio Diagnostics, Cerevel, CNX Therapeutics, Compass Pathways, Gedeon Richter, Holmusk, IntraCellular Therapies, Janssen, Johnson & Johnson, Karuna, LB Pharma, Lundbeck, MedAvante–ProPhase, Merck, Mindpax, Mitsubishi Tanabe Pharma, Neurelis, Newron, Noven, Novo Nordisk, Otsuka, Pharmabrain, PPD Biotech, Recordati, Rovi, Seqirus, SK Life Science, Sunovion, Supernus, Takeda, Teva, and Viatris; has received speaker fees from AbbVie, Alkermes, Angelini, Aristo, Boehringer Ingelheim, Cerevel, Darnitsa, Denovo, Gedeon Richter, Hikma, Janssen, Johnson & Johnson, Karuna, Lundbeck, Mylan, Otsuka, Recordati, Rovi, Seqirus, Sunovion, Sun Pharma, Takeda, Teva, and Viatris; has received honoraria from Allergan, Biogen, Relmada, Reviva, and Supernus; has provided expert testimony for Janssen and Otsuka; was on a data safety monitoring board for Compass Pathways, Denovo, Lundbeck, Relmada, Reviva, Rovi, Sage, Supernus, Tolmar, and Teva; has received grant support from Janssen and Takeda; has received royalties from UpToDate; and is a stock option holder of Cardio Diagnostics, Mindpax, LB Pharma, PsiloSterics, and Quantic. ODH has received investigator-initiated research funding from and participated in advisory and speaker meetings for Angellini, Autifony, Biogen, Boehringer Ingelheim, Eli Lilly, Heptares, Global Medical Education, Invicro, Janssen, Lundbeck, Neurocrine, Otsuka, Sunovion, Rand, Recordati, Roche, ROVI Biotech, Viatris, and Mylan. TP has participated in educational speaker meetings for Lundbeck, Otsuka, Sunovion, Janssen, Schwabe Pharma, ROVI Biotech, and Recordati and receives book royalties from Wiley Blackwell. TP and RAM co-direct Pharmatik, which designs digital resources to support treatment of mental illness. All other authors declare no competing interests.
