# Supplementary material for: Comparative physiological effects of antipsychotic drugs in children and young people: a network meta-analysis
Source: Lancet Child Adolesc Health. 2024 Jul;8(7):510–21. doi: 10.1016/S2352-4642(24)00098-1 (PMC11790527; doi:10.1016/S2352-4642(24)00098-1)
Supplement: Supplementary appendix [file mmc1.pdf]

THE LANCET
Child & Adolescent Health

Supplementary appendix

This appendix formed part of the original submission and has been peer reviewed. We post it as supplied by the authors.

Supplement to: Rogdaki M, McCutcheon RA, D'Ambrosio E, et al. Comparative physiological effects of antipsychotic drugs in children and young people: a network meta-analysis. *Lancet Child Adolesc Health* 2024; **8:** 510–21.

SUPPLEMENTARY INFORMATION

Comparative physiological effects of antipsychotics in children and young people: a network meta-analysis

Rogdaki M, McCutcheon R, D'Ambrosio E, Mancini V, Watson C, Fanshawe, Carr R, Telesia L, Martini GM, Philip A, Gilbert B J, Salazar-de-Pablo G, Kyriakopoulos M, Siskind D, Correll CU, Cipriani A, Efthimiou O, Howes O, Pillinger T

CONTENTS

Page 3-5	**eTable 1.** PRISMA NMA checklist
Pages 6-13	**eAppendix 1.** PROSPERO registration and protocol
Pages 13-14	**eAppendix 2.** Methods to assess confidence in NMA using CINeMA
Page 14	**eAppendix 3.** Metaregression
Pages 15-16	**eAppendix 4.** Assessing the relationship between alterations in physiological parameters and psychopathology
Pages 16-27	**eTable 2.** Studies included in the meta-analysis
Pages 28-29	**eTable 3.** Cochrane risk of bias assessment
Page 29	**eTable 4.** Baseline parameters across treatment-comparisons
Pages 29-30	**eAppendix 5.** Pairwise meta-analyses
Page 30	**eFigure 1.** Pairwise meta-analysis forest plot: placebo versus risperidone/paliperidone.
Page 30	**eFigure 2.** Pairwise meta-analysis contour-enhanced funnel plot: placebo versus risperidone/paliperidone.
Page 31	**eFigure 3.** QQ plots: Change in weight, risperidone/paliperidone vs placebo
Page 31	**eFigure 4.** QQ plots: Change in weight, aripiprazole vs placebo
Page 32	**eFigure 5:** QQ plots: Change in BMI, aripiprazole vs placebo
Page 32	**eFigure 6:** QQ plots: Change in prolactin, risperidone/paliperidone vs placebo
Pages 33-42	**eTables 5-15** League tables comparing alterations of physiological parameters associated with antipsychotic drug treatment.
Page 43	**eAppendix 6.** Full results for HDL- cholesterol and LDL-cholesterol
Page 44	**eFigure 7**. Prolactin elevation: SMD results
Pages 44-46	**eTables 16-25**. P-score ranking for alterations of physiological parameters associated with antipsychotic drug treatment
Pages 47-61	**eTables 26-36.** Identifying 'hot spots' of inconsistency for the 8 network meta-analyses
Pages 62-89	**eTables 37-46.** CINeMA confidence ratings for the drug comparisons making up the 8 network meta-analyses.
Pages 90-91	**eAppendix 7.** Sensitivity analysis: antipsychotic-induced physiological changes in children and young people with schizophrenia
Page 92	**eFigure 8.** Estimates of mean differences of antipsychotic drugs compared with placebo obtained from network meta-analyses.
Page 93	**eAppendix 8.** QQ plots for pairwise meta-regression models
Page 94	**eAppendix 9.** Residual versus fitted plots for pairwise meta-regression models
Pages 95	**eAppendix 10.** Metaregression on the effect of sex, ethnicity, and baseline body weight on antipsychotic-induced physiological alterations
Pages 96-100	**Supplementary References**

eTable 1. PRISMA NMA Checklist

Section/Topic	Item #	Checklist Item	Reported on Page #
**TITLE**			
Title	1	Identify the report as a systematic review *incorporating a network meta-analysis (or related form of meta-analysis).*	1
**ABSTRACT**			
Structured summary	2	Provide a structured summary including, as applicable:<br>**Background:** main objectives<br>**Methods:** data sources; study eligibility criteria, participants, and interventions; study appraisal; and *synthesis methods, such as network meta-analysis.*<br>**Results:** number of studies and participants identified; summary estimates with corresponding confidence/credible intervals; *treatment rankings may also be discussed. Authors may choose to summarize pairwise comparisons against a chosen treatment included in their analyses for brevity.*<br>**Discussion/Conclusions:** limitations; conclusions and implications of findings.<br>**Other:** primary source of funding; systematic review registration number with registry name.	1
**INTRODUCTION**			
Rationale	3	Describe the rationale for the review in the context of what is already known*, including mention of why a network meta-analysis has been conducted.*	2-3
Objectives	4	Provide an explicit statement of questions being addressed, with reference to participants, interventions, comparisons, outcomes, and study design (PICOS).	3
**METHODS**			
Protocol and registration	5	Indicate whether a review protocol exists and if and where it can be accessed (e.g., Web address); and, if available, provide registration information, including registration number.	3
Eligibility criteria	6	Specify study characteristics (e.g., PICOS, length of follow-up) and report characteristics (e.g., years considered, language, publication status) used as criteria for eligibility, giving rationale. *Clearly describe eligible treatments included in the treatment network, and note whether any have been clustered or merged into the same node (with justification).*	3
Information sources	7	Describe all information sources (e.g., databases with dates of coverage, contact with study authors to identify additional studies) in the search and date last searched.	3
Search	8	Present full electronic search strategy for at least one database, including any limits used, such that it could be repeated.	**Sup info**
Study selection	9	State the process for selecting studies (i.e., screening, eligibility, included in systematic review, and, if applicable, included in the meta-analysis).	3
Data collection process	10	Describe method of data extraction from reports (e.g., piloted forms, independently, in duplicate) and any processes for obtaining and confirming data from investigators.	3

Data items	11	List and define all variables for which data were sought (e.g., PICOS, funding sources) and any assumptions and simplifications made.	4
**Geometry of the network**	**S1**	Describe methods used to explore the geometry of the treatment network under study and potential biases related to it. This should include how the evidence base has been graphically summarized for presentation, and what characteristics were compiled and used to describe the evidence base to readers.	
Risk of bias within individual studies	12	Describe methods used for assessing risk of bias of individual studies (including specification of whether this was done at the study or outcome level), and how this information is to be used in any data synthesis.	4
Summary measures	13	State the principal summary measures (e.g., risk ratio, difference in means). *Also describe the use of additional summary measures assessed, such as treatment rankings and surface under the cumulative ranking curve (SUCRA) values, as well as modified approaches used to present summary findings from meta-analyses.*	4
Planned methods of analysis	14	Describe the methods of handling data and combining results of studies for each network meta-analysis. This should include, but not be limited to:<br>• *Handling of multi-arm trials;*<br>• *Selection of variance structure;*<br>• *Selection of prior distributions in Bayesian analyses; and*<br>• *Assessment of model fit.*	4
**Assessment of Inconsistency**	**S2**	Describe the statistical methods used to evaluate the agreement of direct and indirect evidence in the treatment network(s) studied. Describe efforts taken to address its presence when found.	4
Risk of bias across studies	15	Specify any assessment of risk of bias that may affect the cumulative evidence (e.g., publication bias, selective reporting within studies).	4
Additional analyses	16	Describe methods of additional analyses if done, indicating which were pre-specified. This may include, but not be limited to, the following:<br>• Sensitivity or subgroup analyses;<br>• Meta-regression analyses;<br>• *Alternative formulations of the treatment network; and*<br>• *Use of alternative prior distributions for Bayesian analyses (if applicable).*	4
**RESULTS**			
Study selection	17	Give numbers of studies screened, assessed for eligibility, and included in the review, with reasons for exclusions at each stage, ideally with a flow diagram.	5<br>eTable 2
**Presentation of network structure**	**S3**	Provide a network graph of the included studies to enable visualization of the geometry of the treatment network.	Fig 2
**Summary of network geometry**	**S4**	Provide a brief overview of characteristics of the treatment network. This may include commentary on the abundance of trials and randomized patients for the different interventions and pairwise comparisons in the network, gaps of evidence in the treatment network, and potential biases reflected by the network structure.	
Study characteristics	18	For each study, present characteristics for which data were extracted (e.g., study size, PICOS, follow-up period) and provide the citations.	eTable 2
Risk of bias within studies	19	Present data on risk of bias of each study and, if available, any outcome level assessment.	eTable 3

Results of individual studies	20	For all outcomes considered (benefits or harms), present, for each study: 1) simple summary data for each intervention group, and 2) effect estimates and confidence intervals. *Modified approaches may be needed to deal with information from larger networks.*	eTable 4
Synthesis of results	21	Present results of each meta-analysis done, including confidence/credible intervals. *In larger networks, authors may focus on comparisons versus a particular comparator (e.g. placebo or standard care), with full findings presented in an appendix. League tables and forest plots may be considered to summarize pairwise comparisons.* If additional summary measures were explored (such as treatment rankings), these should also be presented.	Fig 3 eTables 5-15
**Exploration for inconsistency**	**S5**	Describe results from investigations of inconsistency. This may include such information as measures of model fit to compare consistency and inconsistency models, *P* values from statistical tests, or summary of inconsistency estimates from different parts of the treatment network.	7-10
Risk of bias across studies	22	Present results of any assessment of risk of bias across studies for the evidence base being studied.	Figure 3 eTable 3 eTables 37-46
Results of additional analyses	23	Give results of additional analyses, if done (e.g., sensitivity or subgroup analyses, meta-regression analyses*, alternative network geometries studied, alternative choice of prior distributions for Bayesian analyses,* and so forth).	10
**DISCUSSION**			
Summary of evidence	24	Summarize the main findings, including the strength of evidence for each main outcome; consider their relevance to key groups (e.g., healthcare providers, users, and policy-makers).	10
Limitations	25	Discuss limitations at study and outcome level (e.g., risk of bias), and at review level (e.g., incomplete retrieval of identified research, reporting bias). *Comment on the validity of the assumptions, such as transitivity and consistency. Comment on any concerns regarding network geometry (e.g., avoidance of certain comparisons).*	11
Conclusions	26	Provide a general interpretation of the results in the context of other evidence, and implications for future research.	11-12
**FUNDING**			
Funding	27	Describe sources of funding for the systematic review and other support (e.g., supply of data); role of funders for the systematic review. This should also include information regarding whether funding has been received from manufacturers of treatments in the network and/or whether some of the authors are content experts with professional conflicts of interest that could affect use of treatments in the network.	12

eAppendix 1. PROSPERO Registration

Registration number: CRD42021274393

Available online: https://www.crd.york.ac.uk/prospero/display_record.php?ID=CRD42021274393

******PROTOCOL******

Comparative effects of antipsychotics on metabolic and endocrine function in children and young people with early onset psychosis, predictors of metabolic dysregulation, and association with psychopathology: a systematic review and network meta-analysis

Review question

1. What is the degree of metabolic dysregulation (defined as alterations in fasting glucose, total cholesterol, low-density lipoprotein (LDL)-cholesterol, high-density lipoprotein (HDL)-cholesterol, triglyceride and prolactin (PRL) levels) and alterations in body weight and body mass index related with short-term ('acute') antipsychotic treatment in children and adolescents with schizophrenia?
2. Can we predict the degree of antipsychotic related metabolic dysregulation from baseline physiology (e.g. body weight) of patients?
3. Is there any association between metabolic dysregulation over time with alterations in degree of psychopathology?

Searches

We plan to search EMBASE, PsycINFO, and MEDLINE from inception using the Results from 21 May 2021

1 (Acepromazine or Acetophenazine or Amisulpride or Aripiprazole or Asenapine or Benperidol or Blonanserin or Bromperidol or Butaperazine or Carpipramine or Chlorproethazine or Chlorpromazine or Chlorprothixene or Clocapramine or Clopenthixol or Clopentixol or Clothiapine or Clotiapine or Clozapine or Cyamemazine or Cyamepromazine or Dixyrazine or Droperidol or Fluanisone or Flupehenazine or Flupenthixol or Flupentixol or Fluphenazine or Fluspirilen or Fluspirilene or Haloperidol or Iloperidone or Levomepromazine or Levosulpiride or Lithium or Loxapine or Loxapinsuccinate or Lurasidone or Melperone or Mepazine or Mesoridazine or Methotrimeprazine or Molindone or Moperone or Mosapramine or Olanzapine or Oxypertine or Paliperidone or Penfluridol or Perazine or Periciazine or Pericyazine or Perospirone or Perphenazine or Pimozide or Pipamperone or Pipothiazine or Pipotiazine or Prochlorperazine or Promazine or Promethazine or Prothipendyl or Quetiapine or Remoxipiride or Reserpine or Riospirone or Risperdal or Risperidone or Seroquel or Sertindole or Stelazine or Sulpiride or Sultopride or Thiopropazate or Thioproperazine or Thioridazine or Tiospirone or Thiothixene or Tiapride or Tiotixene or Trifluoperazine or Trifluperidol or trifluoperidol or Triflupromazine or trifluperazine or Veralipride or Ziprasidone or Zotepine or Zuclopenthixol)

2 (Antipsychoti$ or Anti-psychotic$ or Neurolepic$ or Neurolept$).

3 schizo$.

4 psychosis.

5 psychotic$.

6 Psychoses

7 (early onset or early-onset)

8 (adolesc* or child* or boy* or girl* or juvenil* or minors or paediatri* or pediatri* or pubescen* or school* or student* or teen* or young or youth* or preschool or pre-school)

9 randomized controlled trial

10 clinical trial

11 controlled clinical trial

12 (clinic$ adj2 trial).

13 (random$ adj5 control$ adj5 trial$)

14 (crossover or cross-over)

15 ((singl$ or double$ or trebl$ or tripl$) adj (blind$ or mask$))

16 randomi$.mp.

17 (random$ adj5 (assign$ or allocat$ or assort$ or reciev$))

Types of study to be included

RCT

Condition or domain being studied

Alterations in metabolic parameters (glucose, total cholesterol, Low Density Lipoprotein (LDL) cholesterol, High Density Lipoprotein (HDL) cholesterol, and triglyceride levels), body weight, body mass index.

Participants/population

Children and adolescents with schizophrenia and related psychoses defined according to standard operationalised diagnostic criteria (Feighner criteria, Research Diagnostic Criteria, DSM-III, DSM-III-R, DSM-IV, DSM-V, and ICD-10).

Intervention(s), exposure(s)

Monotherapy with antipsychotic or placebo with either oral or parental administration. No limits will be employed on antipsychotic dose, as there is no clear evidence that dose of antipsychotic treatment influences the degree of metabolic dysregulation, although potential influence of antipsychotic dose on metabolic change will be examined using meta-regression (see 'Meta-regression Analyses' section)1.

Comparator(s)/control

Monotherapy with antipsychotic or placebo.

Context

It has been suggested that treatment with some antipsychotics can lead to glucose dysregulation and lipid disturbance, which can subsequently contribute to development of the metabolic syndrome in children and adolescents with schizophrenia. Moreover, predictors of metabolic dysregulation at baseline are not well defined, and there is no clear association between changes in metabolic parameters and psychopathology.

Main outcome(s)

The aim is to collect data for each study examining mean and standard deviation of change (i.e. from baseline to study endpoint) in the following outcomes:

1. Glucose (mmol/L)
2. Total cholesterol (mmol/L)
3. Low Density Lipoprotein (LDL) cholesterol (mmol/L)
4. High Density Lipoprotein (HDL) cholesterol (mmol/L)
5. Triglycerides (mmol/L)
6. Body weight (kg)
7. Body Mass Index (BMI, kg/m2)
8. Prolactin (PRL) (ng/ml)

All metabolic outcomes will be measured from serum or plasma blood samples taken under fasting conditions.

Additional outcome(s)

None

Data extraction (selection and coding)

We will comply to recommendations of the Preferred Reporting Items for Systematic Reviews and Meta-analyses (PRISMA) extension statement for network meta-analysis.

We will extract the following information name of first author, year of publication, antipsychotic used in study, average dose of antipsychotic used, type of symptom scale used, patient characteristics including age, %male, %Caucasian, duration of drug intervention, mean ± standard deviation, change in symptom scores between baseline and study endpoint, mean ± SD metabolic parameter concentrations (glucose, prolactin, total/LDL/HDL cholesterol/triglycerides) and body weight/body mass index at baseline, active drug number, placebo number.

We may extract data from related publications that refer to the same study. When we encounter unreported data that are required for our analysis, we will contact corresponding authors to request additional data.

Reasonable homogeneity is required for network meta-analysis; therefore we will focus on acute treatment, which will be defined as 6-weeks duration. If data of this duration are not available, we will give preference to the datapoint closest to 6-weeks. To maintain homogeneity in the sample, we will exclude adult studies (i.e.

studies where participant age >18 years). No limits will be employed on antipsychotic dose, as there is no current clear evidence that dose of antipsychotic medication can affect the degree of metabolic dysregulation, although we will investigate the potential influence of antipsychotic dose on metabolic change using meta-regression. Where studies report multiple doses of a single antipsychotic, to increase statistical power, and again based on the lack of clear evidence that antipsychotic dose influences metabolic outcomes, we will calculate a single weighted mean and standard deviation for each metabolic parameter pertaining to a given multi-arm study, applying formulae as previously described by the Cochrane collaboration.

Moreover, we will include data from clinical trials registry relating to papers identified in the literature review.

Risk of bias (quality) assessment

Using the Cochrane Collaboration's Tool for Assessing Risk of Bias, we will assess the risk of bias of individual studies, considering the following domains: randomization sequence generation; allocation concealment; blinding of participants/trial personnel/outcome assessment; incomplete outcome data; and selective outcome reporting.

We will employ the 'Confidence in Network Meta-Analysis' (CINeMA) application to evaluate the credibility of findings from each network meta-analysis. As part of the CINeMA evaluation process, we will need to assess the risk of bias for each study with each study categorised as at low, unclear, or high risk of bias. Our Cochrane Risk of Bias Assessment Tool ratings will be converted into CINeMA categories as follows:

Cochrane Risk of Bias Assessment Tool Rating CINeMA risk of bias category
All domains categorised as low risk Low risk (1)
All domains categorised as either low risk or unclear risk Unclear/moderate risk (2)
Any domain categorised as high risk High risk (3)

Strategy for data synthesis

Characteristics of included studies

Study population characteristics across all eligible trials will be reported, describing the types of comparisons (i.e. which metabolic parameter is examined), as well as physiological/demographic variables (including age, gender (%male), ethnicity (%Caucasian), duration of drug intervention.

Pairwise meta-analyses

For each pairwise comparison with ≥10 studies, data will be compiled to obtain summary mean differences with accompanying 95% confidence interval using a random effects model. We will conduct all the analyses using the metaphor package in the statistical programming language R (version 3.5.1). We will assess small study effects and publication bias for each pairwise comparison by visual inspection of the contour-enhanced funnel plot and

by performing Egger's test of the intercept. For pairwise comparisons informed by 6 or more studies, we will do a quantile-quantile (QQ) plot, to assess whether the random effects in our meta-analyses deviate from a normal distribution.

Assessment of the transitivity assumption

To assess the transitivity assumption, we will restrict our analyses to: 1.children and adolescents with schizophrenia and related psychoses (i.e. we will not examine the metabolic effects of antipsychotics in other patient groups), 2. studies that only investigate acute treatment (aiming for treatment duration of 6-weeks). In addition, we will exclude studies examining patients > 18 years old.There is evidence that age, gender, and ethnicity are parameters that can influence metabolic change in the general population. Therefore, we will investigate if age, gender (% male) and ethnicity (% Caucasian) of participants have a similar distribution across the different treatment comparisons.

Network Analyses

We will perform a random effects network meta-analysis (NMA) to compile all evidence for each outcome if there is similar distribution of age, gender, and ethnicity across collected studies. A frequentist approach to NMA using 'netmeta' will be used.

Assessments of heterogeneity and inconsistency

Monitoring of the I^2 statistic will be used to assess heterogeneity of each network.

Meta-regression analyses

we will examine if body weight/BMI, age, gender, and ethnicity influence metabolic function are linked to change in metabolic parameters.

Analysis of subgroups or subsets

None

Contact details for further information

MARIA ROGDAKI
maria.rogdaki@kcl.ac.uk

Organisational affiliation of the review

Kings College London

Review team members and their organisational affiliations

Dr MARIA ROGDAKI. Kings College London
Dr laurence telesia. kings college
Dr toby pillinger. ioppn

Type and method of review

Meta-analysis, Systematic review

Anticipated or actual start date

02 August 2021

Anticipated completion date

31 January 2023

Funding sources/sponsors

Locally funded lectureship

Conflicts of interest

Language

English

Country

England

Stage of review

Review Ongoing

Subject index terms status

Subject indexing assigned by CRD

Subject index terms

Adolescent; Antipsychotic Agents; Biochemical Phenomena; Child; Humans; Network Meta-Analysis; Psychopathology; Psychotic Disorders

Date of registration in PROSPERO

24 August 2021

Date of first submission

19 August 2021

Protocol Amendment

During review process, authors decided to broaden the inclusion criteria and to include randomised studies in neuropsychiatric and neurodevelopmental disorders, and to gather data on all physiological parameters, not just the ones listed in the protocol.

Supplementary methods

Incorporation of multi-arm studies

For multi-arm studies reporting several doses of an antidepressant, a summary value for a given physiological parameter for all doses was calculated, as follows:

Where multiple doses of a single antidepressant are reported, to increase statistical power, a single weighted mean and standard deviation for each physiological parameter pertaining to a given multi-arm study will be calculated, as follows:

Weighted mean of multiple study arms:

$$\bar{x} = \frac{\sum_{i=1}^{N} n_i x_i}{\sum_{i=1}^{N} n_i}$$

Where,

N = number of observations (i.e. number of arms of the study)

n_i = sample size of study arm

x_i = sample mean of study arm

Weighted standard deviation for 2 study arms:

$$s = \sqrt{\frac{(n_1 - 1)sd_1^2 + (n_2 - 1)sd_2^2 + \frac{n_1 n_2}{n_1 + n_2}(x_1^2 + x_2^2 - 2x_1 x_2)}{n_1 + n_2 - 1}}$$

Where,

n_1 and n_2 = sample sizes of study arms 1 and 2

x_1 and x_2 = means of study arms 1 and 2

sd_1 and sd_2 = standard deviations of study arms 1 and 2

Where there are more than 2 study arms to combine standard deviations, the above formula will be applied sequentially (i.e. combining study arm 1 and 2 to create arm ‘1+2’, then combining group ‘1+2’ and group 3 to create group ‘1+2+3’ and so on).

Calculation of standard deviation of change score when only baseline and follow-up data available

When a t test comparing baseline and follow-up data was performed and a P-value was provided, we would obtain the corresponding t value from a table of the t distribution. The standard error would then be calculated by dividing the difference in means by the t value. We assumed that the standard error was the same in both intervention groups. Standard deviation would then be calculated by dividing the standard error by the square route of the sum of the reciprocals of the two sample sizes.

P-score calculation

We used P-scores to rank antidepressants based on degree of physiological dysregulation. P-scores range from 0 to 1, with a higher score indicating that a given treatment is 'better' than competing treatments. It is arguable that both extremely high and low levels of physiological parameters are pathological; for the purposes of these analyses, we argued that lower cardiometabolic values were preferable with the exception of HDL-cholesterol, where higher levels were deemed preferable.

eAppendix 2. Methods employed to assess confidence in NMA results using CINeMA

1. **Within-study bias**
   Defined as per Cochrane risk of bias assessment as follows:

Cochrane Risk of Bias Assessment Tool Rating	CINeMA risk of bias category
All domains categorised as low risk	Low risk (1)
Domains categorised as either low risk or unclear risk	Unclear/moderate risk (2)
Any domain categorised as high risk	High risk (3)

2. **Across-studies bias**
   Where pairwise meta-analyses are possible, grade risk of bias based on funnel plot/Egger's regression test. If <10 studies for a given pairwise comparison, downgrade confidence.
3. **Indirectness**
   No indirectness assumed
4. **Imprecision**
   A clinically meaningful threshold was set as +/-5% of a given physiological parameter. We used data assuming a patient age of 15 years. For weight we used the 50th percentile, as defined by the Centers for Disease Control and Prevention (https://www.cdc.gov/index.htm) and calculated the average between male and female values. Where a reference range was provided, we used the midpoint between the upper and lower values for that range. Where ranges were different for males and females, the most extreme upper/lower values were selected for midpoint calculation. Thus, the following values were used:

Weight: 55.5kg (5% =2.78kg)
BMI: 20.1kg/m^2 (5% = 1.01 kg/m^2)
Triglycerides: <1.7mmol/L (5% upper limit: 0.085mmol/L)
Prolactin: <22.5ng/ml (5% upper limit: 1.125ng/mL)
Glucose: 3.9-5.6mmol/L (5% of mid-point: 0.085mmol/L)
Total cholesterol: <5mmol/L (5% of upper limit: 0.25mmol/L)
HDL cholesterol: >1.1mmol/L (5% of lower limit: 0.05mmol/L)
LDL cholesterol: <3mmol/L (5% of upper limit:0.15mmol/L)
Systolic blood pressure: 120mmHg (5% of upper limit: 6mmHg)
Heart rate: 60-100bpm (5% of mid-point: 4bpm)
QTc: calculated as SMD, therefore effect size of 0.2 selected (smallest effect as per Cohen)

5. **Heterogeneity**
   Use recommendations automatically provided by CINeMA
6. **Incoherence**
   Downgrade evidence if only indirect evidence

Interpretation of CINeMA Report to give overall confidence rating:

- All assessments deemed 'no concerns' = 'high confidence' (coded as green)
- Combination of 'no concerns' and 'some concerns' but greater proportion of 'no concerns' = 'moderate confidence' (coded as blue)
- Combination of 'no concerns' and 'some concerns' but greater proportion of 'some concerns' = 'low confidence' (coded as yellow)
- Any 'major concerns' = 'very low confidence' (coded as red)

'Indirectness' and 'incoherence' are assessed together; also, 'imprecision' and 'heterogeneity' are assessed together. Where there is contradiction in the confidence ratings for the two assessments making up these respective pairs, the following conversion tool will be used and new confidence rating applied to both parameters in the pair:

'No concern' + 'Some concern' = 'Some concern' for both parameters
'No concern' + 'Major concern' = 'Some concern' for both parameters
'Some concern' + 'Major concern' = 'Major concern' for both parameters

eAppendix 3. Metaregression

Using the 'metafor' package (version 3.8-1),[1] we conducted random-effects meta-regressions of placebo-controlled trials to examine the relationship between antipsychotic-associated physiological change (the outcome variable) and potential effect modifiers (baseline body weight, age, sex, and ethnicity) using the rma() function. This model assumes a normal distribution for the effect sizes and the distribution of the random effect term (τ^2). We drew quantile-quantile (QQ) plots, to assess whether the random effects in meta-analyses deviated from a normal distribution

eAppendix 4. Assessing the relationship between alterations in metabolic parameters and psychopathology

We aimed to perform a bivariate meta-analysis of the MD for change in weight/BMI/metabolic parameter/endocrine parameter and SMD for change in total-symptoms (assessed using PANSS) in patients with schizophrenia. We predicted there would be no report of within-study correlations between the outcomes, therefore we planned to use a model proposed by Riley and colleagues which overcomes this problem, using the package 'metamisc' (v0.2.0)[2].

eTable 2 Studies included in the meta-analysis.

Please note, a full list of excluded studies can be made available on request to the authors.

Study	Country/ Participant population	Diagnosis	Age range (years)	Physical health exclusion criteria	Intervention	Antipsychotic mean dose (mg)	Sample size	Trial duration (weeks)	Parameters tested
Aman 2002[3]	USA	Disruptive behaviours in children with subaverage intelligence	5-12	head injury as a cause of intellectual disability; a seizure disorder re- quiring medication; a history of tardive dyskinesia or neuroleptic malignant syndrome; serious or progressive illnesses; the presence of human immunodeficiency virus; laboratory values outside the normal range	Risperidone<br>Placebo	1.16	55<br>63	6	Weight<br>PRL
Buitelaar 2001[4]	Netherlands	Aggression in adolescents with subaverage cognitive abilities	12-18	neurologic, cardiac, pulmonary, or hepatic diseases; comorbid substance abuse disorder according to DSM-IV;	Risperidone<br>Placebo	3	19<br>19	6	PRL<br>QTc
Connor 2008[5]	USA	Conduct Disorder	12-17	alcohol or substance abuse or dependence within 3 months of study entry; current or past history of lenticular abnormality or juvenile cataracts; seizure disorder;	Quetiapine<br>Placebo	294	9<br>10	7	PRL<br>QTc
DelBello 2009[6]	USA	Bipolar Affective Disorder	12-18	a substance use disorder (other than nicotine) within the previous three months; an unstable medical or neurological illness;	Quetiapine<br>Placebo	403	17<br>15	8	Weight<br>BMI<br>HDL<br>TG<br>HR<br>SBP
DelBello 2017[7]	USA	Bipolar Affective Disorder	10-17	clinically significant neuro-logic, endocrine, or other medical disorder was present at screening that might pose a risk to patients participating in the study or that might confound interpretation of study results.	Lurasidone<br>Placebo	33.6	175<br>172	6	Weight<br>BMI<br>LDL<br>TC<br>TG<br>PRL<br>Glu

Findling 2000[8]	USA	Conduct Disorder	5-15	clinically significant general medical condition, organic mental syndromes	Risperidone<br>Placebo	1.5	10<br>10	10	Weight
Findling 2008[9]	USA, Europe, South America, Asia, the Caribbean and South Africa	Schizophrenia	13-17	neurological disorder, severe head trauma, unstable medical condition	Aripiprazole<br>Placebo	20	196<br>98	6	Weight<br>TG<br>HDL<br>TC<br>Glu<br>QTc
Findling 2009[10]	USA	Bipolar I Disorder	10-17	substance or alcohol use disorder, positive drug screen for cocaine or other substances of abuse during screening; other medical reason as determined by the investigator.<br>neuroleptic malignant syndrome clinically important laboratory test results, vital sign, or electrocardiogram (ECG) abnormali ties; diabetes mellitus; abnormally elevated serum glucose levels; epilepsy; history of severe head trauma; stroke; unstable thyroid pathology requiring treatment; other unstable medical conditions.	Ariprazole<br>Placebo	20	197<br>97	4	Weight<br>BMI<br>PRL
Findling 2012[11]	Asia, Central and Eastern Europe, South Africa, USA	Schizophrenia	13-17	Laboratory tests outside the normal range, hospital admission for diabetes, or diabetes related illness in the past 3 months, renal, cardiovascular, hepatic, haematological,	Quetiapine<br>Placebo	600	127<br>73	6	Weight<br>TG<br>HDL<br>LDL<br>TC<br>Glu<br>PRL

				endocrinologic, ophthalmologic or other medical conditions that were unstable, pregnancy and lactation					
Findling 2013a[12]	USA	Bipolar I disorder	10-17	a Fridericia-corrected QT interval (QTcF) ‡460 ms, DSM-IV substance abuse/dependence (except nicotine or caffeine) in the preceding month, and numerous other standard medical and psychiatric exclusion criteria.	Ziprasidone<br>Placebo	94	149<br>88	4	Weight<br>LDL<br>TC<br>TG<br>Glu
Findling 2013b[13]	USA, Russia, Ukraine, India, Malaysia, Singapore, Peru, Columbia, Costa Rica	Schizophrenia	13-17	Serious/unstable medical condition, history of significant cardiovascular disease, cardiac arrythmias, conduction abnormalities prolongation, significant ECG abnormalities, QTc>460ms	Ziprasidone<br>Placebo	80-160	135<br>52	6	Weight<br>TG<br>HDL<br>LDL<br>TC<br>Glu<br>HR<br>QTc<br>SBP
Findling 2014[14]	Colombia, India, Mexico, Serbia, South Africa, Taiwan, USA	Bipolar Depression	10-17	clinically significant deviations from normal reference ranges of clinical laboratory parameters.	Quetiapine<br>Placebo	204.9	92<br>100	8	Weight<br>HDL<br>LDL<br>TC<br>TG<br>PRL<br>Glu
Findling 2015[15]	USA, Mexico, Russia, Ukraine, Serbia, Romania, Croatia, Bosnia-Herzegovina, South Africa, South	Schizophrenia	12-17	Uncontrolled/unstable medical condition; abnormal laboratory, vital signs, physical examination; ECG; unstable diabetes; significantly abnormal glucose at baseline	Asenapine<br>Placebo	3.75	204<br>102	8	Weight<br>TG<br>TC<br>Glu<br>PRL

	Korea, India, Columbia								
Findling 2015[16]	USA, Russia	Manic or Mixed Episode bipolar disorder	10-17	uncontrolled, unstable, clinically significant medical condition.	Asenapine<br>Placebo	6.75	302<br>101	3	Weight<br>BMI<br>TC<br>TG<br>PRL<br>Glu
Findling 2017[17]	USA	Cyclothymia/BPAD	5-17	active neurological/medical disorder for which treatment with APZ or another atypical anti- psychotic would be contraindicated; general medical/neurological condition (including clinically significant abnormalities on screening laboratories) that may be considered to be the aetiology of the patient's mood disorder; general medical/neurological condition that could interfere with the interpretation of clinical response to APZ;	Aripiprazole<br>Placebo	7.1	30<br>29	12	Weight<br>BMI<br>TC<br>TG<br>PRL<br>Glu<br>SBP
Findling 2022[18]	USA	Mania in bipolar I disorder	10-17	a Fridericia-corrected QT interval (QTcF) ‡450 msec at screening or baseline; a history of significant cardiac arrhythmias; conduction abnormalities; a known personal history of QTc prolongation (including congenital long QT syndrome), or history of significant	Ziprasidone<br>Placebo	66.15	86<br>85	4	Weight<br>BMI<br>HDL<br>LDL<br>TC<br>TG<br>Glu

				cardiovascular disease; clinically significant neurologic, endocrine, or medical disorders that could affect the study results or patient safety were excluded.					
Gilbert 2004[19]	USA	Tourette's syndrome or Tic disorder	7-17	serious or unstable medical illness (e.g., diabetes) or with abnormal ECG or laboratory findings	Risperidone<br>Pimozide	2.5<br>2.4	13<br>13	4	Weight<br>QTc
Goldman 2017 [20]	USA, Mexico, Russia, Ukraine, Bulgaria, Romania, Spain, France, Hungary, Philippines, Columbia, Korea, Malaysia	Schizophrenia	13-17	Evidence of severe or moderate movement disorder	Lurasidone<br>Placebo	60	214<br>112	6	Weight<br>TG<br>TC<br>LDL<br>GLU<br>PRL
Hass 2009a [21]	India, Russia, Ukraine, USA	Schizophrenia	13-17	History of NMS	Risperidone<br>Placebo	3.5	106<br>54	6	PRL
Haas 2009b[22]	USA	Mania in bipolar disorder	10-17	Not medically stable	Risperidone<br>Placebo	3	111<br>58	3	Weight<br>BMI<br>TC<br>TG<br>PRL<br>Glu
Hagman 2011[23]	USA	Anorexia nervosa	12-20	active hepatic or renal disease	Risperidone<br>Placebo	2.5	18<br>22	9	TG<br>TC<br>PRL<br>Glu<br>QTc
Ichikawa 2017[24]	Japan	ASD	6-17	Rett syndrome	Aripiprazole<br>Placebo	5.7	47<br>45	8	Weight<br>BMI

									TG<br>PRL<br>Glu
Kent 2013[25]	USA	Autism	5-17	Neurologic disorders; moderate or severe extrapyramidal symptoms or tardive dyskinesia	Risperidone<br>Placebo	0.9375	61<br>35	6	Weight<br>BMI<br>TG<br>HDL<br>LDL<br>TC<br>PRL
Kowach 2015[26]	USA	Bipolar Disorder	3-8	Clinically significant or unstable hepatic, renal, gastroenterological, respiratory, cardiovascular, endocrine, immunological, hematological, or other systemic medical conditions; neurological disorders including epilepsy, stroke, or severe head trauma; clinically significant laboratory abnormalities on complete blood count (CBC) with differential, electrolytes, blood urea nitrogen (BUN), creatinine, hepatic transaminases, urinalysis, thyroid indices (T3, total T4, tree T4, thyroid-stimulating hormone [TSH]) and electrocardiogram (ECG); evidence of fetal alcohol syndrome or an alcohol-related neurodevelopmental dis- order	Risperidone<br>Placebo	0.5	18<br>7	6	Weight<br>BMI<br>TG<br>HDL<br>LDL<br>TC<br>PRL

Lamberti 2016[27]	Italy	ADHD symptoms in ASD and ADHD	6-13	neurological diseases (neurogenetic diseases, epilepsy, brain injuries, or cerebral lesions documented by magnetic resonance imaging)	Risperidone<br>Aripiprazole	3<br>15	22<br>22	12	Weight<br>Glu<br>Weight<br>SBP<br>HR<br>PRL<br>Glu<br>HR<br>SBP
Loebel 2016[28]	USA	Irritability in ASD	6-17	History of seizures	Lurasidone<br>Placebo	40	100<br>49	6	Weight<br>BMI<br>QTc<br>PRL
Kryzhanovskaya 2009 [29]	USA, Russia	Schizophrenia	13-17	Acute/unstable medical condition	Olanzapine<br>Placebo	12.6	49<br>15	6	Weight<br>BMI<br>TC<br>TG<br>HDL<br>LDL<br>Glu<br>PRL<br>QTc
Kumra 1996[30]	USA	Schizophrenia	6-18	Neurological/medical disease	Clozapine<br>Haloperidol	239<br>16	10<br>11	6	Weight<br>SBP
Kumra 2008[31]	USA	Schizophrenia or schizoaffective disorder	10-18	Serious/unstable medical condition	Clozapine<br>Olanzapine	487.5<br>26.2	18<br>21	12	BMI<br>TG<br>TC<br>Glucose
Marcus 2009[32]	USA	Irritability in children and young people with autistic disorder	6-17	history of neuroleptic malignant syndrome; seizure in the past year; history of severe head trauma or stroke; history or current evidence of any unstable medical	Aripiprazole<br>Placebo	7.5	166<br>52	8	Weight<br>BMI

				conditions; or an abnormal laboratory test result, considered clinically significant vital sign result, or electrocardiogram (ECG) finding considered clinically significant.					
McCracken 2002[33]	USA	Autism and serious behavioural problems	5-17	Serious medical/neurological disorders	Risperidone<br>Placebo	2.4	49<br>52	8	Weight
Miral 2008[34]	Turkey	Autistic Disorder	8-18	Epilepsy	Risperidone<br>Haloperidol	2.6<br>2.6	13<br>15	12	Weight<br>HR<br>SBP
Nicol 2018[35]	USA	Axis I *DSM IV-TR* diagnosis and clinically significant aggression defined by a score of at least 18 on the Irritability subscale of the Aberrant Behavior Checklist	6-18	substance use disorders<br>diabetes	Aripiprazole<br>Olanzapine<br>Risperidone	6<br>6.3<br>1	49<br>46	12	Weight<br>HDL<br>LDL<br>TC<br>TG<br>Glu
Pagsberg 2017 [36]	Denmark	Schizophrenia spectrum disorder, delusional disorder, affective spectrum psychotic disorder	12-17	Compulsory treatment<br>Worsening of clinical state during the trial<br>Organic psychosis<br>Severe somatic illness<br>History of severe head trauma	Quetiapine ER<br>Aripiprazole	451.82<br>14.61	55<br>58	12	Weight<br>BMI<br>TG<br>TC<br>HDL<br>LDL<br>Glu<br>HR<br>QTc

									SBP
Pathak 2013[37]	USA	Mania with bipolar 1 disorder	10-17		Quetiapine<br>Placebo	500	193<br>90	3	Weight<br>HDL<br>LDL<br>TC<br>TG<br>PRL<br>Glu
Safavi 2016[38]	Iran	Disruptive behaviour and ADHD	3-6	Chronic physical or neurological disorder	Risperidone<br>Aripiprazole	2<br>10	20<br>20	8	Weight
Saito 2022[39]	Japan	Schizophrenia	12-18	NMS, TD, paralytic ileus, rhabdomyolysis, agranulocytosis, pulmonary embolism, or deep vein thrombosis; Parkinson's disease; diabetes mellitus; complications such as serious cardiovascular, liver, kidney, organic brain, haematological, endocrinal, or spastic disease; a history of substance abuse or dependence and alcohol abuse or dependence	Blonanserin<br>Blonaserin<br><br>Placebo	8<br>16	51<br>52<br>47	6	Weight<br>BMI<br>TG<br>TC<br>Glu<br>PRL
Sallee 2000[40]	USA	Tourette's syndrome	7-17	Abnormal laboratory tests, Neuroleptic Malignant Syndrome	Ziprasidone<br>Placebo	28.2	16<br>11	8	Weight
Sallee 2017[41]	USA, Canada, Hungary, and Italy.	Tourette's syndrome	7-17	other neurologic disorders that may have accompanying abnormal movements; those with a psychoactive-substance use disorder within the previous three months (DSM-IV-TR) and/or a positive drug screen.	Aripiprazole<br>Placebo	10	89<br>88	8	Weight<br>PRL

Savitz 2015 [42]	India, Romania, Russia, Slovakia, Spain, Ukraine, USA	Schizophrenia	12-17	History of seizure; TD, encephalopathic syndrome; increased risk for torsade de pointes; insulin dependent diabetes mellitus	Paliperidone<br>Aripiprazole	6.75<br>11.56	112<br>114	8	Weight<br>BMI<br>TC<br>TG<br>LDL<br>Glu<br>PRL
Shaw 2006 [43]	USA	Schizophrenia	7-16	Nil stated	Olanzapine<br>Clozapine	18.1<br>327	12<br>13	8	Weight, BMI
Shea 2004[44]	Canada	ASD and other pervasive developmental disorders	5-12	clinically significant laboratory abnormalities, or a seizure disorder for which they were receiving 1 anticonvulsant or if they had had a seizure in the last 3 months; tardive dyskinesia; neuroleptic malignant syndrome; drug or alcohol abuse, or human immunodeficiency virus infection were excluded.	Risperidone<br>Placebo	1.48	40<br>39	8	Weight<br>HR<br>SBP
Sikich 2008[45]	USA	Schizophrenia, Schizoaffective, schizophreniform disorder	8-19	Nil stated	Olanzapine<br>Risperidone<br>Molindone	11.4<br>2.8<br>59.9	40<br>35<br>41	8	Weight<br>BMI<br>TC<br>LDL<br>HDL<br>TG<br>Glu<br>PRL<br>QTc
Singh 2011[46]	India, Romania, Russia, Ukraine, USA	Schizophrenia	12-17	History of seizure, TD, encephalopathic syndrome, increased risk for torsade de pointes, insulin dependent diabetes mellitus, significant or unstable medical disease	Paliperidone<br>Placebo	Average 5.625	149<br>51	6	Weight

Snyder 2002[47]	USA, Canada	Conduct and Disruptive Behaviour Disorder and Subaverage IQ	5-12	Head injury as a cause of impaired IQ; seizure condition requiring medication; serious or progressive illness or clinically abnormal laboratory values; history of tardive dyskinesia, neuroleptic malignant syndrome, or hypersensitivity to any antipsychotic drug; known presence of human immunodeficiency virus;	Risperidone<br>Placebo	0.98	53<br>57	6	PRL<br>HR
Tohen 2007[48]	United States, Puerto Rico	Bipolar mania	13-17	clinically significant abnormal laboratory values at baseline; DSM-IV-TR substance dependence (except nicotine and caffeine) within the past 30 days.	Olanzapine<br>Placebo	10.7	107<br>54	3	Weight<br>BMI<br>PRL<br>Glu<br>TC<br>HR<br>QTc<br>SBP
Yoo 2013[49]	Korea	Tourette's syndrome	6-18	seizure disorders, a history of neuroleptic malignant syndrome, serious brain injury, stroke, or other neurologic disorders	Aripiprazole<br>Placebo	11	32<br>28	10	Weight<br>BMI<br>HDL<br>LDL<br>TC<br>TG<br>PRL<br>Glu
**Total:** 47 studies									

BMI, body mass index; TC, total cholesterol; LDL, low density lipoprotein cholesterol; HDL , high density lipoprotein cholesterol; TG, triglycerides; Glu, glucose; PRL, prolactin; SBP, systolic blood pressure; HR, heart rate; QTc, corrected QT interval ER, extended release; NMS, neuroleptic malignant syndrome; TD, tardive dyskinesia.

eTable 3. Cochrane risk of bias assessment (low, high, unclear risk of bias)

Study	Random sequence generation	Allocation concealment	Blinding of participants and personnel	Blinding of outcome assessment	Incomplete outcome data addressed	Selective reporting	Overall risk of bias
Aman 2002[3]	unclear	unclear	low	low	low	low	moderate
Buitelaar 2001[4]	low	low	low	low	low	low	low
Connor 2008[5]	unclear	unclear	high	low	low	low	high
DelBello 2009[6]	unclear	unclear	low	low	low	low	moderate
DelBello 2017[7]	low	low	low	low	low	low	low
Findling 2000[8]	low	low	low	low	low	low	low
Findling 2008[9]	low	unclear	low	low	low	low	moderate
Findling 2009[10]	unclear	low	low	low	low	low	moderate
Findling 2012[11]	low	low	low	low	low	low	low
Findling 2013a[12]	low	unclear	low	low	low	low	moderate
Findling 2013b[13]	unclear	unclear	low	unclear	unclear	unclear	moderate
Findling 2014[14]	unclear	unclear	low	low	low	low	moderate
Findling 2015a[15]	low	low	low	low	low	low	low
Findling 2015b[16]	low	low	low	low	low	low	low
Findling 2017[17]	low	unclear	low	low	low	low	moderate
Findling 2022[18]	low	unclear	low	low	low	low	moderate
Gilbert 2004[19]	low	low	low	low	unclear	unclear	moderate
Goldman 2017[20]	low	low	low	low	low	low	low
Haas 2009[21]	unclear	unclear	unclear	unclear	unclear	unclear	moderate
Haas 2009b[22]	unclear	unclear	unclear	unclear	unclear	unclear	moderate
Hagman 2011[23]	low	low	low	low	unclear	unclear	moderate
Ichikawa 2017[24]	low	low	low	low	low	low	low
Kent 2013[25]	low	low	low	low	low	low	low
Kowach 2015[26]	unclear	unclear	low	low	low	low	moderate
Kryzhanovskaya 2009[29]	unclear	unclear	low	unclear	unclear	low	moderate
Kumra 1996[30]	unclear	unclear	unclear	unclear	unclear	unclear	moderate
Kumra 2008[31]	low	low	low	low	low	low	low
Lamberti 2016[27]	unclear	unclear	high	high	high	low	high
Loebel 2016[28]	low	low	low	low	low	low	low
Marcus 2009[32]	low	unclear	low	low	low	low	moderate
McCracken 2002[33]	low	unclear	low	low	low	low	moderate
Miral 2008[34]	unclear	unclear	low	low	low	low	moderate
Nicol 2018[35]	low	low	low	low	low	low	low
Pagberg 2017[36]	low	low	low	low	low	low	low
Pathak 2013[37]	low	low	low	low	low	low	low
Safavi 2016[38]	unclear	unclear	high	unclear	h	unclear	high
Saito[39]	low	low	low	unclear	low	low	low

Sallee 2000[40]	unclear	unclear	low	low	low	low	moderate
Sallee 2017[41]	low	low	low	low	low	low	low
Savitz 2015[42]	low	unclear	low	low	unclear	low	low
Shaw 2006[43]	low	low	low	low	low	low	low
Shea 2004[44]	unclear	unclear	low	low	low	low	moderate
Sikich 2008[45]	low	unclear	low	low	low	low	moderate
Singh 2011[46]	low	low	low	low	unclear	low	moderate
Snyder 2002[47]	unclear	unclear	low	low	low	low	moderate
Tohen 2007[48]	low	low	low	low	low	low	low
Yoo 2013[49]	low	low	low	low	low	low	low

eTable 4. Baseline variables across treatment-comparison

Treatment Comparison	Number of studies	Mean age (years)	% male	% non-Caucasians	Mean Baseline body weight(kg)
**aripiprazole:placebo**	7	15.27	68.46	39.05	54.49
**aripiprazole:quetiapine**	1	15.75	29.97	NaN	62.23
**aripiprazole:risperidone_paliperidone**	4	12.40	70.64	34.41	49.16
**ariprazole: olanzapine**	1	11.36	46.68	25.28	46.68
**asenapine:placebo**	2	14.47	53.95	37.79	60.42
**blonanserin:placebo**	1	15.50	42.68	100	55.39
**clozapine:haloperidol**	1	14.07	57.03	NaN	NaN
**clozapine:olanzapine**	1	15.25	56.09	79.43	NaN
**haloperidol:risperidone**	1	10.48	80.48	N/A	38.01
**lurasidone:placebo**	3	14.04	61.65	27.38	57.17
**molindone:olanzapine**	1	NaN	61.73	34.20	64.24
**molindone:risperidone_paliperidone**	1	NaN	61.29	34.13	63.35
**olanzapine:placebo**	2	15.62	60.70	29.54	67.45
**olanzapine:risperidone_paliperidone**	2	11.20	67.80	46.19	52.407
**pimozide:risperidone**	1	11.00	79.00	5.00	54.90
**placebo:quetiapine**	5	14.13	59.68	30.45	62.09
**placebo:risperidone_paliperidone**	11	11.17	79.29	33.94	34.86
**placebo:ziprasidone**	4	14.02	54.09	27.21	56.73

eAppendix 5. Pairwise meta-analyses

There were sufficient studies for one pairwise comparison: change in weight with placebo and risperidone/paliperidone. Risperidone/paliperidone was associated with increased weight gain compared with

placebo (MD: +1.42kg 95%CI: 0.67 to 2.18, P<0.0001). Tau was 0.98kg (large), I^2 was 86.06% (large), and Egger's regression test did not bear evidence of bias (p=0.81).

eFigure 1. Pairwise meta-analysis forest plot: placebo versus risperidone/paliperidone.

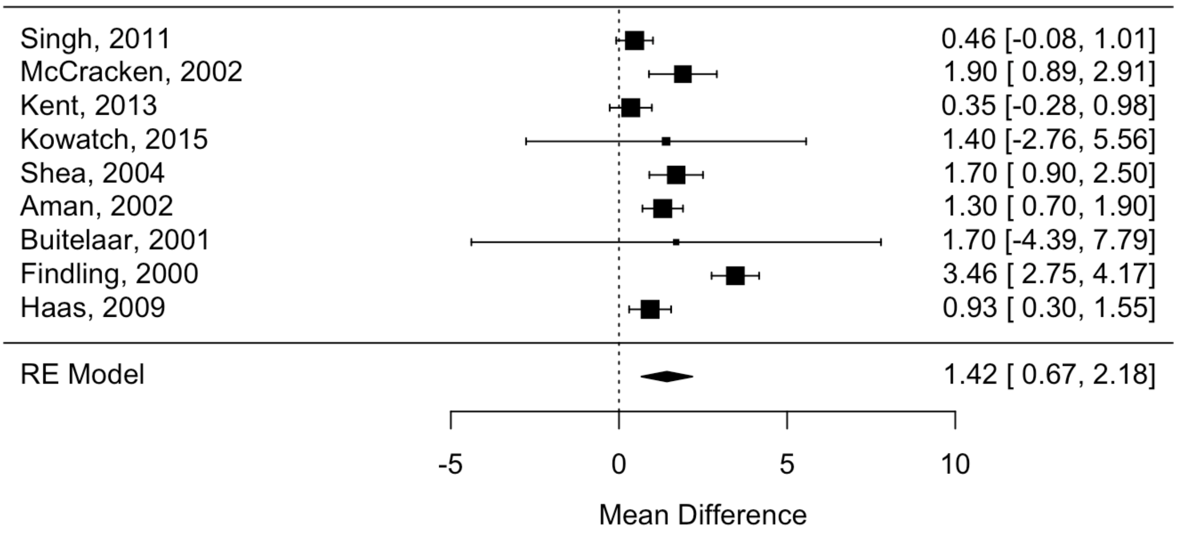

eFigure 2. Pairwise meta-analysis contour-enhanced funnel plot: placebo versus risperidone/paliperidone.

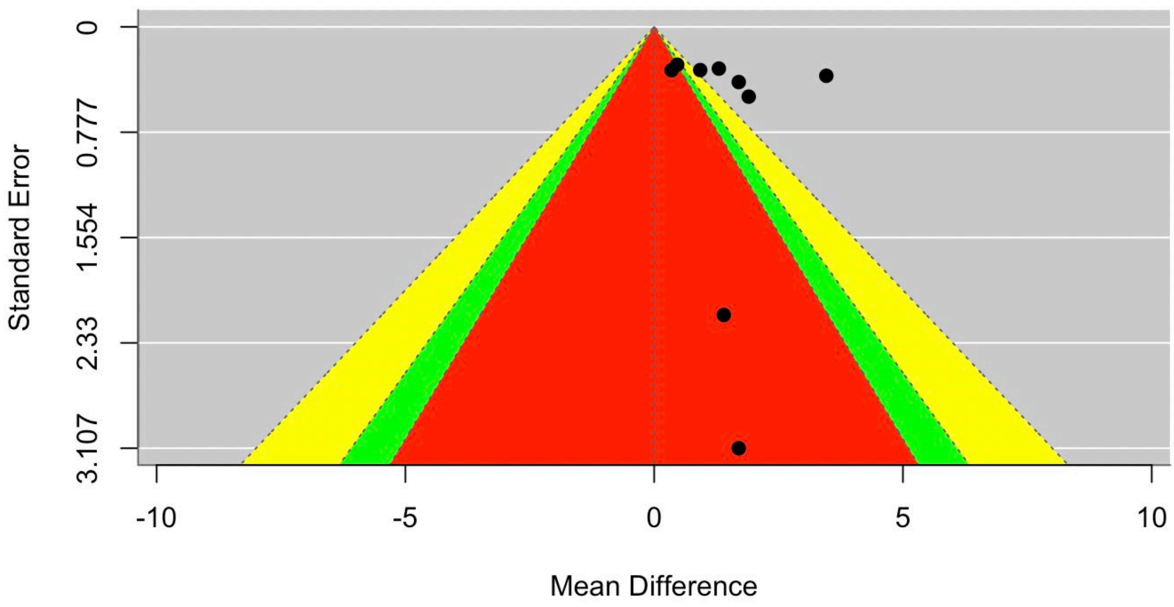

eFigure 3. QQ plots: Change in weight, risperidone/paliperidone vs placebo

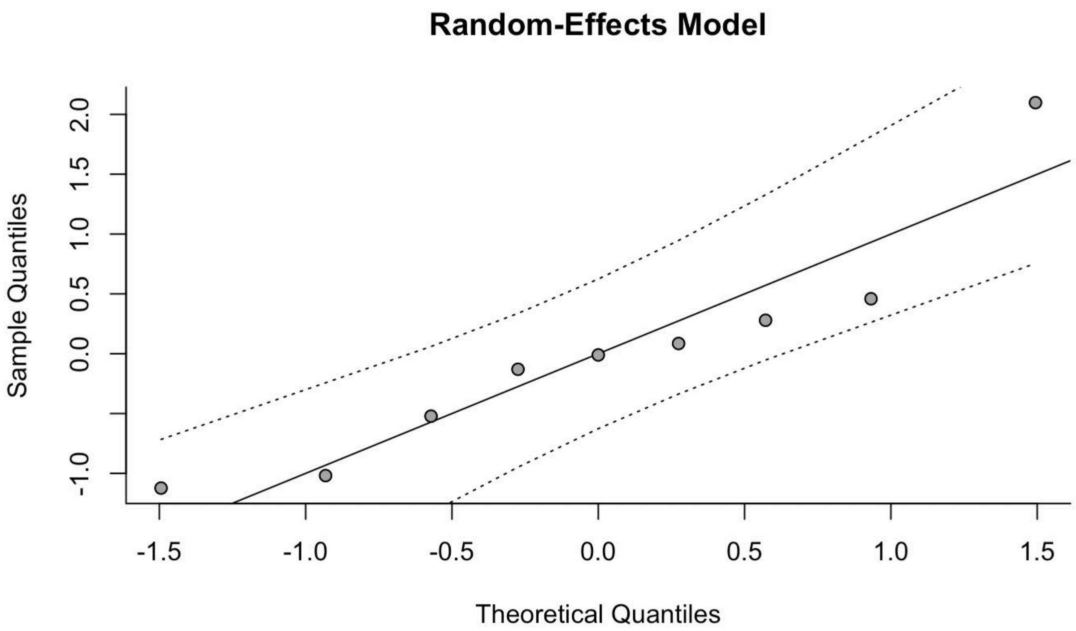

eFigure 4: QQ plots: Change in weight, aripiprazole vs placebo

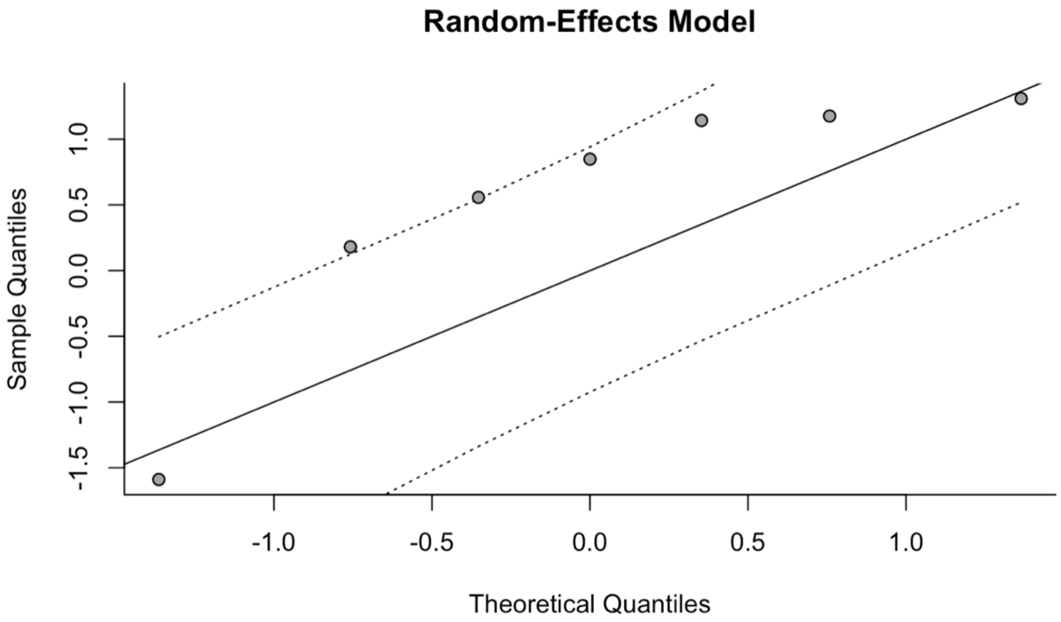

eFigure 5: QQ plots: Change in BMI, aripiprazole vs placebo

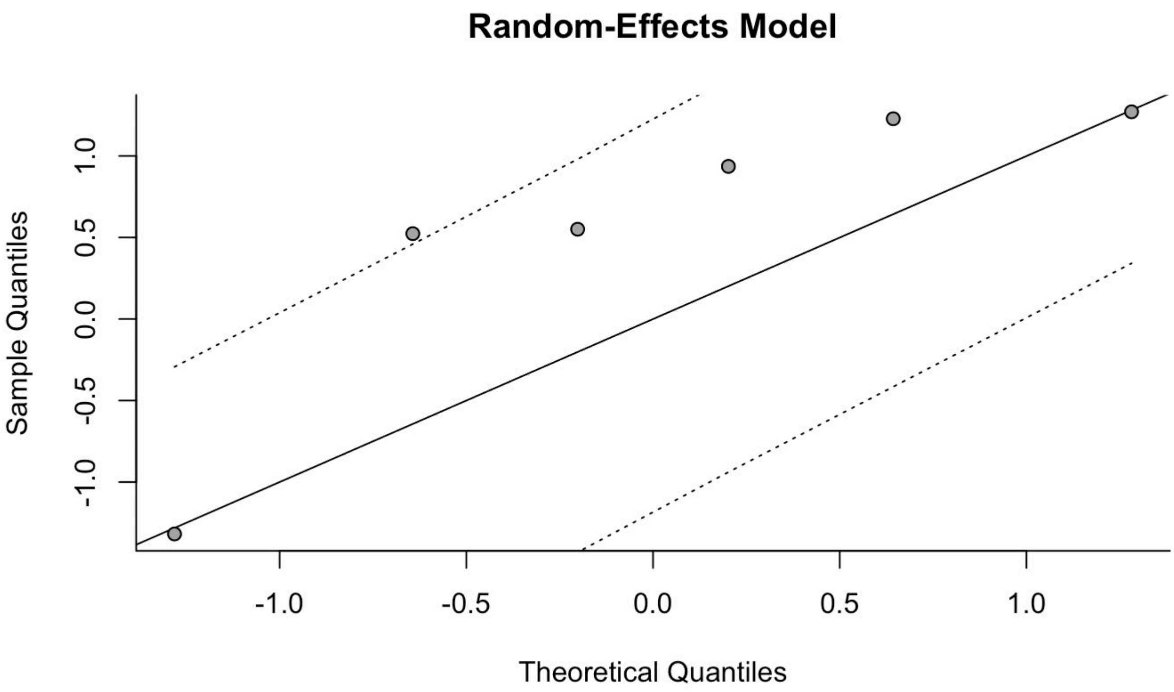

eFigure 6: QQ plots: Change in prolactin, risperidone/paliperidone vs placebo

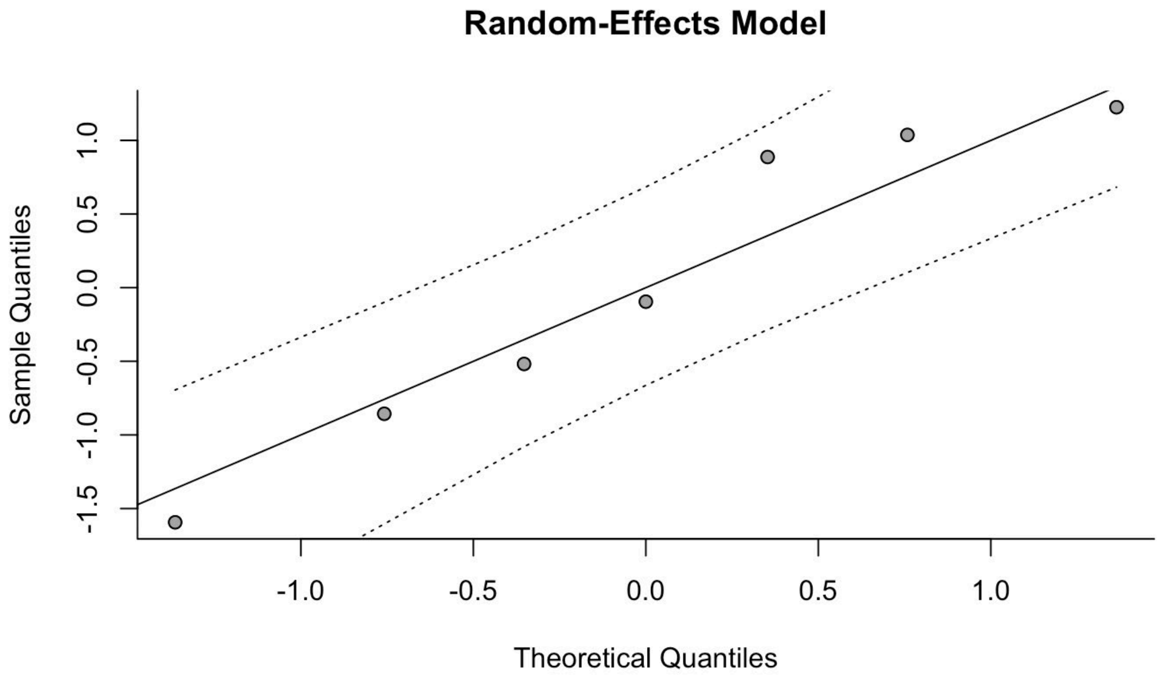

eTable 5. League table for changes in body weight (kg) associated with antipsychotic drug treatment. For interpretation, a number larger than zero favours the column-defining treatment of a cell, i.e this treatment leads to an increase in weight. Values depicted are mean differences with associated 95% confidence **intervals.**

1

**aripiprazole**										
-0.26 (-1.50 to 0.97)	**asenapine**									
0.83 (-0.55 to 2.22)	1.10 (-0.60 to 2.79)	**blonanserin**								
-3.84 (-7.84 to 0.16)	-3.58 (-7.71 to 0.56)	-4.68 (-8.86 to-0.49)	**clozapine**							
-4.75 (-10.10 to 0.60)	-4.49 (-9.94 to 0.97)	-5.58 (-11.07 to-0.10)	-0.91 (-5.08 to 3.26)	**haloperidol**						
0.51 (-0.34 to 1.36)	0.77 (-0.52 to 2.07)	-0.33 (-1.76 to 1.11)	4.35 (0.31 to 8.38)	5.26 (-0.12 to10.63)	**lurasidone**					
2.85 (1.19 to 4.51)	3.11 (1.16 to 5.07)	2.02 (-0.04 to 4.07)	6.69 (2.47 to10.91)	7.60 (2.08 to13.12)	2.34 (0.60 to 4.08)	**molindone**				
-2.89 (-3.78 to-2.01)	-2.63 (-3.99 to-1.26)	-3.73 (-5.23 to-2.22)	0.95 (-2.97 to 4.87)	1.86 (-3.45 to 7.16)	-3.40 (-4.43 to-2.37)	-5.74 (-7.33 to-4.15)	**olanzapine**			
0.19 (-16.77 to17.15)	0.45 (-16.54 to17.44)	-0.64 (-17.64 to16.36)	4.03 (-13.38 to21.44)	4.94 (-12.83 to22.71)	-0.32 (-17.28 to16.65)	-2.66 (-19.68 to14.36)	3.08 (-13.89 to20.05)	**pimozide**		
0.85 (0.31 to 1.39)	1.12 (0.00 to 2.23)	0.02 (-1.26 to 1.30)	4.69 (0.71 to 8.67)	5.60 (0.27 to10.94)	0.34 (-0.32 to 1.00)	-2.00 (-3.61 to-0.39)	3.74 (2.95 to 4.53)	0.66 (-16.29 to17.61)	**placebo**	
-0.55 (-1.35 to 0.26)	-0.28 (-1.55 to 0.99)	-1.38 (-2.79 to 0.03)	3.30 (-0.73 to 7.32)	4.20 (-1.17 to 9.58)	-1.05 (-1.95 to-0.16)	-3.40 (-5.12 to-1.67)	2.35 (1.35 to 3.34)	-0.74 (-17.70 to16.23)	-1.40 (-2.00 to-0.79)	**quetiapine**

-0.51 (-1.06 to 0.05)	-0.25 (-1.44 to 0.94)	-1.34 (-2.69 to 0.00)	3.33 (-0.65 to 7.32)	4.24 (-1.10 to 9.58)	-1.02 (-1.80 to-0.24)	-3.36 (-4.95 to-1.77)	2.38 (1.56 to 3.20)	-0.70 (-17.65 to16.25)	-1.36 (-1.78 to-0.94)	0.04 (-0.70 to 0.77)	**risperidone/paliperidone**	
1.15 (0.29 to 2.01)	1.41 (0.11 to 2.72)	0.32 (-1.13 to 1.76)	4.99 (0.96 to 9.03)	5.90 (0.52 to11.28)	0.64 (-0.30 to 1.59)	-1.70 (-3.45 to 0.05)	4.04 (3.00 to 5.08)	0.96 (-16.01 to17.93)	0.30 (-0.37 to 0.97)	1.70 (0.79 to 2.60)	1.66 (0.87 to 2.45)	**ziprasidone**

eTable 6. League table for changes in body mass index (BMI, kg/m2) associated with antipsychotic drug treatment. For interpretation, a number larger than zero favours the column-defining treatment of a cell, i.e. this treatment leads to an increase in BMI. Values depicted are mean differences with associated 95% confidence intervals.

**aripiprazole**										
-0.28 (-0.50 to-0.07)	**asenapine**									
-1.04 (-2.50 to 0.41)	-0.76 (-2.22 to 0.69)	**clozapine**								
0.10 (-0.09 to 0.30)	0.39 (0.17 to 0.61)	1.15 (-0.30 to 2.60)	**lurasidone**							
0.91 (0.39 to 1.43)	1.19 (0.65 to 1.73)	1.96 (0.44 to 3.47)	0.81 (0.28 to 1.34)	**molindone**						
-1.02 (-1.26 to-0.78)	-0.74 (-1.01 to-0.48)	0.02 (-1.41 to 1.45)	-1.13 (-1.38 to-0.88)	-1.94 (-2.44 to-1.43)	**olanzapine**					
0.21 (0.08 to 0.34)	0.49 (0.33 to 0.66)	1.26 (-0.19 to 2.70)	0.11 (-0.03 to 0.25)	-0.70 (-1.21 to-0.19)	1.24 (1.03 to 1.44)	**placebo**				
-1.82 (-3.33 to-0.31)	-1.54 (-3.07 to-0.01)	-0.78 (-2.87 to 1.32)	-1.92 (-3.45 to-0.40)	-2.73 (-4.33 to-1.13)	-0.80 (-2.33 to 0.74)	-2.03 (-3.55 to-0.51)	**quetiapine**			
-0.39 (-0.56 to-0.22)	-0.11 (-0.34 to 0.12)	0.65 (-0.80 to 2.11)	-0.50 (-0.71 to-0.28)	-1.30 (-1.81 to-0.79)	0.63 (0.39 to 0.88)	-0.60 (-0.76 to-0.44)	1.43 (-0.09 to 2.95)	**risperidone/paliperidone**		
0.31 (0.02 to 0.60)	0.59 (0.29 to 0.90)	1.36 (-0.11 to 2.83)	0.21 (-0.09 to 0.50)	-0.60 (-1.17 to-0.03)	1.34 (1.01 to 1.66)	0.10 (-0.16 to 0.36)	2.13 (0.59 to 3.67)	0.70 (0.40 to 1.01)	ziprasidone	

eTable 7. League table for changes in triglycerides associated with antipsychotic drug treatment (mmol/L). For interpretation, a number larger than zero favours the column-defining treatment of a cell, i.e. this treatment leads to an increase in triglycerides. Values depicted are mean differences with associated 95% confidence intervals.

**aripiprazole**										
-0.01 (-0.14 to 0.12)	**asenapine**									
0.03 (-0.15 to 0.22)	0.04 (-0.15 to 0.24)	**blonanserin**								
-0.35 (-0.89 to 0.19)	-0.34 (-0.89 to 0.22)	-0.38 (-0.95 to 0.19)	**clozapine**							
0.20 (0.08 to 0.31)	0.21 (0.07 to 0.35)	0.17 (-0.02 to 0.36)	0.55 (0.00 to 1.10)	**lurasidone**						
0.13 (-0.08 to 0.35)	0.15 (-0.09 to 0.39)	0.10 (-0.17 to 0.38)	0.48 (-0.08 to 1.05)	-0.06 (-0.30 to 0.17)	**molindone**					
-0.12 (-0.27 to 0.03)	-0.11 (-0.29 to 0.08)	-0.15 (-0.38 to 0.07)	0.23 (-0.29 to 0.75)	-0.32 (-0.50 to-0.14)	-0.26 (-0.49 to-0.03)	**olanzapine**				
0.17 (0.09 to 0.24)	0.18 (0.07 to 0.29)	0.14 (-0.03 to 0.30)	0.52 (-0.02 to 1.06)	-0.03 (-0.12 to 0.06)	0.03 (-0.18 to 0.25)	0.29 (0.14 to 0.44)	**placebo**			
-0.04 (-0.09 to 0.02)	-0.02 (-0.16 to 0.11)	-0.07 (-0.25 to 0.12)	0.31 (-0.23 to 0.85)	-0.23 (-0.35 to-0.12)	-0.17 (-0.39 to 0.05)	0.08 (-0.07 to 0.24)	-0.20 (-0.28 to-0.13)	**quetiapine**		
-0.02 (-0.11 to 0.06)	-0.01 (-0.15 to 0.13)	-0.06 (-0.25 to 0.13)	0.33 (-0.21 to 0.86)	-0.22 (-0.35 to-0.10)	-0.16 (-0.36 to 0.04)	0.10 (-0.05 to 0.24)	-0.19 (-0.28 to-0.10)	0.01 (-0.09 to 0.11)	**risperidone/paliperidone**	
0.20 (0.06 to 0.34)	0.21 (0.05 to 0.37)	0.17 (-0.04 to 0.37)	0.55 (-0.01 to 1.10)	-0.00 (-0.15 to 0.15)	0.06 (-0.18 to 0.31)	0.32 (0.13 to 0.51)	0.03 (-0.09 to 0.15)	0.23 (0.09 to 0.37)	0.22 (0.07 to 0.37)	**ziprasidone**

eTable 8. League table for changes in prolactin associated with antipsychotic drug treatment (mmol/L). For interpretation, a number larger than zero favours the column-defining treatment of a cell, i.e. this treatment leads to an increase in weight. Values depicted are mean differences with associated 95% confidence intervals.

**aripiprazole**								
-3.33 (-15.51 to 8.86)	**asenapine**							
-12.10 (-31.17 to 6.96)	-8.78 (-29.98 to 12.42)	**blonanserin**						
-4.79 (-15.08 to 5.50)	-1.47 (-15.32 to 12.39)	7.31 (-12.86 to 27.48)	**lurasidone**					
-3.15 (-20.40 to 14.11)	0.18 (-19.63 to 19.99)	8.96 (-15.68 to 33.60)	1.65 (-17.05 to 20.35)	**molindone**				
-12.67 (-23.56 to -1.79)	-9.35 (-23.79 to 5.09)	-0.57 (-21.15 to 20.01)	-7.88 (-20.76 to 5.00)	-9.53 (-25.88 to 6.82)	**olanzapine**			
-2.83 (-8.42 to 2.75)	0.49 (-10.34 to 11.32)	9.27 (-8.96 to 27.49)	1.96 (-6.68 to 10.60)	0.31 (-16.27 to 16.90)	9.84 (0.29 to 19.39)	**placebo**		
-3.71 (-12.35 to 4.92)	-0.39 (-13.68 to 12.90)	8.39 (-11.40 to 28.18)	1.08 (-10.50 to 12.66)	-0.57 (-18.79 to 17.66)	8.96 (-3.27 to 21.19)	-0.88 (-8.58 to 6.83)	**quetiapine**	
-29.24 (-35.99 to-22.48)	-25.91 (-37.95 to-13.87)	-17.14 (-36.11 to 1.84)	-24.45 (-34.57 to-14.33)	-26.09 (-42.43 to -9.76)	-16.57 (-26.70 to -6.43)	-26.40 (-31.67 to-21.13)	-25.53 (-34.68 to-16.38)	**risperidone_paliperidone**

eTable 9. League table for changes in glucose associated with antipsychotic drug treatment (mmol/L). For interpretation, a number larger than zero favours the column-defining treatment of a cell, i.e. this treatment leads to an increase in glucose. Values depicted are mean differences with associated 95% confidence intervals.

**aripiprazole**										
-0.066 (-1.17 to 1.04)	**asenapine**									
0.19 (-1.28 to 1.66)	0.26 (-1.41 to 1.92)	**blonanserin**								
-0.61 (-2.25 to 1.01)	-0.55 (-2.41to 1.32)	-0.81 (-2.91 to 1.3)	**clozapine**							
0.092 (-1.015 to 1.2)	0.16 (-1.20 to 1.52)	-0.10 (-1.77 to 1.57)	0.71 (-1.16 to 2.58)	**lurasidone**						
-0.089 (-1.43 to 1.25)	-0.023 (-1.66 to 1.62	-0.28 (-2.18 to 1.62)	0.53 (-1.38 to 2.43)	-0.18 (-1.81 to 1.46)	**molindone**					
-0.05 (-0.83 to 0.72)	0.01(-1.191 to 1.21)	-0.25 (-1.79 to 1.29)	0.56 (-0.87 to 1.99)	-0.15 (-1.35 to 1.05)	0.03 (-1.2 to 1.29)	**olanzapine**				
0.11 (-0.44 to 0.66)	0.17 (-0.79 to 1.13)	-0.08 (-1.45 to 1.28)	0.72 (-0.88 to 2.32)	0.01 (-0.95to 0.98)	0.19 (-1.13 to 1.52)	0.16 (-0.56 to 0.88)	**placebo**			
-0.63 (-1.42 to 0.168)	-0.56 (-1.749 to 0.62)	-0.82 (-2.35to 0.71)	-0.01 (-1.74 to 1.72)	-0.72 (-1.90 to 0.46)	-0.54 (-2.01 to 0.94)	-0.58 (-1.55 to 0.40)	-0.73 (-1.43 to-0.04)	**quetiapine**		
-0.12 (-0.75 to 0.51)	-0.05 (-1.21 to 1.1)	-0.31 (-1.82 to 1.19)	0.49 (-1.12 to 2.12)	-0.21 (-1.37 to 0.94)	-0.03 (-1.29 to 1.22)	-0.06 (-0.82 to 0.69)	-0.23(-0.87 to 0.41)	0.51 (-0.39 to 1.41)	**risperidone/paliperidone**	
0.14 (-0.82 to 1.11)	0.21 (-1.03 to 1.46)	-0.05 (-1.62 to 1.53)	0.76 (-1.08 to 2.55)	0.05 (-1.19 to 1.30)	0.23 (-1.31 to 1.78)	0.2 (-0.88 to 1.28)	0.039 (-0.75 to 0.83)	0.77 (-0.28 to 1.83)	0.27 (-0.75 to 1.2)	ziprasidone

eTable 10. League table for changes in total cholesterol associated with antipsychotic drug treatment (mmol/L). For interpretation, a number larger than zero favours the column-defining treatment of a cell, i.e. this treatment leads to an increase in total cholesterol. Values depicted are mean differences with associated 95% confidence intervals

**aripiprazole**										
-0.18 (-0.47 to 0.11)	**asenapine**									
0.09 (-0.30 to 0.48)	0.27 (-0.15 to 0.70)	**blonanserin**								
-0.25 (-0.92 to 0.42)	-0.07 (-0.77 to 0.64)	-0.34 (-1.09 to 0.41)	**clozapine**							
0.03 (-0.26 to 0.32)	0.21 (-0.12 to 0.54)	-0.06 (-0.48 to 0.36)	0.28 (-0.43 to 0.98)	**lurasidone**						
-0.00 (-0.38 to 0.38)	0.18 (-0.26 to 0.62)	-0.09 (-0.60 to 0.42)	0.25 (-0.46 to 0.96)	-0.03 (-0.47 to 0.41)	**molindone**					
-0.28 (-0.53 to-0.03)	-0.10 (-0.43 to 0.24)	-0.37 (-0.80 to 0.06)	-0.03 (-0.65 to 0.59)	-0.31 (-0.65 to 0.03)	-0.28 (-0.62 to 0.07)	**olanzapine**				
0.05 (-0.12 to 0.23)	0.24 (0.00 to 0.47)	-0.04 (-0.39 to 0.31)	0.30 (-0.36 to 0.97)	0.02 (-0.21 to 0.26)	0.05 (-0.32 to 0.42)	0.33 (0.09 to 0.58)	**placebo**			
-0.30 (-0.53 to-0.07)	-0.11 (-0.41 to 0.18)	-0.39 (-0.78 to 0.01)	-0.05 (-0.73 to 0.64)	-0.33 (-0.62 to-0.03)	-0.30 (-0.70 to 0.11)	-0.02 (-0.31 to 0.28)	-0.35 (-0.53 to-0.17)	**quetiapine**		
0.02 (-0.17 to 0.21)	0.20 (-0.09 to 0.49)	-0.07 (-0.46 to 0.32)	0.27 (-0.39 to 0.93)	-0.01 (-0.30 to 0.28)	0.02 (-0.33 to 0.37)	0.30 (0.07 to 0.53)	-0.03 (-0.20 to 0.14)	0.32 (0.08 to 0.56)	**risperidone/paliperidone**	
0.05 (-0.21 to 0.32)	0.23 (-0.07 to 0.54)	-0.04 (-0.44 to 0.37)	0.30 (-0.39 to 0.99)	0.02 (-0.28 to 0.33)	0.05 (-0.37 to 0.47)	0.33 (0.02 to 0.65)	-0.00 (-0.20 to 0.20)	0.35 (0.08 to 0.62)	0.03 (-0.23 to 0.29)	**ziprasidone**

eTable 11. League table for changes in High Density Lipoprotein (HDL) cholesterol associated with antipsychotic drug treatment (mmol/L). For interpretation, a number larger than zero favours the column-defining treatment of a cell, i.e. this treatment leads to an increase in HDL cholesterol. Values depicted are mean differences with associated 95% confidence intervals.

**aripiprazole**						
-0.23 (-0.79 to 0.34)	**molindone**					
-0.09 (-0.46 to 0.29)	0.14 (-0.35 to 0.63)	**olanzapine**				
0.20 (-0.08 to 0.48)	0.43 (-0.11 to 0.97)	0.29 (-0.05 to 0.64)	**placebo**			
0.15 (-0.17 to 0.48)	0.38 (-0.21 to 0.96)	0.24 (-0.17 to 0.65)	-0.05 (-0.30 to 0.19)	**quetiapine**		
-0.27 (-0.62 to 0.08)	-0.05 (-0.54 to 0.44)	-0.18 (-0.51 to 0.14)	-0.48 (-0.78 to-0.18)	-0.43 (-0.80 to-0.05)	**risperidone/paliperidone**	
0.15 (-0.27 to 0.56)	0.37 (-0.25 to 0.99)	0.23 (-0.22 to 0.69)	-0.06 (-0.36 to 0.25)	-0.01 (-0.40 to 0.38)	0.42 (-0.01 to 0.85)	**ziprasidone**

eTable 12. League table for changes in Low Density Lipoprotein (LDL) cholesterol associated with antipsychotic drug treatment (mmol/L). For interpretation, a number larger than zero favours the column-defining treatment of a cell, i.e. this treatment leads to an increase in LDL cholesterol. Values depicted are mean differences with associated 95% confidence intervals.

**aripiprazole**							
0.03 (-0.30 to 0.36)	**lurasidone**						
-0.01 (-0.33 to 0.30)	-0.04 (-0.46 to 0.37)	**molindone**					
-0.22 (-0.43 to 0.00)	-0.25 (-0.59 to 0.10)	-0.20 (-0.48 to 0.07)	**olanzapine**				
-0.05 (-0.25 to 0.16)	-0.08 (-0.34 to 0.18)	-0.04 (-0.36 to 0.29)	0.17 (-0.06 to 0.40)	**placebo**			
-0.08 (-0.31 to 0.15)	-0.11 (-0.43 to 0.20)	-0.07 (-0.42 to 0.28)	0.14 (-0.13 to 0.40)	-0.03 (-0.21 to 0.14)	**quetiapine**		
0.07 (-0.10 to 0.24)	0.04 (-0.29 to 0.36)	0.08 (-0.20 to 0.36)	0.29 (0.09 to 0.48)	0.12 (-0.07 to 0.31)	0.15 (-0.09 to 0.38)	**risperidone/paliperidone**	
-0.00 (-0.26 to 0.26)	-0.03 (-0.34 to 0.27)	0.01 (-0.35 to 0.37)	0.22 (-0.06 to 0.49)	0.05 (-0.11 to 0.20)	0.08 (-0.15 to 0.31)	-0.07 (-0.32 to 0.18)	**ziprasidone**

eTable 13. League table for changes heart rate associated with antipsychotic drug treatment (bpm). For interpretation, a number larger than zero favours the column-defining treatment of a cell, i.e. this treatment leads to an increase in heart rate. Values depicted are mean differences with associated 95% confidence intervals.

**aripiprazole**						
-3.62 (-15.82 to 8.58)	**haloperidol**					
2.69 (-9.47 to14.85)	6.31 (-7.59 to20.22)	**olanzapine**				
3.35 (-5.26 to11.96)	6.97 (-3.96 to17.90)	0.66 (-7.93 to 9.25)	**placebo**			
-9.07 (-16.66 to-1.49)	-5.45 (-18.34 to 7.43)	-11.76 (-23.91 to 0.39)	-12.42 (-21.01 to-3.83)	**quetiapine**		
-1.92 (-9.75 to 5.91)	1.70 (-7.66 to11.06)	-4.61 (-14.90 to 5.68)	-5.27 (-10.93 to 0.39)	7.15 (-1.71 to16.01)	**risperidone/paliperidone**	
3.55 (-8.14 to15.24)	7.17 (-6.33 to20.67)	0.86 (-10.82 to12.54)	0.20 (-7.71 to 8.11)	12.62 (0.94 to24.30)	5.47 (-4.26 to15.20)	**ziprasidone**

eTable 14. League table for changes in QTc associated with antipsychotic drug treatment (SMD). For interpretation, a number larger than zero favours the column-defining treatment of a cell, i.e. this treatment leads to an increase in QTc interval. Values depicted are standardised mean differences with associated 95% confidence intervals.

**aripiprazole**							
0.22 (-0.34 to 0.77)	**molindone**						
-0.10 (-0.48 to 0.29)	-0.31 (-0.76 to 0.13)	**olanzapine**					
0.52 (-0.39 to 1.42)	0.30 (-0.60 to 1.20)	0.61 (-0.25 to 1.48)	**pimozide**				
-0.09 (-0.36 to 0.18)	-0.31 (-0.79 to 0.17)	0.00 (-0.27 to 0.28)	-0.61 (-1.47 to 0.26)	**placebo**			
-0.35 (-0.72 to 0.02)	-0.57 (-1.21 to 0.08)	-0.25 (-0.76 to 0.26)	-0.86 (-1.83 to 0.10)	-0.26 (-0.69 to 0.17)	**quetiapine**		
0.15 (-0.29 to 0.60)	-0.06 (-0.50 to 0.37)	0.25 (-0.11 to 0.61)	-0.36 (-1.15 to 0.42)	0.24 (-0.11 to 0.60)	0.50 (-0.05 to 1.06)	**risperidone/paliperidone**	
-0.39 (-0.83 to 0.05)	-0.61 (-1.20 to-0.01)	-0.29 (-0.74 to 0.16)	-0.90 (-1.84 to 0.03)	-0.30 (-0.65 to 0.05)	-0.04 (-0.59 to 0.51)	-0.54 (-1.04 to-0.04)	ziprasidone

eTable 15. League table for changes in systolic blood pressure associated with antipsychotic drug treatment (mmHg). For interpretation, a number larger than zero favours the column-defining treatment of a cell, i.e. this treatment leads to an increase in blood pressure. Values depicted are mean differences with associated 95% confidence intervals.

**aripiprazole**							
9.13 (-5.39 to 23.65)	**clozapine**						
4.26 (-1.97 to10.49)	-4.87 (-17.99 to 8.25)	**haloperidol**					
-2.03 (-7.25 to 3.18)	-11.16 (-26.08 to 3.75)	-6.29 (-13.39 to 0.81)	**olanzapine**				
3.85 (0.07 to 7.63)	-5.28 (-19.76 to 9.20)	-0.41 (-6.53 to 5.71)	5.88 (2.29 to 9.47)	**placebo**			
-6.19 (-10.21 to-2.17)	-15.32 (-30.20 to-0.44)	-10.45 (-17.47 to-3.43)	-4.16 (-9.89 to 1.58)	-10.04 (-14.51 to-5.56)	**quetiapine**		
-0.24 (-4.26 to 3.78)	-9.37 (-23.33 to 4.59)	-4.50 (-9.26 to 0.26)	1.79 (-3.47 to 7.05)	-4.09 (-7.93 to-0.24)	5.95 (0.79 to11.10)	**risperidone/paliperidone**	
7.25 (2.51 to11.99)	-1.88 (-16.64 to12.87)	2.99 (-3.76 to 9.74)	9.28 (4.69 to13.87)	3.40 (0.55 to 6.25)	13.44 (8.13 to18.74)	7.49 (2.70 to12.28)	ziprasidone

eAppendix 6. Full results for HDL-cholesterol and LDL-cholesterol

For HDL-cholesterol, 15 studies compared 7 different antipsychotics with placebo (1528 and 686 patients, respectively) (Figure 3 and appendix p39). Compared with placebo, there was evidence of an increase in HDL cholesterol (MD relative to placebo (mmol/L), 95%CI) with risperidone/paliperidone (0.48, 0.18 to 0.78). There was evidence of an effect of aripiprazole (0.20, -0.08 to 0.48), olanzapine (0.29, -0.05, 0.64), and molindone (0.43, -0.11 to 0.97), but with increased uncertainty. We found no strong evidence of HDL cholesterol change with quetiapine or ziprasidone. Ranking based on degree of HDL cholesterol increase identified risperidone/paliperidone as the best and quetiapine the worst (appendix p44). τ was 0.27mmol/L, considered large in the context of the observed antipsychotic-associated changes. The global test for inconsistency found evidence of inconsistency ($p<0.0001$), as did the back-calculation method (appendix p56). Certainty of evidence was very low in 21/21 (100%) of comparisons (appendix p80).

For LDL-cholesterol, 14 studies compared 8 different antipsychotics with placebo (1589 and 672 patients, respectively) (Figure 3 and appendix p39). Compared with placebo, there was some evidence of an increase in LDL cholesterol (MD relative to placebo (mmol/L), 95%CI) with olanzapine (0.17, -0.06 to 0.40) but with a degree of uncertainty. There was also evidence of a reduction in LDL cholesterol with risperidone/paliperidone (-0.12, -0.31 to 0.07) but again this result was uncertain. We found no strong evidence of change in LDL cholesterol with lurasidone, aripiprazole, ziprasidone, molindone, or quetiapine. Ranking based on degree of LDL cholesterol increase identified risperidone/paliperidone as the best and olanzapine the worst (appendix p45). τ was 0.12mmol/L, considered large in the context of the observed antipsychotic-associated changes. The global test for inconsistency found evidence of inconsistency ($p=0.0016$), as did the back-calculation method (appendix p57). Certainty of evidence was very low in 23/28 (82%) of comparisons (appendix p81).

eFigure 7. Prolactin elevation: SMD results

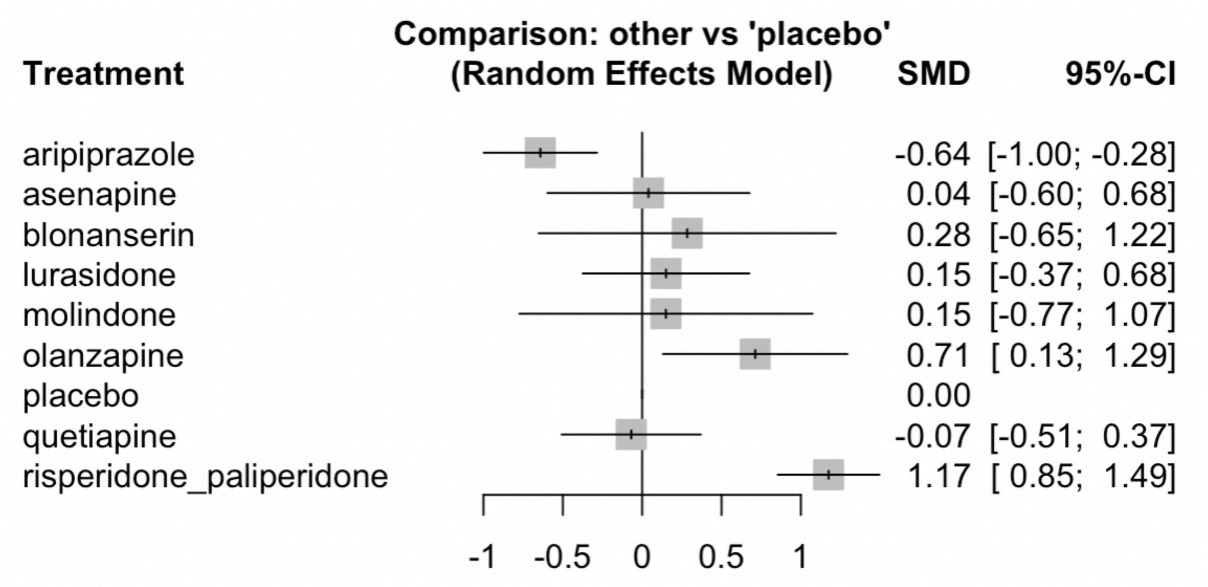

Tau = 0.45; Q=154.16 (p<0.0001)

eTable 16. P-score ranking: weight

	P-score (common)	P-score (random)
molindone	0.9679	0.9623
ziprasidone	0.8550	0.8260
placebo	0.7528	0.7526
blonanserin	0.7454	0.7283
lurasidone	0.6375	0.6490
pimozide	0.5329	0.5315
aripiprazole	0.5370	0.5166
asenapine	0.4131	0.4425
risperidone_paliperidone	0.3612	0.3587
quetiapine	0.3204	0.3563
olanzapine	0.1501	0.1498
clozapine	0.1278	0.1281
haloperidol	0.0989	0.0983

eTable 17. P-score ranking: BMI

	P-score (common)	P-score (random)
molindone	0.9965	0.9965
ziprasidone	0.8510	0.8510
placebo	0.7898	0.7898
lurasidone	0.6602	0.6602
aripiprazole	0.5633	0.5633
asenapine	0.4053	0.4053
risperidone/paliperidone	0.3288	0.3288
clozapine	0.2022	0.2022
olanzapine	0.1507	0.1507
quetiapine	0.0523	0.0523

eTable 18. P-score ranking: triglycerides

	P-score (common)	P-score (random)
clozapine	0.9164	0.9117
olanzapine	0.8798	0.8664
quetiapine	0.6663	0.7092
risperidone/paliperidone	0.6849	0.6555
asenapine	0.6242	0.6170
aripiprazole	0.5534	0.5495
blonanserin	0.4936	0.5023
molindone	0.2762	0.2644
placebo	0.1931	0.1905
ziprasidone	0.1126	0.1210
lurasidone	0.0994	0.1124

eTable 19. P-score ranking: prolactin

	P-score (common)	P-score (random)
aripiprazole	0.9340	0.8356
placebo	0.7028	0.6612
molindone	0.8700	0.6350
asenapine	0.5352	0.6309
quetiapine	0.5781	0.6073
lurasidone	0.4674	0.5540
blonanserin	0.2208	0.3297
olanzapine	0.1785	0.2413
risperidone_paliperidone	0.0131	0.0050

eTable 19. P-score ranking: glucose

	P-score (common)	P-score (random)
placebo	0.7777	0.6543
ziprasidone	0.6943	0.6471
blonanserin	0.9111	0.6363
lurasidone	0.6689	0.6037
aripiprazole	0.5768	0.5578
olanzapine	0.5816	0.5166
asenapine	0.1829	0.5059
molindone	0.3421	0.4978
risperidone/paliperidone	0.1938	0.4567
clozapine	0.0192	0.2654
quetiapine	0.5518	0.1583

eTable 20. P-score ranking: total cholesterol

	P-score (common)	P-score (random)
blonanserin	0.8297	0.7350
placebo	0.7889	0.7266
ziprasidone	0.7483	0.7037
lurasidone	0.7290	0.6534
risperidone/paliperidone	0.5273	0.6423
molindone	0.6112	0.5966
aripiprazole	0.5121	0.5929
clozapine	0.2940	0.3001
asenapine	0.2294	0.2765
olanzapine	0.1195	0.1468
quetiapine	0.1107	0.1263

eTable 21. P-score ranking: HDL cholesterol

	P-score (common)	P-score (random)
risperidone/paliperidone	0.9139	0.8897
molindone	0.9076	0.7725
olanzapine	0.6776	0.6293
aripiprazole	0.5008	0.5166
ziprasidone	0.3192	0.2850

quetiapine	0.1711	0.2598
placebo	0.0097	0.1471

eTable 22. P-score ranking: LDL cholesterol

	P-score (common)	P-score (random)
risperidone/paliperidone	0.8362	0.7973
lurasidone	0.7484	0.6516
ziprasidone	0.5996	0.5906
aripiprazole	0.5936	0.5831
molindone	0.6006	0.5427
placebo	0.3846	0.4249
quetiapine	0.2031	0.3438
olanzapine	0.0339	0.0661

eTable 23. P-score ranking: heart rate

	P-score (common)	P-score (random)
placebo	0.8225	0.7792
ziprasidone	0.8283	0.7500
olanzapine	0.7378	0.6908
aripiprazole	0.4906	0.5375
risperidone/paliperidone	0.3444	0.3761
haloperidol	0.2653	0.3132
quetiapine	0.0111	0.0530

eTable 24. P-score ranking: QTc interval

	P-score (common)	P-score (random)
pimozide	0.8904	0.8846
molindone	0.7776	0.7711
risperidone/paliperidone	0.7350	0.7265
aripiprazole	0.5684	0.5672
placebo	0.4106	0.4104
olanzapine	0.3939	0.3975
quetiapine	0.1305	0.1419
ziprasidone	0.0935	0.1008

eTable 25. P-score ranking: systolic blood pressure

	P-score (common)	P-score (random)
ziprasidone	0.8852	0.8852
clozapine	0.8331	0.8331
haloperidol	0.6877	0.6877
placebo	0.6646	0.6646
aripiprazole	0.3638	0.3638
risperidone/paliperidone	0.3336	0.3336
olanzapine	0.2157	0.2157
quetiapine	0.0164	0.0164

eTable 26. ‘Hot spots’ of inconsistency: Weight

Separate indirect from direct evidence (SIDE) using back-calculation method

Common effects model:

comparison	k	prop	nma	direct	indir.	Diff	z	p-value
asenapine:aripiprazole	0	0	0.4010	.	0.4010	.	.	.
blonanserin:aripiprazole	0	0	-0.6967	.	-0.6967	.	.	.
clozapine:aripiprazole	0	0	3.7615	.	3.7615	.	.	.
haloperidol:aripiprazole	0	0	4.6506	.	4.6506	.	.	.
lurasidone:aripiprazole	0	0	-0.3746	.	-0.3746	.	.	.
molindone:aripiprazole	0	0	-2.8711	.	-2.8711	.	.	.
olanzapine:aripiprazole	1	0.19	2.8350	2.8500	2.8315	0.0185	0.03	0.9791
pimozide:aripiprazole	0	0	-0.1721	.	-0.1721	.	.	.
placebo:aripiprazole	6	0.63	-0.7144	-0.7166	-0.7107	-0.0060	-0.02	0.9839
quetiapine:aripiprazole	1	< 0.01	0.6148	2.9100	0.6115	2.2985	0.44	0.6609
risperidone_paliperidone:aripiprazole	4	0.53	0.5279	0.5130	0.5447	-0.0317	-0.11	0.9161
ziprasidone:aripiprazole	0	0	-0.9944	.	-0.9944	.	.	.
asenapine:blonanserin	0	0	1.0977	.	1.0977	.	.	.
asenapine:clozapine	0	0	-3.3605	.	-3.3605	.	.	.
asenapine:haloperidol	0	0	-4.2496	.	-4.2496	.	.	.
asenapine:lurasidone	0	0	0.7756	.	0.7756	.	.	.
asenapine:molindone	0	0	3.2721	.	3.2721	.	.	.
asenapine:olanzapine	0	0	-2.4340	.	-2.4340	.	.	.
asenapine:pimozide	0	0	0.5731	.	0.5731	.	.	.
asenapine:placebo	1	1.00	1.1154	1.1154	.	.	.	.
asenapine:quetiapine	0	0	-0.2138	.	-0.2138	.	.	.
asenapine:risperidone_paliperidone	0	0	-0.1269	.	-0.1269	.	.	.
asenapine:ziprasidone	0	0	1.3954	.	1.3954	.	.	.
blonanserin:clozapine	0	0	-4.4581	.	-4.4581	.	.	.
blonanserin:haloperidol	0	0	-5.3473	.	-5.3473	.	.	.
blonanserin:lurasidone	0	0	-0.3221	.	-0.3221	.	.	.
blonanserin:molindone	0	0	2.1744	.	2.1744	.	.	.
blonanserin:olanzapine	0	0	-3.5317	.	-3.5317	.	.	.
blonanserin:pimozide	0	0	-0.5245	.	-0.5245	.	.	.
blonanserin:placebo	1	1.00	0.0178	0.0178	.	.	.	.
blonanserin:quetiapine	0	0	-1.3115	.	-1.3115	.	.	.
blonanserin:risperidone_paliperidone	0	0	-1.2245	.	-1.2245	.	.	.
blonanserin:ziprasidone	0	0	0.2978	.	0.2978	.	.	.
clozapine:haloperidol	1	0.87	-0.8891	-0.0400	-6.6049	6.5649	1.06	0.2884
clozapine:lurasidone	0	0	4.1360	.	4.1360	.	.	.
clozapine:molindone	0	0	6.6325	.	6.6325	.	.	.
clozapine:olanzapine	1	0.89	0.9264	0.2000	6.7649	-6.5649	-1.06	0.2884
clozapine:pimozide	0	0	3.9336	.	3.9336	.	.	.
clozapine:placebo	0	0	4.4759	.	4.4759	.	.	.
clozapine:quetiapine	0	0	3.1467	.	3.1467	.	.	.
clozapine:risperidone_paliperidone	0	0	3.2336	.	3.2336	.	.	.
clozapine:ziprasidone	0	0	4.7559	.	4.7559	.	.	.
haloperidol:lurasidone	0	0	5.0252	.	5.0252	.	.	.
haloperidol:molindone	0	0	7.5217	.	7.5217	.	.	.
haloperidol:olanzapine	0	0	1.8156	.	1.8156	.	.	.
haloperidol:pimozide	0	0	4.8227	.	4.8227	.	.	.
haloperidol:placebo	0	0	5.3650	.	5.3650	.	.	.
haloperidol:quetiapine	0	0	4.0358	.	4.0358	.	.	.
haloperidol:risperidone_paliperidone	1	0.24	4.1227	9.1000	2.5351	6.5649	1.06	0.2884
haloperidol:ziprasidone	0	0	5.6450	.	5.6450	.	.	.
lurasidone:molindone	0	0	2.4965	.	2.4965	.	.	.
lurasidone:olanzapine	0	0	-3.2096	.	-3.2096	.	.	.
lurasidone:pimozide	0	0	-0.2024	.	-0.2024	.	.	.
lurasidone:placebo	3	1.00	0.3398	0.3398	.	.	.	.
lurasidone:quetiapine	0	0	-0.9894	.	-0.9894	.	.	.
lurasidone:risperidone_paliperidone	0	0	-0.9024	.	-0.9024	.	.	.
lurasidone:ziprasidone	0	0	0.6198	.	0.6198	.	.	.
molindone:olanzapine	1	0.72	-5.7061	-5.8000	-5.4670	-0.3330	-0.23	0.8165
molindone:pimozide	0	0	-2.6989	.	-2.6989	.	.	.
molindone:placebo	0	0	-2.1567	.	-2.1567	.	.	.
molindone:quetiapine	0	0	-3.4859	.	-3.4859	.	.	.
molindone:risperidone_paliperidone	1	0.70	-3.3989	-3.3000	-3.6268	0.3268	0.23	0.8165
molindone:ziprasidone	0	0	-1.8767	.	-1.8767	.	.	.
olanzapine:pimozide	0	0	3.0072	.	3.0072	.	.	.
olanzapine:placebo	2	0.74	3.5495	3.4560	3.8113	-0.3554	-0.62	0.5320
olanzapine:quetiapine	0	0	2.2202	.	2.2202	.	.	.
olanzapine:risperidone_paliperidone	2	0.27	2.3072	2.6507	2.1819	0.4687	0.78	0.4334
olanzapine:ziprasidone	0	0	3.8294	.	3.8294	.	.	.
pimozide:placebo	0	0	0.5423	.	0.5423	.	.	.
pimozide:quetiapine	0	0	-0.7869	.	-0.7869	.	.	.

```
       pimozide:risperidone_paliperidone 1   1.00 -0.7000 -0.7000       .       .     .       .
                    pimozide:ziprasidone 0      0  0.8223       .  0.8223       .     .       .
                      placebo:quetiapine 4   1.00 -1.3292 -1.3277 -3.6261  2.2985  0.44  0.6609
        placebo:risperidone_paliperidone 9   0.80 -1.2423 -1.2606 -1.1704 -0.0901 -0.32  0.7512
                     placebo:ziprasidone 3   1.00  0.2800  0.2800       .       .     .       .
     quetiapine:risperidone_paliperidone 0      0  0.0869       .  0.0869       .     .       .
                  quetiapine:ziprasidone 0      0  1.6092       .  1.6092       .     .       .
    risperidone_paliperidone:ziprasidone 0      0  1.5223       .  1.5223       .     .       .

Random effects model:

                              comparison k   prop     nma  direct  indir.    Diff     z p-value
                   asenapine:aripiprazole 0      0  0.2639       .  0.2639       .     .       .
                 blonanserin:aripiprazole 0      0 -0.8337       . -0.8337       .     .       .
                   clozapine:aripiprazole 0      0  3.8416       .  3.8416       .     .       .
                 haloperidol:aripiprazole 0      0  4.7500       .  4.7500       .     .       .
                  lurasidone:aripiprazole 0      0 -0.5080       . -0.5080       .     .       .
                   molindone:aripiprazole 0      0 -2.8503       . -2.8503       .     .       .
                  olanzapine:aripiprazole 1   0.30  2.8919  2.8500  2.9097 -0.0597 -0.06  0.9519
                    pimozide:aripiprazole 0      0 -0.1903       . -0.1903       .     .       .
                     placebo:aripiprazole 6   0.59 -0.8515 -0.8764 -0.8163 -0.0601 -0.11  0.9142
                  quetiapine:aripiprazole 1 < 0.01  0.5451  2.9100  0.5305  2.3795  0.45  0.6522
    risperidone_paliperidone:aripiprazole 4   0.56  0.5097  0.5048  0.5157 -0.0109 -0.02  0.9847
                 ziprasidone:aripiprazole 0      0 -1.1509       . -1.1509       .     .       .
                    asenapine:blonanserin 0      0  1.0977       .  1.0977       .     .       .
                      asenapine:clozapine 0      0 -3.5777       . -3.5777       .     .       .
                    asenapine:haloperidol 0      0 -4.4860       . -4.4860       .     .       .
                     asenapine:lurasidone 0      0  0.7719       .  0.7719       .     .       .
                      asenapine:molindone 0      0  3.1142       .  3.1142       .     .       .
                     asenapine:olanzapine 0      0 -2.6280       . -2.6280       .     .       .
                       asenapine:pimozide 0      0  0.4542       .  0.4542       .     .       .
                        asenapine:placebo 1   1.00  1.1154  1.1154       .       .     .       .
                     asenapine:quetiapine 0      0 -0.2812       . -0.2812       .     .       .
       asenapine:risperidone_paliperidone 0      0 -0.2458       . -0.2458       .     .       .
                    asenapine:ziprasidone 0      0  1.4148       .  1.4148       .     .       .
                    blonanserin:clozapine 0      0 -4.6754       . -4.6754       .     .       .
                  blonanserin:haloperidol 0      0 -5.5837       . -5.5837       .     .       .
                   blonanserin:lurasidone 0      0 -0.3258       . -0.3258       .     .       .
                    blonanserin:molindone 0      0  2.0166       .  2.0166       .     .       .
                   blonanserin:olanzapine 0      0 -3.7257       . -3.7257       .     .       .
                     blonanserin:pimozide 0      0 -0.6434       . -0.6434       .     .       .
                      blonanserin:placebo 1   1.00  0.0178  0.0178       .       .     .       .
                   blonanserin:quetiapine 0      0 -1.3788       . -1.3788       .     .       .
     blonanserin:risperidone_paliperidone 0      0 -1.3434       . -1.3434       .     .       .
                  blonanserin:ziprasidone 0      0  0.3172       .  0.3172       .     .       .
                    clozapine:haloperidol 1   0.87 -0.9083 -0.0400 -6.5470  6.5070  1.04  0.2986
                     clozapine:lurasidone 0      0  4.3496       .  4.3496       .     .       .
                      clozapine:molindone 0      0  6.6920       .  6.6920       .     .       .
                     clozapine:olanzapine 1   0.88  0.9497  0.2000  6.7070 -6.5070 -1.04  0.2986
                       clozapine:pimozide 0      0  4.0320       .  4.0320       .     .       .
                        clozapine:placebo 0      0  4.6931       .  4.6931       .     .       .
                     clozapine:quetiapine 0      0  3.2966       .  3.2966       .     .       .
       clozapine:risperidone_paliperidone 0      0  3.3320       .  3.3320       .     .       .
                    clozapine:ziprasidone 0      0  4.9925       .  4.9925       .     .       .
                   haloperidol:lurasidone 0      0  5.2579       .  5.2579       .     .       .
                    haloperidol:molindone 0      0  7.6003       .  7.6003       .     .       .
                   haloperidol:olanzapine 0      0  1.8580       .  1.8580       .     .       .
                     haloperidol:pimozide 0      0  4.9403       .  4.9403       .     .       .
                      haloperidol:placebo 0      0  5.6015       .  5.6015       .     .       .
                   haloperidol:quetiapine 0      0  4.2049       .  4.2049       .     .       .
     haloperidol:risperidone_paliperidone 1   0.25  4.2403  9.1000  2.5930  6.5070  1.04  0.2986
                  haloperidol:ziprasidone 0      0  5.9009       .  5.9009       .     .       .
                     lurasidone:molindone 0      0  2.3423       .  2.3423       .     .       .
                    lurasidone:olanzapine 0      0 -3.3999       . -3.3999       .     .       .
                      lurasidone:pimozide 0      0 -0.3177       . -0.3177       .     .       .
                       lurasidone:placebo 3   1.00  0.3435  0.3435       .       .     .       .
                    lurasidone:quetiapine 0      0 -1.0531       . -1.0531       .     .       .
      lurasidone:risperidone_paliperidone 0      0 -1.0177       . -1.0177       .     .       .
                   lurasidone:ziprasidone 0      0  0.6429       .  0.6429       .     .       .
                     molindone:olanzapine 1   0.76 -5.7422 -5.8000 -5.5612 -0.2388 -0.13  0.8996
                       molindone:pimozide 0      0 -2.6600       . -2.6600       .     .       .
                        molindone:placebo 0      0 -1.9988       . -1.9988       .     .       .
                     molindone:quetiapine 0      0 -3.3954       . -3.3954       .     .       .
       molindone:risperidone_paliperidone 1   0.74 -3.3600 -3.3000 -3.5352  0.2352  0.13  0.8996
                    molindone:ziprasidone 0      0 -1.6994       . -1.6994       .     .       .
                      olanzapine:pimozide 0      0  3.0823       .  3.0823       .     .       .
                       olanzapine:placebo 2   0.59  3.7434  3.5869  3.9646 -0.3777 -0.46  0.6448
```

olanzapine:quetiapine	0	0	2.3468	.	2.3468	.	.	.
olanzapine:risperidone_paliperidone	2	0.42	2.3823	2.6389	2.1935	0.4454	0.53	0.5993
olanzapine:ziprasidone	0	0	4.0428	.	4.0428	.	.	.
pimozide:placebo	0	0	0.6612	.	0.6612	.	.	.
pimozide:quetiapine	0	0	-0.7354	.	-0.7354	.	.	.
pimozide:risperidone_paliperidone	1	1.00	-0.7000	-0.7000	.	.	.	.
pimozide:ziprasidone	0	0	0.9606	.	0.9606	.	.	.
placebo:quetiapine	4	1.00	-1.3966	-1.3884	-3.7680	2.3795	0.45	0.6522
placebo:risperidone_paliperidone	9	0.78	-1.3612	-1.3875	-1.2663	-0.1211	-0.23	0.8144
placebo:ziprasidone	3	1.00	0.2994	0.2994	.	.	.	.
quetiapine:risperidone_paliperidone	0	0	0.0354	.	0.0354	.	.	.
quetiapine:ziprasidone	0	0	1.6960	.	1.6960	.	.	.
risperidone_paliperidone:ziprasidone	0	0	1.6606	.	1.6606	.	.	.

Legend:
comparison - Treatment comparison
k - Number of studies providing direct evidence
prop - Direct evidence proportion
nma - Estimated treatment effect (MD) in network meta-analysis
direct - Estimated treatment effect (MD) derived from direct evidence
indir. - Estimated treatment effect (MD) derived from indirect evidence
Diff - Difference between direct and indirect treatment estimates
z - z-value of test for disagreement (direct versus indirect)
p-value - p-value of test for disagreement (direct versus indirect)

eTable 27. 'Hot spots' of inconsistency: BMI

Separate indirect from direct evidence (SIDE) using back-calculation method

Common effects model:

comparison	k	prop	nma	direct	indir.	Diff	z	p-value
asenapine:aripiprazole	0	0	0.2818	.	0.2818	.	.	.
clozapine:aripiprazole	0	0	1.0448	.	1.0448	.	.	.
lurasidone:aripiprazole	0	0	-0.1047	.	-0.1047	.	.	.
molindone:aripiprazole	0	0	-0.9118	.	-0.9118	.	.	.
olanzapine:aripiprazole	0	0	1.0242	.	1.0242	.	.	.
placebo:aripiprazole	6	0.83	-0.2123	-0.2506	-0.0316	-0.2190	-1.25	0.2129
quetiapine:aripiprazole	1	1.00	1.8200	1.8200	.	.	.	.
risperidone/paliperidone:aripiprazole	1	0.50	0.3904	0.5000	0.2810	0.2190	1.25	0.2129
ziprasidone:aripiprazole	0	0	-0.3123	.	-0.3123	.	.	.
asenapine:clozapine	0	0	-0.7630	.	-0.7630	.	.	.
asenapine:lurasidone	0	0	0.3865	.	0.3865	.	.	.
asenapine:molindone	0	0	1.1936	.	1.1936	.	.	.
asenapine:olanzapine	0	0	-0.7424	.	-0.7424	.	.	.
asenapine:placebo	1	1.00	0.4941	0.4941	.	.	.	.
asenapine:quetiapine	0	0	-1.5382	.	-1.5382	.	.	.
asenapine:risperidone/paliperidone	0	0	-0.1085	.	-0.1085	.	.	.
asenapine:ziprasidone	0	0	0.5941	.	0.5941	.	.	.
clozapine:lurasidone	0	0	1.1495	.	1.1495	.	.	.
clozapine:molindone	0	0	1.9565	.	1.9565	.	.	.
clozapine:olanzapine	2	1.00	0.0206	0.0206	.	.	.	.
clozapine:placebo	0	0	1.2571	.	1.2571	.	.	.
clozapine:quetiapine	0	0	-0.7752	.	-0.7752	.	.	.
clozapine:risperidone/paliperidone	0	0	0.6544	.	0.6544	.	.	.
clozapine:ziprasidone	0	0	1.3571	.	1.3571	.	.	.
lurasidone:molindone	0	0	0.8071	.	0.8071	.	.	.
lurasidone:olanzapine	0	0	-1.1289	.	-1.1289	.	.	.
lurasidone:placebo	2	1.00	0.1076	0.1076	.	.	.	.
lurasidone:quetiapine	0	0	-1.9247	.	-1.9247	.	.	.
lurasidone:risperidone/paliperidone	0	0	-0.4950	.	-0.4950	.	.	.
lurasidone:ziprasidone	0	0	0.2076	.	0.2076	.	.	.
molindone:olanzapine	1	0.82	-1.9360	-2.0500	-1.4217	-0.6283	-0.94	0.3465
molindone:placebo	0	0	-0.6995	.	-0.6995	.	.	.
molindone:quetiapine	0	0	-2.7318	.	-2.7318	.	.	.
molindone:risperidone/paliperidone	1	0.72	-1.3021	-1.1500	-1.7011	0.5511	0.94	0.3465
molindone:ziprasidone	0	0	-0.5995	.	-0.5995	.	.	.
olanzapine:placebo	2	0.89	1.2365	1.2029	1.5227	-0.3198	-0.94	0.3465
olanzapine:quetiapine	0	0	-0.7958	.	-0.7958	.	.	.
olanzapine:risperidone/paliperidone	1	0.17	0.6338	0.9000	0.5802	0.3198	0.94	0.3465
olanzapine:ziprasidone	0	0	1.3365	.	1.3365	.	.	.

placebo:quetiapine	0	0	-2.0323	.	-2.0323	.	.	.
placebo:risperidone/paliperidone	3	0.61	-0.6027	-0.5549	-0.6781	0.1232	0.73	0.4660
placebo:ziprasidone	1	1.00	0.1000	0.1000	.	.	.	.
quetiapine:risperidone/paliperidone	0	0	1.4296	.	1.4296	.	.	.
quetiapine:ziprasidone	0	0	2.1323	.	2.1323	.	.	.
risperidone/paliperidone:ziprasidone	0	0	0.7027	.	0.7027	.	.	.

Random effects model:

comparison	k	prop	nma	direct	indir.	Diff	z	p-value
asenapine:aripiprazole	0	0	0.2818	.	0.2818	.	.	.
clozapine:aripiprazole	0	0	1.0448	.	1.0448	.	.	.
lurasidone:aripiprazole	0	0	-0.1047	.	-0.1047	.	.	.
molindone:aripiprazole	0	0	-0.9118	.	-0.9118	.	.	.
olanzapine:aripiprazole	0	0	1.0242	.	1.0242	.	.	.
placebo:aripiprazole	6	0.83	-0.2123	-0.2506	-0.0316	-0.2190	-1.25	0.2129
quetiapine:aripiprazole	1	1.00	1.8200	1.8200	.	.	.	.
risperidone/paliperidone:aripiprazole	1	0.50	0.3904	0.5000	0.2810	0.2190	1.25	0.2129
ziprasidone:aripiprazole	0	0	-0.3123	.	-0.3123	.	.	.
asenapine:clozapine	0	0	-0.7630	.	-0.7630	.	.	.
asenapine:lurasidone	0	0	0.3865	.	0.3865	.	.	.
asenapine:molindone	0	0	1.1936	.	1.1936	.	.	.
asenapine:olanzapine	0	0	-0.7424	.	-0.7424	.	.	.
asenapine:placebo	1	1.00	0.4941	0.4941	.	.	.	.
asenapine:quetiapine	0	0	-1.5382	.	-1.5382	.	.	.
asenapine:risperidone/paliperidone	0	0	-0.1085	.	-0.1085	.	.	.
asenapine:ziprasidone	0	0	0.5941	.	0.5941	.	.	.
clozapine:lurasidone	0	0	1.1495	.	1.1495	.	.	.
clozapine:molindone	0	0	1.9565	.	1.9565	.	.	.
clozapine:olanzapine	2	1.00	0.0206	0.0206	.	.	.	.
clozapine:placebo	0	0	1.2571	.	1.2571	.	.	.
clozapine:quetiapine	0	0	-0.7752	.	-0.7752	.	.	.
clozapine:risperidone/paliperidone	0	0	0.6544	.	0.6544	.	.	.
clozapine:ziprasidone	0	0	1.3571	.	1.3571	.	.	.
lurasidone:molindone	0	0	0.8071	.	0.8071	.	.	.
lurasidone:olanzapine	0	0	-1.1289	.	-1.1289	.	.	.
lurasidone:placebo	2	1.00	0.1076	0.1076	.	.	.	.
lurasidone:quetiapine	0	0	-1.9247	.	-1.9247	.	.	.
lurasidone:risperidone/paliperidone	0	0	-0.4950	.	-0.4950	.	.	.
lurasidone:ziprasidone	0	0	0.2076	.	0.2076	.	.	.
molindone:olanzapine	1	0.82	-1.9360	-2.0500	-1.4217	-0.6283	-0.94	0.3465
molindone:placebo	0	0	-0.6995	.	-0.6995	.	.	.
molindone:quetiapine	0	0	-2.7318	.	-2.7318	.	.	.
molindone:risperidone/paliperidone	1	0.72	-1.3021	-1.1500	-1.7011	0.5511	0.94	0.3465
molindone:ziprasidone	0	0	-0.5995	.	-0.5995	.	.	.
olanzapine:placebo	2	0.89	1.2365	1.2029	1.5227	-0.3198	-0.94	0.3465
olanzapine:quetiapine	0	0	-0.7958	.	-0.7958	.	.	.
olanzapine:risperidone/paliperidone	1	0.17	0.6338	0.9000	0.5802	0.3198	0.94	0.3465
olanzapine:ziprasidone	0	0	1.3365	.	1.3365	.	.	.
placebo:quetiapine	0	0	-2.0323	.	-2.0323	.	.	.
placebo:risperidone/paliperidone	3	0.61	-0.6027	-0.5549	-0.6781	0.1232	0.73	0.4660
placebo:ziprasidone	1	1.00	0.1000	0.1000	.	.	.	.
quetiapine:risperidone/paliperidone	0	0	1.4296	.	1.4296	.	.	.
quetiapine:ziprasidone	0	0	2.1323	.	2.1323	.	.	.
risperidone/paliperidone:ziprasidone	0	0	0.7027	.	0.7027	.	.	.

Legend:

```
 comparison - Treatment comparison
 k          - Number of studies providing direct evidence
 prop       - Direct evidence proportion
 nma        - Estimated treatment effect (MD) in network meta-analysis
 direct     - Estimated treatment effect (MD) derived from direct evidence
 indir.     - Estimated treatment effect (MD) derived from indirect evidence
 Diff       - Difference between direct and indirect treatment estimates
 z          - z-value of test for disagreement (direct versus indirect)
 p-value    - p-value of test for disagreement (direct versus indirect)
```

eTable 28. 'Hot spots' of inconsistency: Triglycerides

Separate indirect from direct evidence (SIDE) using back-calculation method

Common effects model:

comparison	k	prop	nma	direct	indir.	Diff	z	p-value
asenapine:aripiprazole	0	0	0.0050	.	0.0050	.	.	.
blonanserin:aripiprazole	0	0	-0.0438	.	-0.0438	.	.	.
clozapine:aripiprazole	0	0	0.3441	.	0.3441	.	.	.
lurasidone:aripiprazole	0	0	-0.2121	.	-0.2121	.	.	.
molindone:aripiprazole	0	0	-0.1379	.	-0.1379	.	.	.
olanzapine:aripiprazole	1	0.64	0.1159	0.0654	0.2072	-0.1417	-0.95	0.3407
placebo:aripiprazole	4	0.33	-0.1799	-0.0675	-0.2361	0.1686	2.31	0.0211
quetiapine:aripiprazole	1	1.00	0.0057	0.0055	0.1981	-0.1925	-2.78	0.0055
risperidone/paliperidone:aripiprazole	2	0.64	0.0202	0.0549	-0.0406	0.0954	1.14	0.2541
ziprasidone:aripiprazole	0	0	-0.2111	.	-0.2111	.	.	.
asenapine:blonanserin	0	0	0.0487	.	0.0487	.	.	.
asenapine:clozapine	0	0	-0.3392	.	-0.3392	.	.	.
asenapine:lurasidone	0	0	0.2171	.	0.2171	.	.	.
asenapine:molindone	0	0	0.1429	.	0.1429	.	.	.
asenapine:olanzapine	0	0	-0.1109	.	-0.1109	.	.	.
asenapine:placebo	2	1.00	0.1849	0.1849	.	.	.	.
asenapine:quetiapine	0	0	-0.0007	.	-0.0007	.	.	.
asenapine:risperidone/paliperidone	0	0	-0.0153	.	-0.0153	.	.	.
asenapine:ziprasidone	0	0	0.2160	.	0.2160	.	.	.
blonanserin:clozapine	0	0	-0.3879	.	-0.3879	.	.	.
blonanserin:lurasidone	0	0	0.1683	.	0.1683	.	.	.
blonanserin:molindone	0	0	0.0941	.	0.0941	.	.	.
blonanserin:olanzapine	0	0	-0.1596	.	-0.1596	.	.	.
blonanserin:placebo	1	1.00	0.1361	0.1361	.	.	.	.
blonanserin:quetiapine	0	0	-0.0495	.	-0.0495	.	.	.
blonanserin:risperidone/paliperidone	0	0	-0.0640	.	-0.0640	.	.	.
blonanserin:ziprasidone	0	0	0.1673	.	0.1673	.	.	.
clozapine:lurasidone	0	0	0.5562	.	0.5562	.	.	.
clozapine:molindone	0	0	0.4820	.	0.4820	.	.	.
clozapine:olanzapine	1	1.00	0.2283	0.2283	.	.	.	.
clozapine:placebo	0	0	0.5240	.	0.5240	.	.	.
clozapine:quetiapine	0	0	0.3384	.	0.3384	.	.	.
clozapine:risperidone/paliperidone	0	0	0.3239	.	0.3239	.	.	.
clozapine:ziprasidone	0	0	0.5552	.	0.5552	.	.	.
lurasidone:molindone	0	0	-0.0742	.	-0.0742	.	.	.
lurasidone:olanzapine	0	0	-0.3280	.	-0.3280	.	.	.
lurasidone:placebo	2	1.00	-0.0322	-0.0322	.	.	.	.
lurasidone:quetiapine	0	0	-0.2178	.	-0.2178	.	.	.
lurasidone:risperidone/paliperidone	0	0	-0.2323	.	-0.2323	.	.	.
lurasidone:ziprasidone	0	0	-0.0010	.	-0.0010	.	.	.
molindone:olanzapine	1	0.58	-0.2538	-0.3096	-0.1769	-0.1327	-0.58	0.5610
molindone:placebo	0	0	0.0420	.	0.0420	.	.	.
molindone:quetiapine	0	0	-0.1436	.	-0.1436	.	.	.
molindone:risperidone/paliperidone	1	0.95	-0.1582	-0.1458	-0.4138	0.2680	0.58	0.5610
molindone:ziprasidone	0	0	0.0731	.	0.0731	.	.	.
olanzapine:placebo	1	0.15	0.2958	0.4204	0.2740	0.1464	0.70	0.4842
olanzapine:quetiapine	0	0	0.1102	.	0.1102	.	.	.
olanzapine:risperidone/paliperidone	2	0.80	0.0956	0.0810	0.1525	-0.0715	-0.40	0.6906
olanzapine:ziprasidone	0	0	0.3269	.	0.3269	.	.	.
placebo:quetiapine	4	0.45	-0.1856	-0.2924	-0.0998	-0.1925	-2.78	0.0055
placebo:risperidone/paliperidone	4	0.53	-0.2002	-0.1599	-0.2449	0.0851	1.00	0.3156
placebo:ziprasidone	3	1.00	0.0312	0.0312	.	.	.	.
quetiapine:risperidone/paliperidone	0	0	-0.0145	.	-0.0145	.	.	.
quetiapine:ziprasidone	0	0	0.2168	.	0.2168	.	.	.
risperidone/paliperidone:ziprasidone	0	0	0.2313	.	0.2313	.	.	.

Random effects model:

comparison	k	prop	nma	direct	indir.	Diff	z	p-value
asenapine:aripiprazole	0	0	0.0130	.	0.0130	.	.	.
blonanserin:aripiprazole	0	0	-0.0313	.	-0.0313	.	.	.
clozapine:aripiprazole	0	0	0.3495	.	0.3495	.	.	.
lurasidone:aripiprazole	0	0	-0.1972	.	-0.1972	.	.	.
molindone:aripiprazole	0	0	-0.1346	.	-0.1346	.	.	.
olanzapine:aripiprazole	1	0.63	0.1213	0.0654	0.2161	-0.1507	-0.97	0.3312
placebo:aripiprazole	4	0.37	-0.1675	-0.0697	-0.2260	0.1563	1.94	0.0518
quetiapine:aripiprazole	1	0.84	0.0364	0.0055	0.1959	-0.1904	-2.37	0.0176
risperidone/paliperidone:aripiprazole	2	0.63	0.0245	0.0555	-0.0285	0.0840	0.92	0.3565

ziprasidone:aripiprazole	0	0	-0.1971	.	-0.1971	.	.	.
asenapine:blonanserin	0	0	0.0444	.	0.0444	.	.	.
asenapine:clozapine	0	0	-0.3365	.	-0.3365	.	.	.
asenapine:lurasidone	0	0	0.2103	.	0.2103	.	.	.
asenapine:molindone	0	0	0.1476	.	0.1476	.	.	.
asenapine:olanzapine	0	0	-0.1082	.	-0.1082	.	.	.
asenapine:placebo	2	1.00	0.1805	0.1805	.	.	.	.
asenapine:quetiapine	0	0	-0.0234	.	-0.0234	.	.	.
asenapine:risperidone/paliperidone	0	0	-0.0114	.	-0.0114	.	.	.
asenapine:ziprasidone	0	0	0.2101	.	0.2101	.	.	.
blonanserin:clozapine	0	0	-0.3809	.	-0.3809	.	.	.
blonanserin:lurasidone	0	0	0.1659	.	0.1659	.	.	.
blonanserin:molindone	0	0	0.1033	.	0.1033	.	.	.
blonanserin:olanzapine	0	0	-0.1526	.	-0.1526	.	.	.
blonanserin:placebo	1	1.00	0.1361	0.1361	.	.	.	.
blonanserin:quetiapine	0	0	-0.0677	.	-0.0677	.	.	.
blonanserin:risperidone/paliperidone	0	0	-0.0558	.	-0.0558	.	.	.
blonanserin:ziprasidone	0	0	0.1657	.	0.1657	.	.	.
clozapine:lurasidone	0	0	0.5467	.	0.5467	.	.	.
clozapine:molindone	0	0	0.4841	.	0.4841	.	.	.
clozapine:olanzapine	1	1.00	0.2283	0.2283	.	.	.	.
clozapine:placebo	0	0	0.5170	.	0.5170	.	.	.
clozapine:quetiapine	0	0	0.3131	.	0.3131	.	.	.
clozapine:risperidone/paliperidone	0	0	0.3251	.	0.3251	.	.	.
clozapine:ziprasidone	0	0	0.5466	.	0.5466	.	.	.
lurasidone:molindone	0	0	-0.0626	.	-0.0626	.	.	.
lurasidone:olanzapine	0	0	-0.3185	.	-0.3185	.	.	.
lurasidone:placebo	2	1.00	-0.0297	-0.0297	.	.	.	.
lurasidone:quetiapine	0	0	-0.2336	.	-0.2336	.	.	.
lurasidone:risperidone/paliperidone	0	0	-0.2217	.	-0.2217	.	.	.
lurasidone:ziprasidone	0	0	-0.0001	.	-0.0001	.	.	.
molindone:olanzapine	1	0.60	-0.2559	-0.3096	-0.1761	-0.1335	-0.56	0.5762
molindone:placebo	0	0	0.0329	.	0.0329	.	.	.
molindone:quetiapine	0	0	-0.1710	.	-0.1710	.	.	.
molindone:risperidone/paliperidone	1	0.95	-0.1591	-0.1458	-0.4013	0.2556	0.56	0.5762
molindone:ziprasidone	0	0	0.0625	.	0.0625	.	.	.
olanzapine:placebo	1	0.16	0.2887	0.4204	0.2637	0.1566	0.73	0.4632
olanzapine:quetiapine	0	0	0.0849	.	0.0849	.	.	.
olanzapine:risperidone/paliperidone	2	0.79	0.0968	0.0824	0.1518	-0.0695	-0.37	0.7077
olanzapine:ziprasidone	0	0	0.3183	.	0.3183	.	.	.
placebo:quetiapine	4	0.54	-0.2039	-0.2914	-0.1010	-0.1904	-2.37	0.0176
placebo:risperidone/paliperidone	4	0.55	-0.1919	-0.1588	-0.2332	0.0744	0.81	0.4172
placebo:ziprasidone	3	1.00	0.0296	0.0296	.	.	.	.
quetiapine:risperidone/paliperidone	0	0	0.0119	.	0.0119	.	.	.
quetiapine:ziprasidone	0	0	0.2335	.	0.2335	.	.	.
risperidone/paliperidone:ziprasidone	0	0	0.2216	.	0.2216	.	.	.

Legend:
- comparison - Treatment comparison
- k - Number of studies providing direct evidence
- prop - Direct evidence proportion
- nma - Estimated treatment effect (MD) in network meta-analysis
- direct - Estimated treatment effect (MD) derived from direct evidence
- indir. - Estimated treatment effect (MD) derived from indirect evidence
- Diff - Difference between direct and indirect treatment estimates
- z - z-value of test for disagreement (direct versus indirect)
- p-value - p-value of test for disagreement (direct versus indirect)

eTable 29. 'Hot spots' of inconsistency: prolactin

Separate indirect from direct evidence (SIDE) using back-calculation method

Common effects model:

comparison	k	prop	nma	direct	indir.	Diff	z	p-value
asenapine:aripiprazole	0	0	5.3490	.	5.3490	.	.	.
blonanserin:aripiprazole	0	0	13.6252	.	13.6252	.	.	.
lurasidone:aripiprazole	0	0	5.6731	.	5.6731	.	.	.
molindone:aripiprazole	0	0	-0.2370	.	-0.2370	.	.	.

```
              olanzapine:aripiprazole 0    0  14.6708        .  14.6708        .     .       .
                 placebo:aripiprazole 5 0.88   4.3560   4.9564  -0.2316   5.1880  3.34  0.0008
              quetiapine:aripiprazole 1 0.32   4.9915   0.1880   7.2581  -7.0701 -3.54  0.0004
 risperidone_paliperidone:aripiprazole 2 0.29  20.7057  19.2773  21.2809  -2.0036 -0.88  0.3811
                 asenapine:blonanserin 0    0  -8.2762        .  -8.2762        .     .       .
                  asenapine:lurasidone 0    0  -0.3241        .  -0.3241        .     .       .
                   asenapine:molindone 0    0   5.5860        .   5.5860        .     .       .
                  asenapine:olanzapine 0    0  -9.3218        .  -9.3218        .     .       .
                     asenapine:placebo 2 1.00   0.9930   0.9930        .        .     .       .
                  asenapine:quetiapine 0    0   0.3575        .   0.3575        .     .       .
    asenapine:risperidone_paliperidone 0    0 -15.3567        . -15.3567        .     .       .
                blonanserin:lurasidone 0    0   7.9521        .   7.9521        .     .       .
                 blonanserin:molindone 0    0  13.8622        .  13.8622        .     .       .
                blonanserin:olanzapine 0    0  -1.0456        .  -1.0456        .     .       .
                   blonanserin:placebo 1 1.00   9.2692   9.2692        .        .     .       .
                blonanserin:quetiapine 0    0   8.6338        .   8.6338        .     .       .
  blonanserin:risperidone_paliperidone 0    0  -7.0805        .  -7.0805        .     .       .
                  lurasidone:molindone 0    0   5.9101        .   5.9101        .     .       .
                 lurasidone:olanzapine 0    0  -8.9977        .  -8.9977        .     .       .
                    lurasidone:placebo 3 1.00   1.3171   1.3171        .        .     .       .
                 lurasidone:quetiapine 0    0   0.6816        .   0.6816        .     .       .
   lurasidone:risperidone_paliperidone 0    0 -15.0326        . -15.0326        .     .       .
                  molindone:olanzapine 1 0.80 -14.9078  -7.3000 -46.3036  39.0036  3.31  0.0009
                     molindone:placebo 0    0  -4.5930        .  -4.5930        .     .       .
                  molindone:quetiapine 0    0  -5.2285        .  -5.2285        .     .       .
    molindone:risperidone_paliperidone 1 0.81 -20.9427 -28.3000  11.4616 -39.7616 -3.31  0.0009
                    olanzapine:placebo 2 0.93  10.3148  11.4497  -5.2933  16.7430  3.31  0.0009
                 olanzapine:quetiapine 0    0   9.6793        .   9.6793        .     .       .
   olanzapine:risperidone_paliperidone 1 0.11  -6.0349 -21.0000  -4.2570 -16.7430 -3.31  0.0009
                    placebo:quetiapine 4 0.75  -0.6355  -2.4348   4.6353  -7.0701 -3.54  0.0004
      placebo:risperidone_paliperidone 8 0.72 -16.3497 -15.9968 -17.2762   1.2794  0.59  0.5569
   quetiapine:risperidone_paliperidone 0    0 -15.7142        . -15.7142        .     .       .
```

Random effects model:

```
                            comparison k prop      nma   direct   indir.     Diff     z p-value
                asenapine:aripiprazole 0    0   3.3272        .   3.3272        .     .       .
              blonanserin:aripiprazole 0    0  12.1034        .  12.1034        .     .       .
               lurasidone:aripiprazole 0    0   4.7930        .   4.7930        .     .       .
                molindone:aripiprazole 0    0   3.1452        .   3.1452        .     .       .
               olanzapine:aripiprazole 0    0  12.6735        .  12.6735        .     .       .
                  placebo:aripiprazole 5 0.70   2.8342   6.0219  -4.4878  10.5097  1.69  0.0902
               quetiapine:aripiprazole 1 0.33   3.7128   0.1880   5.4777  -5.2897 -0.57  0.5713
 risperidone_paliperidone:aripiprazole 2 0.37  29.2387  22.4274  33.2532 -10.8258 -1.52  0.1292
                 asenapine:blonanserin 0    0  -8.7762        .  -8.7762        .     .       .
                  asenapine:lurasidone 0    0  -1.4658        .  -1.4658        .     .       .
                   asenapine:molindone 0    0   0.1819        .   0.1819        .     .       .
                  asenapine:olanzapine 0    0  -9.3463        .  -9.3463        .     .       .
                     asenapine:placebo 2 1.00   0.4930   0.4930        .        .     .       .
                  asenapine:quetiapine 0    0  -0.3856        .  -0.3856        .     .       .
    asenapine:risperidone_paliperidone 0    0 -25.9116        . -25.9116        .     .       .
                blonanserin:lurasidone 0    0   7.3104        .   7.3104        .     .       .
                 blonanserin:molindone 0    0   8.9581        .   8.9581        .     .       .
                blonanserin:olanzapine 0    0  -0.5701        .  -0.5701        .     .       .
                   blonanserin:placebo 1 1.00   9.2692   9.2692        .        .     .       .
                blonanserin:quetiapine 0    0   8.3906        .   8.3906        .     .       .
  blonanserin:risperidone_paliperidone 0    0 -17.1353        . -17.1353        .     .       .
                  lurasidone:molindone 0    0   1.6477        .   1.6477        .     .       .
                 lurasidone:olanzapine 0    0  -7.8805        .  -7.8805        .     .       .
                    lurasidone:placebo 3 1.00   1.9588   1.9588        .        .     .       .
                 lurasidone:quetiapine 0    0   1.0802        .   1.0802        .     .       .
   lurasidone:risperidone_paliperidone 0    0 -24.4458        . -24.4458        .     .       .
                  molindone:olanzapine 1 0.84  -9.5282  -7.3000 -21.4115  14.1115  0.62  0.5373
                     molindone:placebo 0    0   0.3111        .   0.3111        .     .       .
                  molindone:quetiapine 0    0  -0.5675        .  -0.5675        .     .       .
    molindone:risperidone_paliperidone 1 0.84 -26.0935 -28.3000 -14.1159 -14.1841 -0.62  0.5373
                    olanzapine:placebo 2 0.72   9.8393  11.6948   4.9671   6.7277  0.62  0.5373
                 olanzapine:quetiapine 0    0   8.9607        .   8.9607        .     .       .
   olanzapine:risperidone_paliperidone 1 0.34 -16.5652 -21.0000 -14.2723  -6.7277 -0.62  0.5373
                    placebo:quetiapine 4 0.77  -0.8786  -2.0941   3.1957  -5.2897 -0.57  0.5713
      placebo:risperidone_paliperidone 8 0.76 -26.4046 -27.8824 -21.6313  -6.2511 -0.99  0.3233
   quetiapine:risperidone_paliperidone 0    0 -25.5259        . -25.5259        .     .       .
```

Legend:

```
 comparison - Treatment comparison
 k          - Number of studies providing direct evidence
 prop       - Direct evidence proportion
 nma        - Estimated treatment effect (MD) in network meta-analysis
 direct     - Estimated treatment effect (MD) derived from direct evidence
 indir.     - Estimated treatment effect (MD) derived from indirect evidence
 Diff       - Difference between direct and indirect treatment estimates
 z          - z-value of test for disagreement (direct versus indirect)
 p-value    - p-value of test for disagreement (direct versus indirect)
```

eTable 30. 'Hot spots' of inconsistency: glucose

Separate indirect from direct evidence (SIDE) using back-calculation method

Common effects model:

comparison	k	prop	nma	direct	indir.	Diff	z	p-value
asenapine:aripiprazole	0	0	0.1407	.	0.1407	.	.	.
blonanserin:aripiprazole	0	0	-0.1120	.	-0.1120	.	.	.
clozapine:aripiprazole	0	0	0.5546	.	0.5546	.	.	.
lurasidone:aripiprazole	0	0	-0.0186	.	-0.0186	.	.	.
molindone:aripiprazole	0	0	0.0993	.	0.0993	.	.	.
olanzapine:aripiprazole	1	0.26	-0.0060	0.0517	-0.0267	0.0784	0.83	0.4053
placebo:aripiprazole	4	0.36	-0.0263	-0.0863	0.0078	-0.0941	-1.09	0.2758
quetiapine:aripiprazole	1	0.20	-0.0042	0.0600	-0.0203	0.0803	0.77	0.4398
risperidone/paliperidone:aripiprazole	3	0.72	0.1323	0.1226	0.1577	-0.0351	-0.35	0.7297
ziprasidone:aripiprazole	0	0	-0.0275	.	-0.0275	.	.	.
asenapine:blonanserin	0	0	0.2527	.	0.2527	.	.	.
asenapine:clozapine	0	0	-0.4139	.	-0.4139	.	.	.
asenapine:lurasidone	0	0	0.1593	.	0.1593	.	.	.
asenapine:molindone	0	0	0.0414	.	0.0414	.	.	.
asenapine:olanzapine	0	0	0.1467	.	0.1467	.	.	.
asenapine:placebo	2	1.00	0.1670	0.1670	.	.	.	.
asenapine:quetiapine	0	0	0.1449	.	0.1449	.	.	.
asenapine:risperidone/paliperidone	0	0	0.0084	.	0.0084	.	.	.
asenapine:ziprasidone	0	0	0.1682	.	0.1682	.	.	.
blonanserin:clozapine	0	0	-0.6666	.	-0.6666	.	.	.
blonanserin:lurasidone	0	0	-0.0934	.	-0.0934	.	.	.
blonanserin:molindone	0	0	-0.2113	.	-0.2113	.	.	.
blonanserin:olanzapine	0	0	-0.1060	.	-0.1060	.	.	.
blonanserin:placebo	1	1.00	-0.0857	-0.0857	.	.	.	.
blonanserin:quetiapine	0	0	-0.1078	.	-0.1078	.	.	.
blonanserin:risperidone/paliperidone	0	0	-0.2443	.	-0.2443	.	.	.
blonanserin:ziprasidone	0	0	-0.0845	.	-0.0845	.	.	.
clozapine:lurasidone	0	0	0.5732	.	0.5732	.	.	.
clozapine:molindone	0	0	0.4553	.	0.4553	.	.	.
clozapine:olanzapine	1	1.00	0.5606	0.5606	.	.	.	.
clozapine:placebo	0	0	0.5809	.	0.5809	.	.	.
clozapine:quetiapine	0	0	0.5588	.	0.5588	.	.	.
clozapine:risperidone/paliperidone	0	0	0.4223	.	0.4223	.	.	.
clozapine:ziprasidone	0	0	0.5821	.	0.5821	.	.	.
lurasidone:molindone	0	0	-0.1179	.	-0.1179	.	.	.
lurasidone:olanzapine	0	0	-0.0126	.	-0.0126	.	.	.
lurasidone:placebo	2	1.00	0.0077	0.0077	.	.	.	.
lurasidone:quetiapine	0	0	-0.0144	.	-0.0144	.	.	.
lurasidone:risperidone/paliperidone	0	0	-0.1509	.	-0.1509	.	.	.
lurasidone:ziprasidone	0	0	0.0089	.	0.0089	.	.	.
molindone:olanzapine	1	0.49	0.1053	0.0166	0.1888	-0.1722	-0.68	0.4951
molindone:placebo	0	0	0.1256	.	0.1256	.	.	.
molindone:quetiapine	0	0	0.1035	.	0.1035	.	.	.
molindone:risperidone/paliperidone	1	0.96	-0.0330	-0.0167	-0.4461	0.4295	0.68	0.4951
molindone:ziprasidone	0	0	0.1268	.	0.1268	.	.	.
olanzapine:placebo	2	1.00	0.0203	0.0201	0.1517	-0.1316	-1.65	0.0984
olanzapine:quetiapine	0	0	-0.0018	.	-0.0018	.	.	.
olanzapine:risperidone/paliperidone	2	0.38	-0.1383	-0.0250	-0.2078	0.1828	1.97	0.0484
olanzapine:ziprasidone	0	0	0.0215	.	0.0215	.	.	.
placebo:quetiapine	3	1.00	-0.0221	-0.0220	-0.1023	0.0803	0.77	0.4398
placebo:risperidone/paliperidone	2	0.38	-0.1586	-0.2423	-0.1083	-0.1340	-1.44	0.1486
placebo:ziprasidone	3	1.00	0.0012	0.0012	.	.	.	.
quetiapine:risperidone/paliperidone	0	0	-0.1365	.	-0.1365	.	.	.
quetiapine:ziprasidone	0	0	0.0233	.	0.0233	.	.	.
risperidone/paliperidone:ziprasidone	0	0	0.1598	.	0.1598	.	.	.

Random effects model:

comparison	k	prop	nma	direct	indir.	Diff	z	p-value
asenapine:aripiprazole	0	0	0.0659	.	0.0659	.	.	.
blonanserin:aripiprazole	0	0	-0.1923	.	-0.1923	.	.	.
clozapine:aripiprazole	0	0	0.6153	.	0.6153	.	.	.
lurasidone:aripiprazole	0	0	-0.0918	.	-0.0918	.	.	.
molindone:aripiprazole	0	0	0.0885	.	0.0885	.	.	.
olanzapine:aripiprazole	1	0.33	0.0548	0.0517	0.0563	-0.0046	-0.01	0.9956
placebo:aripiprazole	4	0.60	-0.1066	0.0259	-0.3048	0.3306	0.58	0.5632
quetiapine:aripiprazole	1	0.34	0.6289	0.0600	0.9223	-0.8623	-1.01	0.3145
risperidone/paliperidone:aripiprazole	3	0.64	0.1210	0.1428	0.0817	0.0610	0.09	0.9278
ziprasidone:aripiprazole	0	0	-0.1452	.	-0.1452	.	.	.
asenapine:blonanserin	0	0	0.2582	.	0.2582	.	.	.
asenapine:clozapine	0	0	-0.5494	.	-0.5494	.	.	.
asenapine:lurasidone	0	0	0.1577	.	0.1577	.	.	.
asenapine:molindone	0	0	-0.0226	.	-0.0226	.	.	.
asenapine:olanzapine	0	0	0.0111	.	0.0111	.	.	.
asenapine:placebo	2	1.00	0.1725	0.1725	.	.	.	.
asenapine:quetiapine	0	0	-0.5630	.	-0.5630	.	.	.
asenapine:risperidone/paliperidone	0	0	-0.0551	.	-0.0551	.	.	.

asenapine:ziprasidone	0	0	0.2111	.	0.2111	.	.	.
blonanserin:clozapine	0	0	-0.8076	.	-0.8076	.	.	.
blonanserin:lurasidone	0	0	-0.1004	.	-0.1004	.	.	.
blonanserin:molindone	0	0	-0.2807	.	-0.2807	.	.	.
blonanserin:olanzapine	0	0	-0.2471	.	-0.2471	.	.	.
blonanserin:placebo	1	1.00	-0.0857	-0.0857	.	.	.	.
blonanserin:quetiapine	0	0	-0.8212	.	-0.8212	.	.	.
blonanserin:risperidone/paliperidone	0	0	-0.3132	.	-0.3132	.	.	.
blonanserin:ziprasidone	0	0	-0.0471	.	-0.0471	.	.	.
clozapine:lurasidone	0	0	0.7072	.	0.7072	.	.	.
clozapine:molindone	0	0	0.5269	.	0.5269	.	.	.
clozapine:olanzapine	1	1.00	0.5605	0.5606	.	.	.	.
clozapine:placebo	0	0	0.7219	.	0.7219	.	.	.
clozapine:quetiapine	0	0	-0.0136	.	-0.0136	.	.	.
clozapine:risperidone/paliperidone	0	0	0.4944	.	0.4944	.	.	.
clozapine:ziprasidone	0	0	0.7605	.	0.7605	.	.	.
lurasidone:molindone	0	0	-0.1803	.	-0.1803	.	.	.
lurasidone:olanzapine	0	0	-0.1466	.	-0.1466	.	.	.
lurasidone:placebo	2	1.00	0.0148	0.0148	.	.	.	.
lurasidone:quetiapine	0	0	-0.7208	.	-0.7208	.	.	.
lurasidone:risperidone/paliperidone	0	0	-0.2128	.	-0.2128	.	.	.
lurasidone:ziprasidone	0	0	0.0534	.	0.0534	.	.	.
molindone:olanzapine	1	0.81	0.0337	0.0166	0.1080	-0.0913	-0.06	0.9559
molindone:placebo	0	0	0.1950	.	0.1950	.	.	.
molindone:quetiapine	0	0	-0.5405	.	-0.5405	.	.	.
molindone:risperidone/paliperidone	1	0.83	-0.0325	-0.0167	-0.1112	0.0946	0.06	0.9559
molindone:ziprasidone	0	0	0.2337	.	0.2337	.	.	.
olanzapine:placebo	2	0.55	0.1614	0.1318	0.1981	-0.0663	-0.09	0.9286
olanzapine:quetiapine	0	0	-0.5742	.	-0.5742	.	.	.
olanzapine:risperidone/paliperidone	2	0.60	-0.0662	-0.0279	-0.1238	0.0959	0.12	0.9029
olanzapine:ziprasidone	0	0	0.2000	.	0.2000	.	.	.
placebo:quetiapine	3	0.78	-0.7355	-0.9240	-0.0617	-0.8623	-1.01	0.3145
placebo:risperidone/paliperidone	2	0.44	-0.2276	-0.2175	-0.2354	0.0178	0.03	0.9784
placebo:ziprasidone	3	1.00	0.0386	0.0386	.	.	.	.
quetiapine:risperidone/paliperidone	0	0	0.5080	.	0.5080	.	.	.
quetiapine:ziprasidone	0	0	0.7741	.	0.7741	.	.	.
risperidone/paliperidone:ziprasidone	0	0	0.2662	.	0.2662	.	.	.

Legend:
comparison - Treatment comparison
k - Number of studies providing direct evidence
prop - Direct evidence proportion
nma - Estimated treatment effect (MD) in network meta-analysis
direct - Estimated treatment effect (MD) derived from direct evidence
indir. - Estimated treatment effect (MD) derived from indirect evidence
Diff - Difference between direct and indirect treatment estimates
z - z-value of test for disagreement (direct versus indirect)
p-value - p-value of test for disagreement (direct versus indirect)

eTable 31. 'Hot spots' of inconsistency: total cholesterol

Separate indirect from direct evidence (SIDE) using back-calculation method

Common effects model:

comparison	k	prop	nma	direct	indir.	Diff	z	p-value
asenapine:aripiprazole	0	0	0.1555	.	0.1555	.	.	.
blonanserin:aripiprazole	0	0	-0.1165	.	-0.1165	.	.	.
clozapine:aripiprazole	0	0	0.1943	.	0.1943	.	.	.
lurasidone:aripiprazole	0	0	-0.0674	.	-0.0674	.	.	.
molindone:aripiprazole	0	0	-0.0241	.	-0.0241	.	.	.
olanzapine:aripiprazole	1	0.62	0.2254	0.0135	0.5721	-0.5586	-3.73	0.0002
placebo:aripiprazole	3	0.51	-0.0793	-0.1172	-0.0397	-0.0775	-0.78	0.4374
quetiapine:aripiprazole	1	0.20	0.2272	0.6300	0.1280	0.5020	3.10	0.0019
risperidone/paliperidone:aripiprazole	2	0.66	-0.0048	0.0011	-0.0165	0.0177	0.17	0.8655
ziprasidone:aripiprazole	0	0	-0.0727	.	-0.0727	.	.	.
asenapine:blonanserin	0	0	0.2721	.	0.2721	.	.	.
asenapine:clozapine	0	0	-0.0388	.	-0.0388	.	.	.
asenapine:lurasidone	0	0	0.2229	.	0.2229	.	.	.
asenapine:molindone	0	0	0.1796	.	0.1796	.	.	.
asenapine:olanzapine	0	0	-0.0698	.	-0.0698	.	.	.
asenapine:placebo	2	1.00	0.2348	0.2348	.	.	.	.
asenapine:quetiapine	0	0	-0.0717	.	-0.0717	.	.	.
asenapine:risperidone/paliperidone	0	0	0.1604	.	0.1604	.	.	.
asenapine:ziprasidone	0	0	0.2282	.	0.2282	.	.	.
blonanserin:clozapine	0	0	-0.3108	.	-0.3108	.	.	.
blonanserin:lurasidone	0	0	-0.0491	.	-0.0491	.	.	.
blonanserin:molindone	0	0	-0.0925	.	-0.0925	.	.	.
blonanserin:olanzapine	0	0	-0.3419	.	-0.3419	.	.	.
blonanserin:placebo	1	1.00	-0.0372	-0.0372	.	.	.	.
blonanserin:quetiapine	0	0	-0.3437	.	-0.3437	.	.	.
blonanserin:risperidone/paliperidone	0	0	-0.1117	.	-0.1117	.	.	.
blonanserin:ziprasidone	0	0	-0.0438	.	-0.0438	.	.	.
clozapine:lurasidone	0	0	0.2617	.	0.2617	.	.	.
clozapine:molindone	0	0	0.2183	.	0.2183	.	.	.

```
               clozapine:olanzapine 1 1.00 -0.0311 -0.0311       .       .     .        .
                  clozapine:placebo 0    0  0.2736       .  0.2736       .     .        .
               clozapine:quetiapine 0    0 -0.0329       . -0.0329       .     .        .
  clozapine:risperidone/paliperidone 0    0  0.1991       .  0.1991       .     .        .
             clozapine:ziprasidone 0    0  0.2670       .  0.2670       .     .        .
              lurasidone:molindone 0    0 -0.0434       . -0.0434       .     .        .
             lurasidone:olanzapine 0    0 -0.2928       . -0.2928       .     .        .
                lurasidone:placebo 2 1.00  0.0119  0.0119       .       .     .        .
             lurasidone:quetiapine 0    0 -0.2946       . -0.2946       .     .        .
 lurasidone:risperidone/paliperidone 0    0 -0.0626       . -0.0626       .     .        .
            lurasidone:ziprasidone 0    0  0.0053       .  0.0053       .     .        .
              molindone:olanzapine 1 0.73 -0.2494 -0.5154  0.4750 -0.9904 -4.16 < 0.0001
                 molindone:placebo 0    0  0.0553       .  0.0553       .     .        .
              molindone:quetiapine 0    0 -0.2512       . -0.2512       .     .        .
  molindone:risperidone/paliperidone 1 0.71 -0.0192  0.2642 -0.7091  0.9732  4.16 < 0.0001
             molindone:ziprasidone 0    0  0.0487       .  0.0487       .     .        .
                olanzapine:placebo 1 0.24  0.3047  0.2201  0.3310 -0.1109 -0.62   0.5344
             olanzapine:quetiapine 0    0 -0.0018       . -0.0018       .     .        .
 olanzapine:risperidone/paliperidone 2 0.72  0.2302  0.3648 -0.1164  0.4812  3.01   0.0026
            olanzapine:ziprasidone 0    0  0.2981       .  0.2981       .     .        .
                placebo:quetiapine 3 0.91 -0.3065 -0.2609 -0.7628  0.5020  3.10   0.0019
    placebo:risperidone/paliperidone 4 0.63 -0.0745 -0.1326  0.0230 -0.1556 -1.52   0.1279
               placebo:ziprasidone 3 1.00 -0.0066 -0.0066       .       .     .        .
 quetiapine:risperidone/paliperidone 0    0  0.2320       .  0.2320       .     .        .
            quetiapine:ziprasidone 0    0  0.2999       .  0.2999       .     .        .
 risperidone/paliperidone:ziprasidone 0    0  0.0679       .  0.0679       .     .        .

Random effects model:

                          comparison k prop     nma  direct  indir.    Diff     z p-value
            asenapine:aripiprazole 0    0  0.1825       .  0.1825       .     .       .
          blonanserin:aripiprazole 0    0 -0.0903       . -0.0903       .     .       .
            clozapine:aripiprazole 0    0  0.2492       .  0.2492       .     .       .
           lurasidone:aripiprazole 0    0 -0.0296       . -0.0296       .     .       .
            molindone:aripiprazole 0    0  0.0006       .  0.0006       .     .       .
           olanzapine:aripiprazole 1 0.50  0.2803  0.0135  0.5469 -0.5335 -2.11  0.0352
              placebo:aripiprazole 3 0.50 -0.0531 -0.1203  0.0146 -0.1349 -0.75  0.4518
           quetiapine:aripiprazole 1 0.31  0.2969  0.6300  0.1494  0.4806  1.89  0.0581
 risperidone/paliperidone:aripiprazole 2 0.59 -0.0204 -0.0053 -0.0419  0.0367  0.19  0.8498
          ziprasidone:aripiprazole 0    0 -0.0523       . -0.0523       .     .       .
             asenapine:blonanserin 0    0  0.2728       .  0.2728       .     .       .
               asenapine:clozapine 0    0 -0.0666       . -0.0666       .     .       .
              asenapine:lurasidone 0    0  0.2121       .  0.2121       .     .       .
               asenapine:molindone 0    0  0.1819       .  0.1819       .     .       .
              asenapine:olanzapine 0    0 -0.0977       . -0.0977       .     .       .
                 asenapine:placebo 2 1.00  0.2356  0.2356       .       .     .       .
              asenapine:quetiapine 0    0 -0.1143       . -0.1143       .     .       .
  asenapine:risperidone/paliperidone 0    0  0.2029       .  0.2029       .     .       .
             asenapine:ziprasidone 0    0  0.2348       .  0.2348       .     .       .
             blonanserin:clozapine 0    0 -0.3395       . -0.3395       .     .       .
            blonanserin:lurasidone 0    0 -0.0607       . -0.0607       .     .       .
             blonanserin:molindone 0    0 -0.0909       . -0.0909       .     .       .
            blonanserin:olanzapine 0    0 -0.3706       . -0.3706       .     .       .
               blonanserin:placebo 1 1.00 -0.0372 -0.0372       .       .     .       .
            blonanserin:quetiapine 0    0 -0.3872       . -0.3872       .     .       .
 blonanserin:risperidone/paliperidone 0    0 -0.0699       . -0.0699       .     .       .
           blonanserin:ziprasidone 0    0 -0.0380       . -0.0380       .     .       .
              clozapine:lurasidone 0    0  0.2787       .  0.2787       .     .       .
               clozapine:molindone 0    0  0.2486       .  0.2486       .     .       .
              clozapine:olanzapine 1 1.00 -0.0311 -0.0311       .       .     .       .
                 clozapine:placebo 0    0  0.3023       .  0.3023       .     .       .
              clozapine:quetiapine 0    0 -0.0477       . -0.0477       .     .       .
  clozapine:risperidone/paliperidone 0    0  0.2696       .  0.2696       .     .       .
             clozapine:ziprasidone 0    0  0.3015       .  0.3015       .     .       .
              lurasidone:molindone 0    0 -0.0302       . -0.0302       .     .       .
             lurasidone:olanzapine 0    0 -0.3098       . -0.3098       .     .       .
                lurasidone:placebo 2 1.00  0.0235  0.0235       .       .     .       .
             lurasidone:quetiapine 0    0 -0.3264       . -0.3264       .     .       .
 lurasidone:risperidone/paliperidone 0    0 -0.0092       . -0.0092       .     .       .
            lurasidone:ziprasidone 0    0  0.0227       .  0.0227       .     .       .
              molindone:olanzapine 1 0.80 -0.2797 -0.5154  0.6512 -1.1666 -2.66  0.0078
                 molindone:placebo 0    0  0.0537       .  0.0537       .     .       .
              molindone:quetiapine 0    0 -0.2963       . -0.2963       .     .       .
  molindone:risperidone/paliperidone 1 0.79  0.0210  0.2642 -0.8877  1.1519  2.66  0.0078
             molindone:ziprasidone 0    0  0.0529       .  0.0529       .     .       .
                olanzapine:placebo 1 0.32  0.3333  0.2202  0.3867 -0.1665 -0.63  0.5304
             olanzapine:quetiapine 0    0 -0.0166       . -0.0166       .     .       .
 olanzapine:risperidone/paliperidone 2 0.70  0.3007  0.4304 -0.0089  0.4393  1.70  0.0883
            olanzapine:ziprasidone 0    0  0.3325       .  0.3325       .     .       .
                placebo:quetiapine 3 0.84 -0.3500 -0.2728 -0.7534  0.4806  1.89  0.0581
    placebo:risperidone/paliperidone 4 0.64 -0.0327 -0.0997  0.0866 -0.1863 -1.02  0.3056
               placebo:ziprasidone 3 1.00 -0.0008 -0.0008       .       .     .       .
 quetiapine:risperidone/paliperidone 0    0  0.3173       .  0.3173       .     .       .
            quetiapine:ziprasidone 0    0  0.3492       .  0.3492       .     .       .
 risperidone/paliperidone:ziprasidone 0    0  0.0319       .  0.0319       .     .       .

Legend:
 comparison - Treatment comparison
```

k - Number of studies providing direct evidence
prop - Direct evidence proportion
nma - Estimated treatment effect (MD) in network meta-analysis
direct - Estimated treatment effect (MD) derived from direct evidence
indir. - Estimated treatment effect (MD) derived from indirect evidence
Diff - Difference between direct and indirect treatment estimates
z - z-value of test for disagreement (direct versus indirect)
p-value - p-value of test for disagreement (direct versus indirect)

eTable 32. ‘Hot spots’ of inconsistency: HDL cholesterol

Separate indirect from direct evidence (SIDE) using back-calculation method

Common effects model:

comparison	k	prop	nma	direct	indir.	Diff	z	p-value
molindone:aripiprazole	0	0	0.1715	.	0.1715	.	.	.
olanzapine:aripiprazole	1	0.72	0.0920	-0.0262	0.4011	-0.4273	-5.63	< 0.0001
placebo:aripiprazole	2	0.46	-0.3438	-0.1488	-0.5108	0.3620	5.79	< 0.0001
quetiapine:aripiprazole	1	0.25	-0.3172	0.0200	-0.4278	0.4478	5.73	< 0.0001
risperidone/paliperidone:aripiprazole	1	0.72	0.1698	-0.0373	0.7011	-0.7384	-11.09	< 0.0001
ziprasidone:aripiprazole	0	0	-0.2810	.	-0.2810	.	.	.
molindone:olanzapine	1	0.86	0.0795	-0.0207	0.7109	-0.7317	-4.78	< 0.0001
molindone:placebo	0	0	0.5152	.	0.5152	.	.	.
molindone:quetiapine	0	0	0.4887	.	0.4887	.	.	.
molindone:risperidone/paliperidone	1	0.84	0.0017	0.1114	-0.5830	0.6944	4.78	< 0.0001
molindone:ziprasidone	0	0	0.4525	.	0.4525	.	.	.
olanzapine:placebo	1	0.21	0.4358	-0.1010	0.5784	-0.6794	-7.26	< 0.0001
olanzapine:quetiapine	0	0	0.4092	.	0.4092	.	.	.
olanzapine:risperidone/paliperidone	2	0.91	-0.0778	0.0516	-1.3467	1.3984	13.98	< 0.0001
olanzapine:ziprasidone	0	0	0.3730	.	0.3730	.	.	.
placebo:quetiapine	4	0.95	-0.0266	-0.0048	-0.4526	0.4478	5.73	< 0.0001
placebo:risperidone/paliperidone	2	0.47	-0.5135	-1.1042	-0.0001	-1.1041	-16.40	< 0.0001
placebo:ziprasidone	3	1.00	-0.0627	-0.0627	.	.	.	.
quetiapine:risperidone/paliperidone	0	0	-0.4870	.	-0.4870	.	.	.
quetiapine:ziprasidone	0	0	-0.0362	.	-0.0362	.	.	.
risperidone/paliperidone:ziprasidone	0	0	0.4508	.	0.4508	.	.	.

Random effects model:

comparison	k	prop	nma	direct	indir.	Diff	z	p-value
molindone:aripiprazole	0	0	0.2260	.	0.2260	.	.	.
olanzapine:aripiprazole	1	0.50	0.0885	-0.0262	0.2032	-0.2294	-0.60	0.5480
placebo:aripiprazole	2	0.53	-0.2040	-0.1421	-0.2748	0.1327	0.46	0.6427
quetiapine:aripiprazole	1	0.37	-0.1535	0.0200	-0.2552	0.2752	0.79	0.4276
risperidone/paliperidone:aripiprazole	1	0.44	0.2734	-0.0373	0.5138	-0.5511	-1.54	0.1244
ziprasidone:aripiprazole	0	0	-0.1461	.	-0.1461	.	.	.
molindone:olanzapine	1	0.85	0.1375	-0.0207	1.0004	-1.0211	-1.47	0.1408
molindone:placebo	0	0	0.4300	.	0.4300	.	.	.
molindone:quetiapine	0	0	0.3796	.	0.3796	.	.	.
molindone:risperidone/paliperidone	1	0.84	-0.0474	0.1114	-0.9080	1.0193	1.47	0.1408
molindone:ziprasidone	0	0	0.3721	.	0.3721	.	.	.
olanzapine:placebo	1	0.39	0.2925	-0.1010	0.5460	-0.6470	-1.80	0.0711
olanzapine:quetiapine	0	0	0.2421	.	0.2421	.	.	.
olanzapine:risperidone/paliperidone	2	0.76	-0.1849	0.0710	-0.9774	1.0485	2.70	0.0068
olanzapine:ziprasidone	0	0	0.2346	.	0.2346	.	.	.
placebo:quetiapine	4	0.85	-0.0505	-0.0082	-0.2834	0.2752	0.79	0.4276
placebo:risperidone/paliperidone	2	0.61	-0.4774	-0.8242	0.0562	-0.8804	-2.81	0.0050
placebo:ziprasidone	3	1.00	-0.0579	-0.0579	.	.	.	.
quetiapine:risperidone/paliperidone	0	0	-0.4269	.	-0.4269	.	.	.
quetiapine:ziprasidone	0	0	-0.0075	.	-0.0075	.	.	.
risperidone/paliperidone:ziprasidone	0	0	0.4195	.	0.4195	.	.	.

Legend:
comparison - Treatment comparison
k - Number of studies providing direct evidence
prop - Direct evidence proportion
nma - Estimated treatment effect (MD) in network meta-analysis
direct - Estimated treatment effect (MD) derived from direct evidence
indir. - Estimated treatment effect (MD) derived from indirect evidence
Diff - Difference between direct and indirect treatment estimates
z - z-value of test for disagreement (direct versus indirect)
p-value - p-value of test for disagreement (direct versus indirect)

eTable 33. 'Hot spots' of inconsistency: LDL cholesterol

Separate indirect from direct evidence (SIDE) using back-calculation method

Common effects model:

comparison	k	prop	nma	direct	indir.	Diff	z	p-value
lurasidone:aripiprazole	0	0	-0.0322	.	-0.0322	.	.	.
molindone:aripiprazole	0	0	-0.0021	.	-0.0021	.	.	.
olanzapine:aripiprazole	1	0.65	0.1912	0.0080	0.5276	-0.5196	-3.76	0.0002
placebo:aripiprazole	1	0.12	0.0478	0.1632	0.0322	0.1309	0.59	0.5574
quetiapine:aripiprazole	1	0.36	0.0970	0.0100	0.1454	-0.1354	-0.83	0.4038
risperidone/paliperidone:aripiprazole	2	0.87	-0.0527	-0.0067	-0.3480	0.3413	2.56	0.0105
ziprasidone:aripiprazole	0	0	0.0081	.	0.0081	.	.	.
lurasidone:molindone	0	0	-0.0301	.	-0.0301	.	.	.
lurasidone:olanzapine	0	0	-0.2234	.	-0.2234	.	.	.
lurasidone:placebo	1	1.00	-0.0800	-0.0800	.	.	.	.
lurasidone:quetiapine	0	0	-0.1292	.	-0.1292	.	.	.
lurasidone:risperidone/paliperidone	0	0	0.0204	.	0.0204	.	.	.
lurasidone:ziprasidone	0	0	-0.0403	.	-0.0403	.	.	.
molindone:olanzapine	1	0.79	-0.1933	-0.3683	0.4515	-0.8198	-3.75	0.0002
molindone:placebo	0	0	-0.0499	.	-0.0499	.	.	.
molindone:quetiapine	0	0	-0.0991	.	-0.0991	.	.	.
molindone:risperidone/paliperidone	1	0.73	0.0505	0.2611	-0.5110	0.7721	3.75	0.0002
molindone:ziprasidone	0	0	-0.0102	.	-0.0102	.	.	.
olanzapine:placebo	1	0.31	0.1434	0.1295	0.1496	-0.0201	-0.11	0.9102
olanzapine:quetiapine	0	0	0.0942	.	0.0942	.	.	.
olanzapine:risperidone/paliperidone	2	0.79	0.2439	0.3155	-0.0184	0.3339	2.12	0.0338
olanzapine:ziprasidone	0	0	0.1831	.	0.1831	.	.	.
placebo:quetiapine	2	0.91	-0.0492	-0.0608	0.0746	-0.1354	-0.83	0.4038
placebo:risperidone/paliperidone	2	0.53	0.1004	0.1178	0.0809	0.0369	0.26	0.7920
placebo:ziprasidone	3	1.00	0.0397	0.0397	.	.	.	.
quetiapine:risperidone/paliperidone	0	0	0.1497	.	0.1497	.	.	.
quetiapine:ziprasidone	0	0	0.0889	.	0.0889	.	.	.
risperidone/paliperidone:ziprasidone	0	0	-0.0607	.	-0.0607	.	.	.

Random effects model:

comparison	k	prop	nma	direct	indir.	Diff	z	p-value
lurasidone:aripiprazole	0	0	-0.0319	.	-0.0319	.	.	.
molindone:aripiprazole	0	0	0.0129	.	0.0129	.	.	.
olanzapine:aripiprazole	1	0.56	0.2179	0.0080	0.4835	-0.4755	-2.17	0.0302
placebo:aripiprazole	1	0.19	0.0481	0.1632	0.0216	0.1415	0.53	0.5974
quetiapine:aripiprazole	1	0.44	0.0802	0.0100	0.1348	-0.1248	-0.53	0.5973
risperidone/paliperidone:aripiprazole	2	0.77	-0.0689	-0.0111	-0.2640	0.2529	1.23	0.2186
ziprasidone:aripiprazole	0	0	0.0012	.	0.0012	.	.	.
lurasidone:molindone	0	0	-0.0449	.	-0.0449	.	.	.
lurasidone:olanzapine	0	0	-0.2498	.	-0.2498	.	.	.
lurasidone:placebo	1	1.00	-0.0800	-0.0800	.	.	.	.
lurasidone:quetiapine	0	0	-0.1122	.	-0.1122	.	.	.
lurasidone:risperidone/paliperidone	0	0	0.0370	.	0.0370	.	.	.
lurasidone:ziprasidone	0	0	-0.0332	.	-0.0332	.	.	.
molindone:olanzapine	1	0.82	-0.2050	-0.3683	0.5396	-0.9079	-2.45	0.0144
molindone:placebo	0	0	-0.0351	.	-0.0351	.	.	.
molindone:quetiapine	0	0	-0.0673	.	-0.0673	.	.	.
molindone:risperidone/paliperidone	1	0.79	0.0818	0.2611	-0.6117	0.8728	2.45	0.0144
molindone:ziprasidone	0	0	0.0117	.	0.0117	.	.	.
olanzapine:placebo	1	0.36	0.1698	0.1295	0.1928	-0.0633	-0.26	0.7914
olanzapine:quetiapine	0	0	0.1377	.	0.1377	.	.	.
olanzapine:risperidone/paliperidone	2	0.76	0.2868	0.3539	0.0783	0.2756	1.20	0.2298
olanzapine:ziprasidone	0	0	0.2167	.	0.2167	.	.	.
placebo:quetiapine	2	0.83	-0.0322	-0.0535	0.0713	-0.1248	-0.53	0.5973
placebo:risperidone/paliperidone	2	0.55	0.1170	0.1026	0.1347	-0.0321	-0.16	0.8696
placebo:ziprasidone	3	1.00	0.0468	0.0468	.	.	.	.
quetiapine:risperidone/paliperidone	0	0	0.1491	.	0.1491	.	.	.
quetiapine:ziprasidone	0	0	0.0790	.	0.0790	.	.	.
risperidone/paliperidone:ziprasidone	0	0	-0.0701	.	-0.0701	.	.	.

Legend:
comparison - Treatment comparison
k - Number of studies providing direct evidence
prop - Direct evidence proportion
nma - Estimated treatment effect (MD) in network meta-analysis
direct - Estimated treatment effect (MD) derived from direct evidence
indir. - Estimated treatment effect (MD) derived from indirect evidence
Diff - Difference between direct and indirect treatment estimates
z - z-value of test for disagreement (direct versus indirect)
p-value - p-value of test for disagreement (direct versus indirect)

eTable 34. 'Hot spots' of inconsistency: heart rate

Separate indirect from direct evidence (SIDE) using back-calculation method

Common effects model:

comparison	k	prop	nma	direct	indir.	Diff	z	p-value
haloperidol:aripiprazole	0	0	3.1888	.	3.1888	.	.	.
olanzapine:aripiprazole	0	0	-2.9511	.	-2.9511	.	.	.
placebo:aripiprazole	0	0	-3.6111	.	-3.6111	.	.	.
quetiapine:aripiprazole	1	0.81	9.8237	11.9000	0.9272	10.9728	1.82	0.0688
risperidone/paliperidone:aripiprazole	1	0.75	1.4888	-1.2000	9.7728	-10.9728	-1.82	0.0688
ziprasidone:aripiprazole	0	0	-3.8111	.	-3.8111	.	.	.
haloperidol:olanzapine	0	0	6.1399	.	6.1399	.	.	.
haloperidol:placebo	0	0	6.7999	.	6.7999	.	.	.
haloperidol:quetiapine	0	0	-6.6349	.	-6.6349	.	.	.
haloperidol:risperidone/paliperidone	1	1.00	1.7000	1.7000	.	.	.	.
haloperidol:ziprasidone	0	0	6.9999	.	6.9999	.	.	.
olanzapine:placebo	1	1.00	0.6600	0.6600	.	.	.	.
olanzapine:quetiapine	0	0	-12.7748	.	-12.7748	.	.	.
olanzapine:risperidone/paliperidone	0	0	-4.4399	.	-4.4399	.	.	.
olanzapine:ziprasidone	0	0	0.8600	.	0.8600	.	.	.
placebo:quetiapine	1	0.50	-13.4348	-8.0000	-18.9728	10.9728	1.82	0.0688
placebo:risperidone/paliperidone	2	0.93	-5.0999	-5.8728	5.1000	-10.9728	-1.82	0.0688
placebo:ziprasidone	1	1.00	0.2000	0.2000	.	.	.	.
quetiapine:risperidone/paliperidone	0	0	8.3349	.	8.3349	.	.	.
quetiapine:ziprasidone	0	0	13.6348	.	13.6348	.	.	.
risperidone/paliperidone:ziprasidone	0	0	5.2999	.	5.2999	.	.	.

Random effects model:

comparison	k	prop	nma	direct	indir.	Diff	z	p-value
haloperidol:aripiprazole	0	0	3.6190	.	3.6190	.	.	.
olanzapine:aripiprazole	0	0	-2.6914	.	-2.6914	.	.	.
placebo:aripiprazole	0	0	-3.3514	.	-3.3514	.	.	.
quetiapine:aripiprazole	1	0.76	9.0718	11.9000	0.1809	11.7191	1.30	0.1950
risperidone/paliperidone:aripiprazole	1	0.73	1.9190	-1.2000	10.5191	-11.7191	-1.30	0.1950
ziprasidone:aripiprazole	0	0	-3.5514	.	-3.5514	.	.	.
haloperidol:olanzapine	0	0	6.3104	.	6.3104	.	.	.
haloperidol:placebo	0	0	6.9704	.	6.9704	.	.	.
haloperidol:quetiapine	0	0	-5.4528	.	-5.4528	.	.	.
haloperidol:risperidone/paliperidone	1	1.00	1.7000	1.7000	.	.	.	.
haloperidol:ziprasidone	0	0	7.1704	.	7.1704	.	.	.
olanzapine:placebo	1	1.00	0.6600	0.6600	.	.	.	.
olanzapine:quetiapine	0	0	-11.7632	.	-11.7632	.	.	.
olanzapine:risperidone/paliperidone	0	0	-4.6104	.	-4.6104	.	.	.
olanzapine:ziprasidone	0	0	0.8600	.	0.8600	.	.	.
placebo:quetiapine	1	0.62	-12.4232	-8.0000	-19.7191	11.7191	1.30	0.1950
placebo:risperidone/paliperidone	2	0.88	-5.2704	-6.6191	5.1000	-11.7191	-1.30	0.1950
placebo:ziprasidone	1	1.00	0.2000	0.2000	.	.	.	.
quetiapine:risperidone/paliperidone	0	0	7.1528	.	7.1528	.	.	.
quetiapine:ziprasidone	0	0	12.6232	.	12.6232	.	.	.
risperidone/paliperidone:ziprasidone	0	0	5.4704	.	5.4704	.	.	.

Legend:
- comparison - Treatment comparison
- k - Number of studies providing direct evidence
- prop - Direct evidence proportion
- nma - Estimated treatment effect (MD) in network meta-analysis
- direct - Estimated treatment effect (MD) derived from direct evidence
- indir. - Estimated treatment effect (MD) derived from indirect evidence
- Diff - Difference between direct and indirect treatment estimates
- z - z-value of test for disagreement (direct versus indirect)
- p-value - p-value of test for disagreement (direct versus indirect)

eTable 35. 'Hot spots' of inconsistency: QTc interval

Separate indirect from direct evidence (SIDE) using back-calculation method

Common effects model:

comparison	k	prop	nma	direct	indir.	Diff	z	p-value
molindone:aripiprazole	0	0	-0.2195	.	-0.2195	.	.	.
olanzapine:aripiprazole	0	0	0.0974	.	0.0974	.	.	.
pimozide:aripiprazole	0	0	-0.5212	.	-0.5212	.	.	.
placebo:aripiprazole	1	0.94	0.0890	0.0754	0.3088	-0.2335	-0.46	0.6487
quetiapine:aripiprazole	1	0.86	0.3521	0.3841	0.1507	0.2335	0.46	0.6487
risperidone/paliperidone:aripiprazole	0	0	-0.1582	.	-0.1582	.	.	.

ziprasidone:aripiprazole	0	0	0.3851	.	0.3851	.	.	.
molindone:olanzapine	1	0.87	-0.3169	-0.4266	0.4470	-0.8736	-1.33	0.1843
molindone:pimozide	0	0	0.3017	.	0.3017	.	.	.
molindone:placebo	0	0	-0.3085	.	-0.3085	.	.	.
molindone:quetiapine	0	0	-0.5716	.	-0.5716	.	.	.
molindone:risperidone/paliperidone	1	0.90	-0.0613	0.0299	-0.9251	0.9550	1.33	0.1843
molindone:ziprasidone	0	0	-0.6046	.	-0.6046	.	.	.
olanzapine:pimozide	0	0	0.6186	.	0.6186	.	.	.
olanzapine:placebo	2	0.83	0.0084	-0.0707	0.4020	-0.4727	-1.33	0.1843
olanzapine:quetiapine	0	0	-0.2547	.	-0.2547	.	.	.
olanzapine:risperidone/paliperidone	1	0.58	0.2556	0.4564	-0.0163	0.4727	1.33	0.1843
olanzapine:ziprasidone	0	0	-0.2877	.	-0.2877	.	.	.
pimozide:placebo	0	0	-0.6102	.	-0.6102	.	.	.
pimozide:quetiapine	0	0	-0.8733	.	-0.8733	.	.	.
pimozide:risperidone/paliperidone	1	1.00	-0.3630	-0.3630	.	.	.	.
pimozide:ziprasidone	0	0	-0.9063	.	-0.9063	.	.	.
placebo:quetiapine	1	0.20	-0.2631	-0.0753	-0.3088	0.2335	0.46	0.6487
placebo:risperidone/paliperidone	2	0.59	0.2472	0.0544	0.5271	-0.4727	-1.33	0.1843
placebo:ziprasidone	1	1.00	-0.2961	-0.2961	.	.	.	.
quetiapine:risperidone/paliperidone	0	0	0.5103	.	0.5103	.	.	.
quetiapine:ziprasidone	0	0	-0.0330	.	-0.0330	.	.	.
risperidone/paliperidone:ziprasidone	0	0	-0.5433	.	-0.5433	.	.	.

Random effects model:

comparison	k	prop	nma	direct	indir.	Diff	z	p-value
molindone:aripiprazole	0	0	-0.2170	.	-0.2170	.	.	.
olanzapine:aripiprazole	0	0	0.0975	.	0.0975	.	.	.
pimozide:aripiprazole	0	0	-0.5152	.	-0.5152	.	.	.
placebo:aripiprazole	1	0.93	0.0925	0.0754	0.3088	-0.2335	-0.44	0.6578
quetiapine:aripiprazole	1	0.85	0.3496	0.3841	0.1507	0.2335	0.44	0.6578
risperidone/paliperidone:aripiprazole	0	0	-0.1522	.	-0.1522	.	.	.
ziprasidone:aripiprazole	0	0	0.3886	.	0.3886	.	.	.
molindone:olanzapine	1	0.88	-0.3145	-0.4266	0.4712	-0.8978	-1.30	0.1930
molindone:pimozide	0	0	0.2982	.	0.2982	.	.	.
molindone:placebo	0	0	-0.3095	.	-0.3095	.	.	.
molindone:quetiapine	0	0	-0.5666	.	-0.5666	.	.	.
molindone:risperidone/paliperidone	1	0.90	-0.0648	0.0299	-0.9443	0.9742	1.30	0.1930
molindone:ziprasidone	0	0	-0.6056	.	-0.6056	.	.	.
olanzapine:pimozide	0	0	0.6126	.	0.6126	.	.	.
olanzapine:placebo	2	0.82	0.0050	-0.0805	0.4021	-0.4826	-1.30	0.1930
olanzapine:quetiapine	0	0	-0.2521	.	-0.2521	.	.	.
olanzapine:risperidone/paliperidone	1	0.57	0.2496	0.4564	-0.0262	0.4826	1.30	0.1930
olanzapine:ziprasidone	0	0	-0.2911	.	-0.2911	.	.	.
pimozide:placebo	0	0	-0.6077	.	-0.6077	.	.	.
pimozide:quetiapine	0	0	-0.8648	.	-0.8648	.	.	.
pimozide:risperidone/paliperidone	1	1.00	-0.3630	-0.3630	.	.	.	.
pimozide:ziprasidone	0	0	-0.9038	.	-0.9038	.	.	.
placebo:quetiapine	1	0.22	-0.2571	-0.0753	-0.3088	0.2335	0.44	0.6578
placebo:risperidone/paliperidone	2	0.61	0.2446	0.0543	0.5369	-0.4826	-1.30	0.1930
placebo:ziprasidone	1	1.00	-0.2961	-0.2961	.	.	.	.
quetiapine:risperidone/paliperidone	0	0	0.5018	.	0.5018	.	.	.
quetiapine:ziprasidone	0	0	-0.0390	.	-0.0390	.	.	.
risperidone/paliperidone:ziprasidone	0	0	-0.5407	.	-0.5407	.	.	.

Legend:
comparison - Treatment comparison
k - Number of studies providing direct evidence
prop - Direct evidence proportion
nma - Estimated treatment effect (SMD) in network meta-analysis
direct - Estimated treatment effect (SMD) derived from direct evidence
indir. - Estimated treatment effect (SMD) derived from indirect evidence
Diff - Difference between direct and indirect treatment estimates
z - z-value of test for disagreement (direct versus indirect)
p-value - p-value of test for disagreement (direct versus indirect)

eTable 36. ‘Hot spots’ of inconsistency: systolic blood pressure

Separate indirect from direct evidence (SIDE) using back-calculation method

Common effects model:

comparison	k	prop	nma	direct	indir.	Diff	z	p-value
clozapine:aripiprazole	0	0	-9.1294	.	-9.1294	.	.	.
haloperidol:aripiprazole	0	0	-4.2594	.	-4.2594	.	.	.
olanzapine:aripiprazole	0	0	2.0320	.	2.0320	.	.	.
placebo:aripiprazole	1	0.47	-3.8480	-1.4500	-5.9620	4.5120	1.17	0.2434
quetiapine:aripiprazole	1	0.72	6.1883	5.0600	9.0900	-4.0300	-0.88	0.3776
risperidone/paliperidone:aripiprazole	1	0.62	0.2406	-0.5000	1.4279	-1.9279	-0.46	0.6475
ziprasidone:aripiprazole	0	0	-7.2480	.	-7.2480	.	.	.
clozapine:haloperidol	1	1.00	-4.8700	-4.8700	.	.	.	.
clozapine:olanzapine	0	0	-11.1614	.	-11.1614	.	.	.

clozapine:placebo	0	0	-5.2814	.	-5.2814	.	.	.
clozapine:quetiapine	0	0	-15.3177	.	-15.3177	.	.	.
clozapine:risperidone/paliperidone	0	0	-9.3700	.	-9.3700	.	.	.
clozapine:ziprasidone	0	0	-1.8814	.	-1.8814	.	.	.
haloperidol:olanzapine	0	0	-6.2914	.	-6.2914	.	.	.
haloperidol:placebo	0	0	-0.4114	.	-0.4114	.	.	.
haloperidol:quetiapine	0	0	-10.4477	.	-10.4477	.	.	.
haloperidol:risperidone/paliperidone	1	1.00	-4.5000	-4.5000	.	.	.	.
haloperidol:ziprasidone	0	0	2.9886	.	2.9886	.	.	.
olanzapine:placebo	1	1.00	5.8800	5.8800	.	.	.	.
olanzapine:quetiapine	0	0	-4.1563	.	-4.1563	.	.	.
olanzapine:risperidone/paliperidone	0	0	1.7914	.	1.7914	.	.	.
olanzapine:ziprasidone	0	0	9.2800	.	9.2800	.	.	.
placebo:quetiapine	1	0.51	-10.0363	-12.0000	-7.9700	-4.0300	-0.88	0.3776
placebo:risperidone/paliperidone	1	0.68	-4.0886	-4.7000	-2.7721	-1.9279	-0.46	0.6475
placebo:ziprasidone	1	1.00	3.4000	3.4000	.	.	.	.
quetiapine:risperidone/paliperidone	0	0	5.9477	.	5.9477	.	.	.
quetiapine:ziprasidone	0	0	13.4363	.	13.4363	.	.	.
risperidone/paliperidone:ziprasidone	0	0	7.4886	.	7.4886	.	.	.

Random effects model:

comparison	k	prop	nma	direct	indir.	Diff	z	p-value
clozapine:aripiprazole	0	0	-9.1294	.	-9.1294	.	.	.
haloperidol:aripiprazole	0	0	-4.2594	.	-4.2594	.	.	.
olanzapine:aripiprazole	0	0	2.0320	.	2.0320	.	.	.
placebo:aripiprazole	1	0.47	-3.8480	-1.4500	-5.9620	4.5120	1.17	0.2434
quetiapine:aripiprazole	1	0.72	6.1883	5.0600	9.0900	-4.0300	-0.88	0.3776
risperidone/paliperidone:aripiprazole	1	0.62	0.2406	-0.5000	1.4279	-1.9279	-0.46	0.6475
ziprasidone:aripiprazole	0	0	-7.2480	.	-7.2480	.	.	.
clozapine:haloperidol	1	1.00	-4.8700	-4.8700	.	.	.	.
clozapine:olanzapine	0	0	-11.1614	.	-11.1614	.	.	.
clozapine:placebo	0	0	-5.2814	.	-5.2814	.	.	.
clozapine:quetiapine	0	0	-15.3177	.	-15.3177	.	.	.
clozapine:risperidone/paliperidone	0	0	-9.3700	.	-9.3700	.	.	.
clozapine:ziprasidone	0	0	-1.8814	.	-1.8814	.	.	.
haloperidol:olanzapine	0	0	-6.2914	.	-6.2914	.	.	.
haloperidol:placebo	0	0	-0.4114	.	-0.4114	.	.	.
haloperidol:quetiapine	0	0	-10.4477	.	-10.4477	.	.	.
haloperidol:risperidone/paliperidone	1	1.00	-4.5000	-4.5000	.	.	.	.
haloperidol:ziprasidone	0	0	2.9886	.	2.9886	.	.	.
olanzapine:placebo	1	1.00	5.8800	5.8800	.	.	.	.
olanzapine:quetiapine	0	0	-4.1563	.	-4.1563	.	.	.
olanzapine:risperidone/paliperidone	0	0	1.7914	.	1.7914	.	.	.
olanzapine:ziprasidone	0	0	9.2800	.	9.2800	.	.	.
placebo:quetiapine	1	0.51	-10.0363	-12.0000	-7.9700	-4.0300	-0.88	0.3776
placebo:risperidone/paliperidone	1	0.68	-4.0886	-4.7000	-2.7721	-1.9279	-0.46	0.6475
placebo:ziprasidone	1	1.00	3.4000	3.4000	.	.	.	.
quetiapine:risperidone/paliperidone	0	0	5.9477	.	5.9477	.	.	.
quetiapine:ziprasidone	0	0	13.4363	.	13.4363	.	.	.
risperidone/paliperidone:ziprasidone	0	0	7.4886	.	7.4886	.	.	.

Legend:
- comparison - Treatment comparison
- k - Number of studies providing direct evidence
- prop - Direct evidence proportion
- nma - Estimated treatment effect (MD) in network meta-analysis
- direct - Estimated treatment effect (MD) derived from direct evidence
- indir. - Estimated treatment effect (MD) derived from indirect evidence
- Diff - Difference between direct and indirect treatment estimates
- z - z-value of test for disagreement (direct versus indirect)
- p-value - p-value of test for disagreement (direct versus indirect)

eTable 37. CINeMA confidence rating for weight

Comparison	Number of studies	Within-study bias	Reporting bias	Indirectness	Imprecision	Heterogeneity	Incoherence	Confidence rating
**aripiprazole:olanzapine**	1	Some concerns	Low risk	No concerns	No concerns	No concerns	No concerns	Moderate
**aripiprazole:placebo**	7	Some concerns	Low risk	No concerns	No concerns	No concerns	No concerns	Moderate
**aripiprazole:quetiapine**	1	Some concerns	Low risk	No concerns	No concerns	No concerns	No concerns	Moderate
**aripiprazole:risperidone_paliperidone**	4	Some concerns	Low risk	No concerns	No concerns	No concerns	No concerns	Moderate
**asenapine:placebo**	1	No concerns	Low risk	No concerns	No concerns	No concerns	No concerns	High
**blonanserin:placebo**	1	No concerns	Low risk	No concerns	No concerns	No concerns	No concerns	High
**clozapine:haloperidol**	1	Some concerns	Low risk	No concerns	Major concerns	No concerns	No concerns	Very low
**clozapine:olanzapine**	1	Some concerns	Low risk	No concerns	Major concerns	No concerns	No concerns	Very low
**haloperidol:risperidone_paliperidone**	1	Some concerns	Low risk	No concerns	Some concerns	No concerns	No concerns	Moderate
**lurasidone:placebo**	3	No concerns	Low risk	No concerns	No concerns	No concerns	No concerns	High
**molindone:olanzapine**	1	Some concerns	Low risk	No concerns	No concerns	No concerns	No concerns	Moderate
**molindone:risperidone_paliperidone**	1	Some concerns	Low risk	No concerns	No concerns	No concerns	No concerns	Moderate
**olanzapine:placebo**	2	Some concerns	Low risk	No concerns	No concerns	No concerns	No concerns	Moderate
**olanzapine:risperidone_paliperidone**	2	Some concerns	Low risk	No concerns	No concerns	No concerns	No concerns	Moderate
**pimozide:risperidone_paliperidone**	1	Some concerns	Low risk	No concerns	Major concerns	No concerns	No concerns	Very low
**placebo:quetiapine**	4	Some concerns	Low risk	No concerns	No concerns	No concerns	No concerns	Moderate

**placebo:risperidone_paliperidone**	9	Some concerns	Low risk	No concerns	No concerns	No concerns	No concerns	Moderate
**placebo:ziprasidone**	4	Some concerns	Low risk	No concerns	No concerns	No concerns	No concerns	Moderate
**aripiprazole:asenapine**	0	Some concerns	Low risk	No concerns	No concerns	No concerns	No concerns	Moderate
**aripiprazole:blonanserin**	0	Some concerns	Low risk	No concerns	No concerns	No concerns	No concerns	Moderate
**aripiprazole:clozapine**	0	Some concerns	Low risk	No concerns	Some concerns	No concerns	No concerns	Moderate
**aripiprazole:haloperidol**	0	Some concerns	Low risk	No concerns	Some concerns	No concerns	No concerns	Moderate
**aripiprazole:lurasidone**	0	Some concerns	Low risk	No concerns	No concerns	No concerns	No concerns	Moderate
**aripiprazole:molindone**	0	Some concerns	Low risk	No concerns	No concerns	No concerns	No concerns	Moderate
**aripiprazole:pimozide**	0	Some concerns	Low risk	No concerns	Major concerns	No concerns	No concerns	Very low
**aripiprazole:ziprasidone**	0	Some concerns	Low risk	No concerns	No concerns	No concerns	No concerns	Moderate
**asenapine:blonanserin**	0	No concerns	Low risk	No concerns	No concerns	Some concerns	No concerns	Moderate
**asenapine:clozapine**	0	Some concerns	Low risk	No concerns	Some concerns	No concerns	No concerns	Moderate
**asenapine:haloperidol**	0	Some concerns	Low risk	No concerns	Some concerns	No concerns	No concerns	Moderate
**asenapine:lurasidone**	0	No concerns	Low risk	No concerns	No concerns	No concerns	No concerns	High
**asenapine:molindone**	0	Some concerns	Low risk	No concerns	No concerns	No concerns	No concerns	Moderate
**asenapine:olanzapine**	0	No concerns	Low risk	No concerns	No concerns	No concerns	No concerns	High
**asenapine:pimozide**	0	Some concerns	Low risk	No concerns	Major concerns	No concerns	No concerns	Very low
**asenapine:quetiapine**	0	No concerns	Low risk	No concerns	No concerns	No concerns	No concerns	High
**asenapine:risperidone_paliperidone**	0	No concerns	Low risk	No concerns	No concerns	No concerns	No concerns	High

**asenapine:ziprasidone**	0	No concerns	Low risk	No concerns	No concerns	Some concerns	No concerns	Moderate
**blonanserin:clozapine**	0	Some concerns	Low risk	No concerns	No concerns	No concerns	No concerns	Moderate
**blonanserin:haloperidol**	0	Some concerns	Low risk	No concerns	No concerns	Some concerns	No concerns	Moderate
**blonanserin:lurasidone**	0	No concerns	Low risk	No concerns	No concerns	No concerns	No concerns	High
**blonanserin:molindone**	0	Some concerns	Low risk	No concerns	No concerns	Some concerns	No concerns	Moderate
**blonanserin:olanzapine**	0	No concerns	Low risk	No concerns	No concerns	No concerns	No concerns	High
**blonanserin:pimozide**	0	Some concerns	Low risk	No concerns	Major concerns	No concerns	No concerns	Very low
**blonanserin:quetiapine**	0	No concerns	Low risk	No concerns	No concerns	Some concerns	No concerns	Moderate
**blonanserin:risperidone_paliperidone**	0	No concerns	Low risk	No concerns	No concerns	Some concerns	No concerns	Moderate
**blonanserin:ziprasidone**	0	No concerns	Low risk	No concerns	No concerns	No concerns	No concerns	High
**clozapine:lurasidone**	0	Some concerns	Low risk	No concerns	No concerns	No concerns	No concerns	Moderate
**clozapine:molindone**	0	Some concerns	Low risk	No concerns	No concerns	No concerns	No concerns	Moderate
**clozapine:pimozide**	0	Some concerns	Low risk	No concerns	Major concerns	No concerns	No concerns	Very low
**clozapine:placebo**	0	Some concerns	Low risk	No concerns	No concerns	No concerns	No concerns	Moderate
**clozapine:quetiapine**	0	Some concerns	Low risk	No concerns	Some concerns	No concerns	No concerns	Moderate
**clozapine:risperidone_paliperidone**	0	Some concerns	Low risk	No concerns	Some concerns	No concerns	No concerns	Moderate
**clozapine:ziprasidone**	0	Some concerns	Low risk	No concerns	No concerns	No concerns	No concerns	Moderate
**haloperidol:lurasidone**	0	Some concerns	Low risk	No concerns	Some concerns	No concerns	No concerns	Moderate
**haloperidol:molindone**	0	Some concerns	Low risk	No concerns	No concerns	No concerns	No concerns	Moderate

**haloperidol:olanzapine**	0	Some concerns	Low risk	No concerns	Major concerns	No concerns	No concerns	Very low
**haloperidol:pimozide**	0	Some concerns	Low risk	No concerns	Major concerns	No concerns	No concerns	Very low
**haloperidol:placebo**	0	Some concerns	Low risk	No concerns	No concerns	Some concerns	No concerns	Moderate
**haloperidol:quetiapine**	0	Some concerns	Low risk	No concerns	Some concerns	No concerns	No concerns	Moderate
**haloperidol:ziprasidone**	0	Some concerns	Low risk	No concerns	No concerns	No concerns	No concerns	Moderate
**lurasidone:molindone**	0	Some concerns	Low risk	No concerns	No concerns	No concerns	No concerns	Moderate
**lurasidone:olanzapine**	0	No concerns	Low risk	No concerns	No concerns	No concerns	No concerns	High
**lurasidone:pimozide**	0	Some concerns	Low risk	No concerns	Major concerns	No concerns	No concerns	Very low
**lurasidone:quetiapine**	0	No concerns	Low risk	No concerns	No concerns	No concerns	No concerns	High
**lurasidone:risperidone_paliperidone**	0	No concerns	Low risk	No concerns	No concerns	No concerns	No concerns	High
**lurasidone:ziprasidone**	0	No concerns	Low risk	No concerns	No concerns	No concerns	No concerns	High
**molindone:pimozide**	0	Some concerns	Low risk	No concerns	Major concerns	No concerns	No concerns	Very low
**molindone:placebo**	0	Some concerns	Low risk	No concerns	No concerns	No concerns	No concerns	Moderate
**molindone:quetiapine**	0	Some concerns	Low risk	No concerns	No concerns	No concerns	No concerns	Moderate
**molindone:ziprasidone**	0	Some concerns	Low risk	No concerns	No concerns	Some concerns	No concerns	Moderate
**olanzapine:pimozide**	0	Some concerns	Low risk	No concerns	Major concerns	No concerns	No concerns	Very low
**olanzapine:quetiapine**	0	Some concerns	Low risk	No concerns	No concerns	No concerns	No concerns	Moderate
**olanzapine:ziprasidone**	0	Some concerns	Low risk	No concerns	No concerns	No concerns	No concerns	Moderate
**pimozide:placebo**	0	Some concerns	Low risk	No concerns	Major concerns	No concerns	No concerns	Very low

**pimozide:quetiapine**	0	Some concerns	Low risk	No concerns	Major concerns	No concerns	No concerns	Very low
**pimozide:ziprasidone**	0	Some concerns	Low risk	No concerns	Major concerns	No concerns	No concerns	Very low
**quetiapine:risperidone_paliperidone**	0	Some concerns	Low risk	No concerns	No concerns	No concerns	No concerns	Moderate
**quetiapine:ziprasidone**	0	Some concerns	Low risk	No concerns	No concerns	Some concerns	No concerns	Moderate
**risperidone_paliperidone:ziprasidone**	0	Some concerns	Low risk	No concerns	No concerns	Some concerns	No concerns	Moderate

eTable 38. CINeMA confidence rating for BMI

Comparison	Number of studies	Within-study bias	Reporting bias	Indirectness	Imprecision	Heterogeneity	Incoherence	Confidence rating
**aripiprazole:placebo**	6	Some concerns	Low risk	No concerns	No concerns	No concerns	No concerns	Moderate
**aripiprazole:quetiapine**	1	Some concerns	Low risk	No concerns	No concerns	No concerns	Some concerns	Moderate
**aripiprazole:risperidone/paliperidone**	1	Some concerns	Low risk	No concerns	No concerns	No concerns	No concerns	Moderate
**asenapine:placebo**	1	No concerns	Low risk	No concerns	No concerns	No concerns	No concerns	High
**clozapine:olanzapine**	2	No concerns	Low risk	No concerns	Major concerns	No concerns	No concerns	Very low
**lurasidone:placebo**	2	No concerns	Low risk	No concerns	No concerns	No concerns	No concerns	High
**molindone:olanzapine**	1	Some concerns	Low risk	No concerns	No concerns	No concerns	No concerns	Moderate
**molindone:risperidone/paliperidone**	1	Some concerns	Low risk	No concerns	No concerns	No concerns	No concerns	Moderate
**olanzapine:placebo**	2	Some concerns	Low risk	No concerns	No concerns	No concerns	No concerns	Moderate

**olanzapine:risperidone/paliperidone**	1	Some concerns	Low risk	No concerns	No concerns	No concerns	No concerns	Moderate
**placebo:quetiapine**	1	Some concerns	Low risk	No concerns	No concerns	No concerns	Some concerns	Moderate
**placebo:risperidone/paliperidone**	3	Some concerns	Low risk	No concerns	No concerns	No concerns	No concerns	Moderate
**placebo:ziprasidone**	1	Some concerns	Low risk	No concerns	No concerns	No concerns	No concerns	Moderate
**aripiprazole:asenapine**	0	Some concerns	Low risk	No concerns	No concerns	No concerns	No concerns	Moderate
**aripiprazole:clozapine**	0	Some concerns	Low risk	No concerns	Some concerns	No concerns	No concerns	Moderate
**aripiprazole:lurasidone**	0	Some concerns	Low risk	No concerns	No concerns	No concerns	No concerns	Moderate
**aripiprazole:molindone**	0	Some concerns	Low risk	No concerns	No concerns	No concerns	No concerns	Moderate
**aripiprazole:olanzapine**	0	Some concerns	Low risk	No concerns	No concerns	No concerns	No concerns	Moderate
**aripiprazole:ziprasidone**	0	Some concerns	Low risk	No concerns	No concerns	No concerns	No concerns	Moderate
**asenapine:clozapine**	0	No concerns	Low risk	No concerns	Some concerns	No concerns	No concerns	Moderate
**asenapine:lurasidone**	0	No concerns	Low risk	No concerns	No concerns	No concerns	No concerns	High
**asenapine:molindone**	0	Some concerns	Low risk	No concerns	No concerns	No concerns	No concerns	Moderate
**asenapine:olanzapine**	0	Some concerns	Low risk	No concerns	No concerns	Some concerns	No concerns	Moderate
**asenapine:quetiapine**	0	No concerns	Low risk	No concerns	No concerns	No concerns	No concerns	High
**asenapine:risperidone/paliperidone**	0	Some concerns	Low risk	No concerns	No concerns	No concerns	No concerns	Moderate
**asenapine:ziprasidone**	0	No concerns	Low risk	No concerns	No concerns	No concerns	No concerns	High
**clozapine:lurasidone**	0	No concerns	Low risk	No concerns	Some concerns	No concerns	No concerns	Moderate

**clozapine:molindone**	0	Some concerns	Low risk	No concerns	No concerns	No concerns	No concerns	Moderate
**clozapine:placebo**	0	Some concerns	Low risk	No concerns	Some concerns	No concerns	No concerns	Moderate
**clozapine:quetiapine**	0	Some concerns	Low risk	No concerns	Some concerns	No concerns	No concerns	Moderate
**clozapine:risperidone/paliperidone**	0	Some concerns	Low risk	No concerns	Some concerns	No concerns	No concerns	Moderate
**clozapine:ziprasidone**	0	Some concerns	Low risk	No concerns	Some concerns	No concerns	No concerns	Moderate
**lurasidone:molindone**	0	Some concerns	Low risk	No concerns	No concerns	No concerns	No concerns	Moderate
**lurasidone:olanzapine**	0	Some concerns	Low risk	No concerns	No concerns	No concerns	No concerns	Moderate
**lurasidone:quetiapine**	0	No concerns	Low risk	No concerns	No concerns	No concerns	No concerns	High
**lurasidone:risperidone/paliperidone**	0	Some concerns	Low risk	No concerns	No concerns	No concerns	No concerns	Moderate
**lurasidone:ziprasidone**	0	No concerns	Low risk	No concerns	No concerns	No concerns	No concerns	High
**molindone:placebo**	0	Some concerns	Low risk	No concerns	No concerns	No concerns	No concerns	Moderate
**molindone:quetiapine**	0	Some concerns	Low risk	No concerns	No concerns	No concerns	No concerns	Moderate
**molindone:ziprasidone**	0	Some concerns	Low risk	No concerns	No concerns	Some concerns	No concerns	Moderate
**olanzapine:quetiapine**	0	Some concerns	Low risk	No concerns	No concerns	No concerns	No concerns	Moderate
**olanzapine:ziprasidone**	0	Some concerns	Low risk	No concerns	No concerns	No concerns	No concerns	Moderate
**quetiapine:risperidone/paliperidone**	0	Some concerns	Low risk	No concerns	No concerns	No concerns	No concerns	Moderate
**quetiapine:ziprasidone**	0	Some concerns	Low risk	No concerns	No concerns	No concerns	No concerns	Moderate
**risperidone/paliperidone:ziprasidone**	0	Some concerns	Low risk	No concerns	No concerns	Some concerns	No concerns	Moderate

eTable 39. CINeMA confidence rating for triglycerides

Comparison	Number of studies	Within-study bias	Reporting bias	Indirectness	Imprecision	Heterogeneity	Incoherence	Confidence rating
**aripiprazole:olanzapine**	1	Major concerns	Low risk	No concerns	Some concerns	No concerns	No concerns	Very low
**aripiprazole:placebo**	4	Some concerns	Low risk	No concerns	No concerns	No concerns	Some concerns	Moderate
**aripiprazole:quetiapine**	1	No concerns	Low risk	No concerns	Some concerns	No concerns	Some concerns	Moderate
**aripiprazole:risperidone/paliperidone**	2	Some concerns	Low risk	No concerns	Some concerns	Some concerns	No concerns	Low
**asenapine:placebo**	2	No concerns	Low risk	No concerns	No concerns	No concerns	Some concerns	Moderate
**blonanserin:placebo**	1	No concerns	Low risk	No concerns	Some concerns	No concerns	Some concerns	Moderate
**clozapine:olanzapine**	1	Some concerns	Low risk	No concerns	Major concerns	No concerns	Some concerns	Very low
**lurasidone:placebo**	2	No concerns	Low risk	No concerns	Some concerns	Some concerns	Some concerns	Low
**molindone:olanzapine**	1	Some concerns	Low risk	No concerns	No concerns	Some concerns	No concerns	High
**molindone:risperidone/paliperidone**	1	Some concerns	Low risk	No concerns	Some concerns	No concerns	No concerns	Moderate
**olanzapine:placebo**	1	Some concerns	Low risk	No concerns	No concerns	No concerns	No concerns	Moderate
**olanzapine:risperidone/paliperidone**	2	Some concerns	Low risk	No concerns	Some concerns	No concerns	No concerns	Moderate
**placebo:quetiapine**	4	No concerns	Low risk	No concerns	No concerns	No concerns	Some concerns	Moderate
**placebo:risperidone/paliperidone**	4	Some concerns	Low risk	No concerns	No concerns	No concerns	No concerns	Moderate
**placebo:ziprasidone**	3	Some concerns	Low risk	No concerns	Major concerns	No concerns	Some concerns	Very low

**aripiprazole:asenapine**	0	No concerns	Low risk	No concerns	Major concerns	No concerns	Some concerns	Very low
**aripiprazole:blonanserin**	0	No concerns	Low risk	No concerns	Major concerns	No concerns	Some concerns	Very low
**aripiprazole:clozapine**	0	Some concerns	Low risk	No concerns	Major concerns	No concerns	Some concerns	Very low
**aripiprazole:lurasidone**	0	No concerns	Low risk	No concerns	No concerns	No concerns	Some concerns	Moderate
**aripiprazole:molindone**	0	Some concerns	Low risk	No concerns	Some concerns	Some concerns	Some concerns	Low
**aripiprazole:ziprasidone**	0	Some concerns	Low risk	No concerns	No concerns	No concerns	Some concerns	Low
**asenapine:blonanserin**	0	No concerns	Low risk	No concerns	Major concerns	No concerns	Some concerns	Very low
**asenapine:clozapine**	0	Some concerns	Low risk	No concerns	Major concerns	No concerns	Some concerns	Very low
**asenapine:lurasidone**	0	No concerns	Low risk	No concerns	No concerns	No concerns	Some concerns	Moderate
**asenapine:molindone**	0	Some concerns	Low risk	No concerns	Major concerns	No concerns	Some concerns	Very low
**asenapine:olanzapine**	0	No concerns	Low risk	No concerns	Some concerns	Some concerns	Some concerns	Low
**asenapine:quetiapine**	0	No concerns	Low risk	No concerns	Major concerns	No concerns	Some concerns	Very low
**asenapine:risperidone/paliperidone**	0	No concerns	Low risk	No concerns	Major concerns	No concerns	Some concerns	Very low
**asenapine:ziprasidone**	0	No concerns	Low risk	No concerns	No concerns	No concerns	Some concerns	Moderate
**blonanserin:clozapine**	0	Some concerns	Low risk	No concerns	Major concerns	No concerns	Some concerns	Very low
**blonanserin:lurasidone**	0	No concerns	Low risk	No concerns	Some concerns	No concerns	Some concerns	Moderate
**blonanserin:molindone**	0	Some concerns	Low risk	No concerns	Major concerns	No concerns	Some concerns	Very low
**blonanserin:olanzapine**	0	No concerns	Low risk	No concerns	Some concerns	Some concerns	Some concerns	Low

**blonanserin:quetiapine**	0	No concerns	Low risk	No concerns	Major concerns	No concerns	Some concerns	Very low
**blonanserin:risperidone/paliperidone**	0	No concerns	Low risk	No concerns	Major concerns	No concerns	Some concerns	Very low
**blonanserin:ziprasidone**	0	No concerns	Low risk	No concerns	Some concerns	No concerns	Some concerns	Moderate
**clozapine:lurasidone**	0	Some concerns	Low risk	No concerns	Some concerns	No concerns	Some concerns	Low
**clozapine:molindone**	0	Some concerns	Low risk	No concerns	Some concerns	Some concerns	Some concerns	Low
**clozapine:placebo**	0	Some concerns	Low risk	No concerns	Some concerns	No concerns	Some concerns	Low
**clozapine:quetiapine**	0	Some concerns	Low risk	No concerns	Major concerns	No concerns	Some concerns	Very low
**clozapine:risperidone/paliperidone**	0	Some concerns	Low risk	No concerns	Major concerns	No concerns	Some concerns	Very low
**clozapine:ziprasidone**	0	Some concerns	Low risk	No concerns	Some concerns	No concerns	Some concerns	Low
**lurasidone:molindone**	0	Some concerns	Low risk	No concerns	Major concerns	No concerns	Some concerns	Very low
**lurasidone:olanzapine**	0	No concerns	Low risk	No concerns	No concerns	No concerns	Some concerns	Moderate
**lurasidone:quetiapine**	0	No concerns	Low risk	No concerns	No concerns	No concerns	Some concerns	Moderate
**lurasidone:risperidone/paliperidone**	0	No concerns	Low risk	No concerns	No concerns	No concerns	Some concerns	Moderate
**lurasidone:ziprasidone**	0	No concerns	Low risk	No concerns	Major concerns	No concerns	Some concerns	Very low
**molindone:placebo**	0	Some concerns	Low risk	No concerns	Major concerns	No concerns	Some concerns	Very low
**molindone:quetiapine**	0	Some concerns	Low risk	No concerns	Some concerns	No concerns	Some concerns	Low
**molindone:ziprasidone**	0	Some concerns	Low risk	No concerns	Major concerns	No concerns	Some concerns	Very low
**olanzapine:quetiapine**	0	No concerns	Low risk	No concerns	Some concerns	Some concerns	Some concerns	Low

**olanzapine:ziprasidone**	0	Some concerns	Low risk	No concerns	No concerns	No concerns	Some concerns	Moderate
**quetiapine:risperidone/paliperidone**	0	No concerns	Low risk	No concerns	Major concerns	No concerns	Some concerns	Very low
**quetiapine:ziprasidone**	0	Some concerns	Low risk	No concerns	No concerns	No concerns	Some concerns	Low
**risperidone/paliperidone:ziprasidone**	0	Some concerns	Low risk	No concerns	No concerns	No concerns	Some concerns	Low

eTable 40. CINeMA confidence rating for prolactin

Comparison	Number of studies	Within-study bias	Reporting bias	Indirectness	Imprecision	Heterogeneity	Incoherence	Confidence rating
**aripiprazole:placebo**	6	Some concerns	Low risk	No concerns	Major concerns	No concerns	Some concerns	Very low
**aripiprazole:quetiapine**	1	No concerns	Low risk	No concerns	Major concerns	No concerns	No concerns	Very low
**aripiprazole:risperidone_paliperidone**	2	Some concerns	Low risk	No concerns	No concerns	No concerns	No concerns	Moderate
**asenapine:placebo**	2	No concerns	Low risk	No concerns	Major concerns	No concerns	No concerns	Very low
**blonanserin:placebo**	1	No concerns	Low risk	No concerns	Major concerns	No concerns	No concerns	Very low
**lurasidone:placebo**	3	No concerns	Low risk	No concerns	Major concerns	No concerns	No concerns	Very low
**molindone:olanzapine**	1	Some concerns	Low risk	No concerns	Major concerns	No concerns	No concerns	Very low
**molindone:risperidone_paliperidone**	1	Some concerns	Low risk	No concerns	No concerns	No concerns	No concerns	Moderate
**olanzapine:placebo**	2	Some concerns	Low risk	No concerns	No concerns	Major concerns	No concerns	Very low
**olanzapine:risperidone_paliperidone**	1	Some concerns	Low risk	No concerns	No concerns	Major concerns	No concerns	Very low

**placebo:quetiapine**	4	No concerns	Low risk	No concerns	Major concerns	No concerns	No concerns	Very low
**placebo:risperidone_paliperidone**	8	Some concerns	Low risk	No concerns	No concerns	No concerns	No concerns	Moderate
**aripiprazole:asenapine**	0	No concerns	Low risk	No concerns	Major concerns	No concerns	No concerns	Very low
**aripiprazole:blonanserin**	0	No concerns	Low risk	No concerns	Major concerns	No concerns	No concerns	Very low
**aripiprazole:lurasidone**	0	No concerns	Low risk	No concerns	Major concerns	No concerns	No concerns	Very low
**aripiprazole:molindone**	0	Some concerns	Low risk	No concerns	Major concerns	No concerns	No concerns	Very low
**aripiprazole:olanzapine**	0	Some concerns	Low risk	No concerns	No concerns	Major concerns	No concerns	Very low
**asenapine:blonanserin**	0	No concerns	Low risk	No concerns	Major concerns	No concerns	No concerns	Very low
**asenapine:lurasidone**	0	No concerns	Low risk	No concerns	Major concerns	No concerns	No concerns	Very low
**asenapine:molindone**	0	Some concerns	Low risk	No concerns	Major concerns	No concerns	No concerns	Very low
**asenapine:olanzapine**	0	No concerns	Low risk	No concerns	Major concerns	No concerns	No concerns	Very low
**asenapine:quetiapine**	0	No concerns	Low risk	No concerns	Major concerns	No concerns	No concerns	Very low
**asenapine:risperidone_paliperidone**	0	No concerns	Low risk	No concerns	No concerns	No concerns	No concerns	High
**blonanserin:lurasidone**	0	No concerns	Low risk	No concerns	Major concerns	No concerns	No concerns	Very low
**blonanserin:molindone**	0	Some concerns	Low risk	No concerns	Major concerns	No concerns	No concerns	Very low
**blonanserin:olanzapine**	0	No concerns	Low risk	No concerns	Major concerns	No concerns	No concerns	Very low
**blonanserin:quetiapine**	0	No concerns	Low risk	No concerns	Major concerns	No concerns	No concerns	Very low
**blonanserin:risperidone_paliperidone**	0	No concerns	Low risk	No concerns	Major concerns	No concerns	No concerns	Very low

**lurasidone:molindone**	0	Some concerns	Low risk	No concerns	Major concerns	No concerns	No concerns	Very low
**lurasidone:olanzapine**	0	No concerns	Low risk	No concerns	Major concerns	No concerns	No concerns	Very low
**lurasidone:quetiapine**	0	No concerns	Low risk	No concerns	Major concerns	No concerns	No concerns	Very low
**lurasidone:risperidone_paliperidone**	0	No concerns	Low risk	No concerns	No concerns	No concerns	No concerns	High
**molindone:placebo**	0	Some concerns	Low risk	No concerns	Major concerns	No concerns	No concerns	Very low
**molindone:quetiapine**	0	Some concerns	Low risk	No concerns	Major concerns	No concerns	No concerns	Very low
**olanzapine:quetiapine**	0	No concerns	Low risk	No concerns	Major concerns	No concerns	No concerns	Very low
**quetiapine:risperidone_paliperidone**	0	Some concerns	Low risk	No concerns	No concerns	No concerns	No concerns	Moderate

eTable 41. CINeMA confidence rating for glucose

Comparison	Number of studies	Within-study bias	Reporting bias	Indirectness	Imprecision	Heterogeneity	Incoherence	Confidence rating
**aripiprazole:olanzapine**	1	Major concerns	Low risk	No concerns	Major concerns	No concerns	No concerns	Very low
**aripiprazole:placebo**	4	Some concerns	Low risk	No concerns	Major concerns	No concerns	No concerns	Very low
**aripiprazole:quetiapine**	1	No concerns	Low risk	No concerns	Major concerns	No concerns	No concerns	Very low
**aripiprazole:risperidone/paliperidone**	3	Some concerns	Low risk	No concerns	Major concerns	No concerns	No concerns	Very low
**asenapine:placebo**	2	No concerns	Low risk	No concerns	Major concerns	No concerns	No concerns	Very low
**blonanserin:placebo**	1	No concerns	Low risk	No concerns	Major concerns	No concerns	No concerns	High
**clozapine:olanzapine**	1	Some concerns	Low risk	No concerns	Major concerns	No concerns	No concerns	Very low

**lurasidone:placebo**	2	No concerns	Low risk	No concerns	Major concerns	No concerns	No concerns	Very low
**molindone:olanzapine**	1	Some concerns	Low risk	No concerns	Major concerns	No concerns	No concerns	Very low
**molindone:risperidone/paliperidone**	1	Some concerns	Low risk	No concerns	Major concerns	No concerns	No concerns	Very low
**olanzapine:placebo**	2	Some concerns	Low risk	No concerns	Major concerns	No concerns	No concerns	Very low
**olanzapine:risperidone/paliperidone**	2	Some concerns	Low risk	No concerns	Major concerns	No concerns	No concerns	Very low
**placebo:quetiapine**	3	No concerns	Low risk	No concerns	No concerns	Major concerns	No concerns	Very low
**placebo:risperidone/paliperidone**	2	Some concerns	Low risk	No concerns	Major concerns	No concerns	No concerns	Very low
**placebo:ziprasidone**	3	Some concerns	Low risk	No concerns	Major concerns	No concerns	No concerns	Very low
**aripiprazole:asenapine**	0	No concerns	Low risk	No concerns	Major concerns	No concerns	No concerns	Very low
**aripiprazole:blonanserin**	0	No concerns	Low risk	No concerns	Major concerns	No concerns	No concerns	Very low
**aripiprazole:clozapine**	0	Some concerns	Low risk	No concerns	Major concerns	No concerns	No concerns	Very low
**aripiprazole:lurasidone**	0	No concerns	Low risk	No concerns	Major concerns	No concerns	No concerns	Very low
**aripiprazole:molindone**	0	Some concerns	Low risk	No concerns	Major concerns	No concerns	No concerns	Very low
**aripiprazole:ziprasidone**	0	Some concerns	Low risk	No concerns	Major concerns	No concerns	No concerns	Very low
**asenapine:blonanserin**	0	No concerns	Low risk	No concerns	Major concerns	No concerns	No concerns	Very low
**asenapine:clozapine**	0	Some concerns	Low risk	No concerns	Major concerns	No concerns	No concerns	Very low
**asenapine:lurasidone**	0	No concerns	Low risk	No concerns	Major concerns	No concerns	No concerns	Very low

**asenapine:molindone**	0	Some concerns	Low risk	No concerns	Major concerns	No concerns	No concerns	Very low
**asenapine:olanzapine**	0	No concerns	Low risk	No concerns	Major concerns	No concerns	No concerns	Very low
**asenapine:quetiapine**	0	No concerns	Low risk	No concerns	Major concerns	No concerns	No concerns	Very low
**asenapine:risperidone/paliperidone**	0	No concerns	Low risk	No concerns	Major concerns	No concerns	No concerns	Very low
**asenapine:ziprasidone**	0	No concerns	Low risk	No concerns	Major concerns	No concerns	No concerns	Very low
**blonanserin:clozapine**	0	Some concerns	Low risk	No concerns	Major concerns	No concerns	No concerns	Very low
**blonanserin:lurasidone**	0	No concerns	Low risk	No concerns	Major concerns	No concerns	No concerns	Very low
**blonanserin:molindone**	0	Some concerns	Low risk	No concerns	Major concerns	No concerns	No concerns	Very low
**blonanserin:olanzapine**	0	No concerns	Low risk	No concerns	Major concerns	No concerns	No concerns	Very low
**blonanserin:quetiapine**	0	No concerns	Low risk	No concerns	Major concerns	No concerns	No concerns	Very low
**blonanserin:risperidone/paliperidone**	0	No concerns	Low risk	No concerns	Major concerns	No concerns	No concerns	Very low
**blonanserin:ziprasidone**	0	No concerns	Low risk	No concerns	Major concerns	No concerns	No concerns	Very low
**clozapine:lurasidone**	0	Some concerns	Low risk	No concerns	Major concerns	No concerns	No concerns	Very low
**clozapine:molindone**	0	Some concerns	Low risk	No concerns	Major concerns	No concerns	No concerns	Very low
**clozapine:placebo**	0	Some concerns	Low risk	No concerns	Major concerns	No concerns	No concerns	Very low
**clozapine:quetiapine**	0	Some concerns	Low risk	No concerns	Major concerns	No concerns	No concerns	Very low

**clozapine:risperidone/paliperidone**	0	Some concerns	Low risk	No concerns	Major concerns	No concerns	No concerns	Very low
**clozapine:ziprasidone**	0	Some concerns	Low risk	No concerns	Major concerns	No concerns	No concerns	Very low
**lurasidone:molindone**	0	Some concerns	Low risk	No concerns	Major concerns	No concerns	No concerns	Very low
**lurasidone:olanzapine**	0	No concerns	Low risk	No concerns	Major concerns	No concerns	No concerns	Very low
**lurasidone:quetiapine**	0	No concerns	Low risk	No concerns	Major concerns	No concerns	No concerns	Very low
**lurasidone:risperidone/paliperidone**	0	No concerns	Low risk	No concerns	Major concerns	No concerns	No concerns	Very low
**lurasidone:ziprasidone**	0	No concerns	Low risk	No concerns	Major concerns	No concerns	No concerns	Very low
**molindone:placebo**	0	Some concerns	Low risk	No concerns	Major concerns	No concerns	No concerns	Very low
**molindone:quetiapine**	0	Some concerns	Low risk	No concerns	Major concerns	No concerns	No concerns	Very low
**molindone:ziprasidone**	0	Some concerns	Low risk	No concerns	Major concerns	No concerns	No concerns	Very low
**olanzapine:quetiapine**	0	No concerns	Low risk	No concerns	Major concerns	No concerns	No concerns	Very low
**olanzapine:ziprasidone**	0	Some concerns	Low risk	No concerns	Major concerns	No concerns	No concerns	Very low
**quetiapine:risperidone/paliperidone**	0	Some concerns	Low risk	No concerns	Major concerns	No concerns	No concerns	Very low
**quetiapine:ziprasidone**	0	No concerns	Low risk	No concerns	Major concerns	No concerns	No concerns	Very low
**risperidone/paliperidone:ziprasidone**	0	Some concerns	Low risk	No concerns	Major concerns	No concerns	No concerns	Very low

eTable 42. CINeMA confidence rating for total cholesterol

Comparison	Number of studies	Within-study bias	Reporting bias	Indirectness	Imprecision	Heterogeneity	Incoherence	Confidence rating
**aripiprazole:olanzapine**	1	Major concerns	Low risk	No concerns	No concerns	Some concerns	Some concerns	Very low
**aripiprazole:placebo**	3	Some concerns	Low risk	No concerns	No concerns	Major concerns	No concerns	Very low
**aripiprazole:quetiapine**	1	No concerns	Low risk	No concerns	No concerns	Some concerns	No concerns	Moderate
**aripiprazole:risperidone/paliperidone**	2	Some concerns	Low risk	No concerns	No concerns	Major concerns	No concerns	Very low
**asenapine:placebo**	2	No concerns	Low risk	No concerns	No concerns	Some concerns	Major concerns	Very low
**blonanserin:placebo**	1	No concerns	Low risk	No concerns	Major concerns	No concerns	Major concerns	Very low
**clozapine:olanzapine**	1	Some concerns	Low risk	No concerns	Major concerns	No concerns	Major concerns	Very low
**lurasidone:placebo**	2	No concerns	Low risk	No concerns	Some concerns	Some concerns	Major concerns	Very low
**molindone:olanzapine**	1	Some concerns	Low risk	No concerns	Some concerns	No concerns	Major concerns	Very low
**molindone:risperidone/paliperidone**	1	Some concerns	Low risk	No concerns	Major concerns	No concerns	Major concerns	Very low
**olanzapine:placebo**	1	Some concerns	Low risk	No concerns	No concerns	Some concerns	No concerns	Moderate
**olanzapine:risperidone/paliperidone**	2	Some concerns	Low risk	No concerns	No concerns	Some concerns	Some concerns	Low
**placebo:quetiapine**	3	No concerns	Low risk	No concerns	No concerns	Some concerns	Some concerns	Moderate
**placebo:risperidone/paliperidone**	4	Some concerns	Low risk	No concerns	No concerns	Major concerns	No concerns	Very low
**placebo:ziprasidone**	3	Some concerns	Low risk	No concerns	No concerns	Major concerns	Major concerns	Very low

**aripiprazole:asenapine**	0	No concerns	Low risk	No concerns	Some concerns	Some concerns	Major concerns	Very low
**aripiprazole:blonanserin**	0	No concerns	Low risk	No concerns	Major concerns	No concerns	Major concerns	Very low
**aripiprazole:clozapine**	0	Some concerns	Low risk	No concerns	Major concerns	No concerns	Major concerns	Very low
**aripiprazole:lurasidone**	0	No concerns	Low risk	No concerns	Major concerns	No concerns	Major concerns	Very low
**aripiprazole:molindone**	0	Some concerns	Low risk	No concerns	Major concerns	No concerns	Major concerns	Very low
**aripiprazole:ziprasidone**	0	Some concerns	Low risk	No concerns	Some concerns	Some concerns	Major concerns	Very low
**asenapine:blonanserin**	0	No concerns	Low risk	No concerns	Some concerns	Some concerns	Major concerns	Very low
**asenapine:clozapine**	0	Some concerns	Low risk	No concerns	Major concerns	No concerns	Major concerns	Very low
**asenapine:lurasidone**	0	No concerns	Low risk	No concerns	Some concerns	Some concerns	Major concerns	Very low
**asenapine:molindone**	0	Some concerns	Low risk	No concerns	Major concerns	No concerns	Major concerns	Very low
**asenapine:olanzapine**	0	No concerns	Low risk	No concerns	Some concerns	Some concerns	Major concerns	Very low
**asenapine:quetiapine**	0	No concerns	Low risk	No concerns	Some concerns	Some concerns	Major concerns	Very low
**asenapine:risperidone/paliperidone**	0	No concerns	Low risk	No concerns	Some concerns	Some concerns	Major concerns	Very low
**asenapine:ziprasidone**	0	No concerns	Low risk	No concerns	Some concerns	No concerns	Major concerns	Very low
**blonanserin:clozapine**	0	Some concerns	Low risk	No concerns	Major concerns	No concerns	Major concerns	Very low
**blonanserin:lurasidone**	0	No concerns	Low risk	No concerns	Major concerns	No concerns	Major concerns	Very low
**blonanserin:molindone**	0	Some concerns	Low risk	No concerns	Major concerns	No concerns	Major concerns	Very low
**blonanserin:olanzapine**	0	No concerns	Low risk	No concerns	Some concerns	No concerns	Major concerns	Very low

**blonanserin:quetiapine**	0	No concerns	Low risk	No concerns	Some concerns	No concerns	Major concerns	Very low
**blonanserin:risperidone/paliperidone**	0	No concerns	Low risk	No concerns	Major concerns	No concerns	Major concerns	Very low
**blonanserin:ziprasidone**	0	No concerns	Low risk	No concerns	Major concerns	No concerns	Major concerns	Very low
**clozapine:lurasidone**	0	Some concerns	Low risk	No concerns	Major concerns	No concerns	Major concerns	Very low
**clozapine:molindone**	0	Some concerns	Low risk	No concerns	Major concerns	No concerns	Major concerns	Very low
**clozapine:placebo**	0	Some concerns	Low risk	No concerns	Major concerns	No concerns	Major concerns	Very low
**clozapine:quetiapine**	0	Some concerns	Low risk	No concerns	Major concerns	No concerns	Major concerns	Very low
**clozapine:risperidone/paliperidone**	0	Some concerns	Low risk	No concerns	Major concerns	No concerns	Major concerns	Very low
**clozapine:ziprasidone**	0	Some concerns	Low risk	No concerns	Major concerns	No concerns	Major concerns	Very low
**lurasidone:molindone**	0	Some concerns	Low risk	No concerns	Major concerns	No concerns	Major concerns	Very low
**lurasidone:olanzapine**	0	No concerns	Low risk	No concerns	Some concerns	No concerns	Major concerns	Very low
**lurasidone:quetiapine**	0	No concerns	Low risk	No concerns	No concerns	Some concerns	Major concerns	Very low
**lurasidone:risperidone/paliperidone**	0	No concerns	Low risk	No concerns	Major concerns	No concerns	Major concerns	Very low
**lurasidone:ziprasidone**	0	No concerns	Low risk	No concerns	Major concerns	No concerns	Major concerns	Very low
**molindone:placebo**	0	Some concerns	Low risk	No concerns	Major concerns	No concerns	Major concerns	Very low
**molindone:quetiapine**	0	Some concerns	Low risk	No concerns	Some concerns	Some concerns	Major concerns	Very low
**molindone:ziprasidone**	0	Some concerns	Low risk	No concerns	Major concerns	No concerns	Major concerns	Very low
**olanzapine:quetiapine**	0	Some concerns	Low risk	No concerns	Major concerns	No concerns	Major concerns	Very low

**olanzapine:ziprasidone**	0	Some concerns	Low risk	No concerns	No concerns	Some concerns	Major concerns	Very low
**quetiapine:risperidone/paliperidone**	0	Some concerns	Low risk	No concerns	No concerns	Some concerns	Major concerns	Very low
**quetiapine:ziprasidone**	0	Some concerns	Low risk	No concerns	No concerns	Some concerns	Major concerns	Very low
**risperidone/paliperidone:ziprasidone**	0	Some concerns	Low risk	No concerns	Some concerns	Some concerns	Major concerns	Very low

eTable 43. CINeMA confidence rating for HDL cholesterol

Comparison	Number of studies	Within-study bias	Reporting bias	Indirectness	Imprecision	Heterogeneity	Incoherence	Confidence rating
**aripiprazole:olanzapine**	1	Major concerns	Low risk	No concerns	Major concerns	No concerns	No concerns	Very low
**aripiprazole:placebo**	2	No concerns	Low risk	No concerns	Major concerns	No concerns	No concerns	Very low
**aripiprazole:quetiapine**	1	No concerns	Low risk	No concerns	Major concerns	No concerns	No concerns	Very low
**aripiprazole:risperidone/paliperidone**	1	Major concerns	Low risk	No concerns	Major concerns	No concerns	No concerns	Very low
**molindone:olanzapine**	1	Some concerns	Low risk	No concerns	Major concerns	No concerns	No concerns	Very low
**molindone:risperidone/paliperidone**	1	Some concerns	Low risk	No concerns	Major concerns	No concerns	No concerns	Very low
**olanzapine:placebo**	1	Some concerns	Low risk	No concerns	Major concerns	No concerns	Major concerns	Very low
**olanzapine:risperidone/paliperidone**	2	Some concerns	Low risk	No concerns	Major concerns	No concerns	Major concerns	Very low
**placebo:quetiapine**	4	No concerns	Low risk	No concerns	Major concerns	No concerns	No concerns	Very low
**placebo:risperidone/paliperidone**	2	Some concerns	Low risk	No concerns	No concerns	Major concerns	Major concerns	Very low
**placebo:ziprasidone**	3	Some concerns	Low risk	No concerns	Major concerns	No concerns	Major concerns	Very low
**aripiprazole:molindone**	0	Some concerns	Low risk	No concerns	Major concerns	No concerns	Major concerns	Very low

**aripiprazole:ziprasidone**	0	Some concerns	Low risk	No concerns	Major concerns	No concerns	Major concerns	Very low
**molindone:placebo**	0	Some concerns	Low risk	No concerns	Major concerns	No concerns	Major concerns	Very low
**molindone:quetiapine**	0	Some concerns	Low risk	No concerns	Major concerns	No concerns	Major concerns	Very low
**molindone:ziprasidone**	0	Some concerns	Low risk	No concerns	Major concerns	No concerns	Major concerns	Very low
**olanzapine:quetiapine**	0	Some concerns	Low risk	No concerns	Major concerns	No concerns	Major concerns	Very low
**olanzapine:ziprasidone**	0	Some concerns	Low risk	No concerns	Major concerns	No concerns	Major concerns	Very low
**quetiapine:risperidone/paliperidone**	0	No concerns	Low risk	No concerns	No concerns	Major concerns	Major concerns	Very low
**quetiapine:ziprasidone**	0	Some concerns	Low risk	No concerns	Major concerns	No concerns	Major concerns	Very low
**risperidone/paliperidone:ziprasidone**	0	Some concerns	Low risk	No concerns	Some concerns	Some concerns	Major concerns	Very low

eTable 44. CINeMA confidence rating for LDL cholesterol

Comparison	Number of studies	Within-study bias	Reporting bias	Indirectness	Imprecision	Heterogeneity	Incoherence	Confidence rating
**aripiprazole:olanzapine**	1	Major concerns	Low risk	No concerns	No concerns	Major concerns	Major concerns	Very low
**aripiprazole:placebo**	1	No concerns	Low risk	No concerns	Major concerns	No concerns	No concerns	Very low
**aripiprazole:quetiapine**	1	No concerns	Low risk	No concerns	Some concerns	Some concerns	No concerns	Moderate
**aripiprazole:risperidone/paliperidone**	2	Major concerns	Low risk	No concerns	Some concerns	Some concerns	No concerns	Very low

**lurasidone:placebo**	1	Some concerns	Low risk	No concerns	Major concerns	No concerns	Major concerns	Very low
**molindone:olanzapine**	1	Some concerns	Low risk	No concerns	Some concerns	Some concerns	Major concerns	Very low
**molindone:risperidone/paliperidone**	1	Some concerns	Low risk	No concerns	Major concerns	No concerns	Major concerns	Very low
**olanzapine:placebo**	1	Some concerns	Low risk	No concerns	Some concerns	Some concerns	No concerns	Moderate
**olanzapine:risperidone/paliperidone**	2	Some concerns	Low risk	No concerns	No concerns	Some concerns	No concerns	Moderate
**placebo:quetiapine**	2	No concerns	Low risk	No concerns	Some concerns	Some concerns	No concerns	Moderate
**placebo:risperidone/paliperidone**	2	No concerns	Low risk	No concerns	Some concerns	Some concerns	No concerns	Moderate
**placebo:ziprasidone**	3	Some concerns	Low risk	No concerns	Some concerns	Some concerns	Major concerns	Very low
**aripiprazole:lurasidone**	0	Some concerns	Low risk	No concerns	Major concerns	No concerns	Major concerns	Very low
**aripiprazole:molindone**	0	Some concerns	Low risk	No concerns	Major concerns	No concerns	Major concerns	Very low
**aripiprazole:ziprasidone**	0	Some concerns	Low risk	No concerns	Major concerns	No concerns	Major concerns	Very low
**lurasidone:molindone**	0	Some concerns	Low risk	No concerns	Major concerns	No concerns	Major concerns	Very low
**lurasidone:olanzapine**	0	Some concerns	Low risk	No concerns	Some concerns	Some concerns	Major concerns	Very low
**lurasidone:quetiapine**	0	Some concerns	Low risk	No concerns	Major concerns	No concerns	Major concerns	Very low
**lurasidone:risperidone/paliperidone**	0	Some concerns	Low risk	No concerns	Major concerns	No concerns	Major concerns	Very low
**lurasidone:ziprasidone**	0	Some concerns	Low risk	No concerns	Major concerns	No concerns	Major concerns	Very low
**molindone:placebo**	0	Some concerns	Low risk	No concerns	Major concerns	No concerns	Major concerns	Very low
**molindone:quetiapine**	0	Some concerns	Low risk	No concerns	Major concerns	No concerns	Major concerns	Very low

**molindone:ziprasidone**	0	Some concerns	Low risk	No concerns	Major concerns	No concerns	Major concerns	Very low
**olanzapine:quetiapine**	0	Some concerns	Low risk	No concerns	Some concerns	Some concerns	Major concerns	Very low
**olanzapine:ziprasidone**	0	Some concerns	Low risk	No concerns	Some concerns	Some concerns	Major concerns	Very low
**quetiapine:risperidone/paliperidone**	0	No concerns	Low risk	No concerns	Some concerns	Some concerns	Major concerns	Very low
**quetiapine:ziprasidone**	0	Some concerns	Low risk	No concerns	Major concerns	No concerns	Major concerns	Very low
**risperidone/paliperidone:ziprasidone**	0	Some concerns	Low risk	No concerns	Major concerns	No concerns	Major concerns	Very low

eTable 45. CINeMA confidence rating for QTc interval

Comparison	Number of studies	Within-study bias	Reporting bias	Indirectness	Imprecision	Heterogeneity	Incoherence	Confidence rating
**aripiprazole:placebo**	1	Some concerns	Low risk	No concerns	Some concerns	Some concerns	No concerns	Low
**aripiprazole:quetiapine**	1	No concerns	Low risk	No concerns	Some concerns	Some concerns	No concerns	Moderate
**molindone:olanzapine**	1	Some concerns	Low risk	No concerns	Some concerns	Some concerns	No concerns	Low
**molindone:risperidone/paliperidone**	1	Some concerns	Low risk	No concerns	Major concerns	No concerns	No concerns	Very low
**olanzapine:placebo**	2	No concerns	Low risk	No concerns	Major concerns	No concerns	No concerns	Very low
**olanzapine:risperidone/paliperidone**	1	Some concerns	Low risk	No concerns	Some concerns	Some concerns	No concerns	Low
**pimozide:risperidone/paliperidone**	1	Some concerns	Low risk	No concerns	Major concerns	No concerns	No concerns	Very low
**placebo:quetiapine**	1	Some concerns	Low risk	No concerns	Some concerns	Some concerns	No concerns	Low

**placebo:risperidone/paliperidone**	2	No concerns	Low risk	No concerns	Some concerns	Some concerns	No concerns	Moderate
**placebo:ziprasidone**	1	Some concerns	Low risk	No concerns	Some concerns	Some concerns	No concerns	Low
**aripiprazole:molindone**	0	Some concerns	Low risk	No concerns	Major concerns	No concerns	No concerns	Very low
**aripiprazole:olanzapine**	0	Some concerns	Low risk	No concerns	Major concerns	No concerns	No concerns	Very low
**aripiprazole:pimozide**	0	Some concerns	Low risk	No concerns	Major concerns	No concerns	No concerns	Very low
**aripiprazole:risperidone/paliperidone**	0	Some concerns	Low risk	No concerns	Major concerns	No concerns	No concerns	Very low
**aripiprazole:ziprasidone**	0	Some concerns	Low risk	No concerns	Some concerns	Some concerns	No concerns	Low
**molindone:pimozide**	0	Some concerns	Low risk	No concerns	Major concerns	No concerns	No concerns	Very low
**molindone:placebo**	0	Some concerns	Low risk	No concerns	Some concerns	Some concerns	No concerns	Low
**molindone:quetiapine**	0	Some concerns	Low risk	No concerns	Some concerns	Some concerns	No concerns	Low
**molindone:ziprasidone**	0	Some concerns	Low risk	No concerns	No concerns	Major concerns	No concerns	Very low
**olanzapine:pimozide**	0	Some concerns	Low risk	No concerns	Major concerns	No concerns	No concerns	Very low
**olanzapine:quetiapine**	0	No concerns	Low risk	No concerns	Major concerns	No concerns	No concerns	Very low
**olanzapine:ziprasidone**	0	Some concerns	Low risk	No concerns	Some concerns	Some concerns	No concerns	Low
**pimozide:placebo**	0	Some concerns	Low risk	No concerns	Major concerns	No concerns	No concerns	Very low
**pimozide:quetiapine**	0	Some concerns	Low risk	No concerns	Some concerns	Some concerns	No concerns	Low
**pimozide:ziprasidone**	0	Some concerns	Low risk	No concerns	Some concerns	Some concerns	No concerns	Low
**quetiapine:risperidone/paliperidone**	0	Some concerns	Low risk	No concerns	Some concerns	Some concerns	No concerns	Low

**quetiapine:ziprasidone**	0	Some concerns	Low risk	No concerns	Major concerns	No concerns	No concerns	Very low
**risperidone/paliperidone:ziprasidone**	0	Some concerns	Low risk	No concerns	No concerns	Major concerns	No concerns	Very low

eTable 46. CINeMA confidence rating for heart rate

Comparison	Number of studies	Within-study bias	Reporting bias	Indirectness	Imprecision	Heterogeneity	Incoherence	Confidence rating
**aripiprazole:quetiapine**	1	No concerns	Low risk	No concerns	No concerns	Major concerns	No concerns	Very low
**aripiprazole:risperidone/paliperidone**	1	Some concerns	Low risk	No concerns	Major concerns	No concerns	No concerns	Very low
**haloperidol:risperidone/paliperidone**	1	Some concerns	Low risk	No concerns	Major concerns	No concerns	No concerns	Very low
**olanzapine:placebo**	1	No concerns	Low risk	No concerns	Major concerns	No concerns	No concerns	Very low
**placebo:quetiapine**	1	Some concerns	Low risk	No concerns	No concerns	Major concerns	No concerns	Very low
**placebo:risperidone/paliperidone**	2	Some concerns	Low risk	No concerns	Some concerns	Some concerns	No concerns	Low
**placebo:ziprasidone**	1	Some concerns	Low risk	No concerns	Major concerns	No concerns	No concerns	Very low
**aripiprazole:haloperidol**	0	Some concerns	Low risk	No concerns	Major concerns	No concerns	No concerns	Very low
**aripiprazole:olanzapine**	0	Some concerns	Low risk	No concerns	Major concerns	No concerns	No concerns	Very low
**aripiprazole:placebo**	0	Some concerns	Low risk	No concerns	Major concerns	No concerns	No concerns	Very low
**aripiprazole:ziprasidone**	0	Some concerns	Low risk	No concerns	Major concerns	No concerns	No concerns	Very low
**haloperidol:olanzapine**	0	Some concerns	Low risk	No concerns	Major concerns	No concerns	No concerns	Very low

**haloperidol:placebo**	0	Some concerns	Low risk	No concerns	Some concerns	Some concerns	No concerns	Low
**haloperidol:quetiapine**	0	Some concerns	Low risk	No concerns	Major concerns	No concerns	No concerns	Very low
**haloperidol:ziprasidone**	0	Some concerns	Low risk	No concerns	Major concerns	No concerns	No concerns	Very low
**olanzapine:quetiapine**	0	No concerns	Low risk	No concerns	Some concerns	Some concerns	No concerns	Moderate
**olanzapine:risperidone/paliperidone**	0	No concerns	Low risk	No concerns	Major concerns	No concerns	No concerns	Very low
**olanzapine:ziprasidone**	0	No concerns	Low risk	No concerns	Major concerns	No concerns	No concerns	Very low
**quetiapine:risperidone/paliperidone**	0	Some concerns	Low risk	No concerns	Some concerns	Some concerns	No concerns	Low
**quetiapine:ziprasidone**	0	Some concerns	Low risk	No concerns	No concerns	Major concerns	No concerns	Very low
**risperidone/paliperidone:ziprasidone**	0	Some concerns	Low risk	No concerns	Major concerns	No concerns	No concerns	Very low

eTable 47. CINeMA confidence rating for systolic blood pressure

Comparison	Number of studies	Within-study bias	Reporting bias	Indirectness	Imprecision	Heterogeneity	Incoherence	Confidence rating
**aripiprazole:placebo**	1	Some concerns	Low risk	No concerns	No concerns	Major concerns	No concerns	Very low
**aripiprazole:quetiapine**	1	No concerns	Low risk	No concerns	No concerns	Major concerns	No concerns	Very low
**aripiprazole:risperidone/paliperidone**	1	Some concerns	Low risk	No concerns	No concerns	Major concerns	No concerns	Very low
**clozapine:haloperidol**	1	No concerns	Low risk	No concerns	Major concerns	No concerns	No concerns	Very low
**haloperidol:risperidone/paliperidone**	1	Some concerns	Low risk	No concerns	Some concerns	Some concerns	No concerns	Low

**olanzapine:placebo**	1	No concerns	Low risk	No concerns	No concerns	Major concerns	No concerns	Very low
**placebo:quetiapine**	1	Some concerns	Low risk	No concerns	No concerns	Major concerns	No concerns	Very low
**placebo:risperidone/paliperidone**	1	Some concerns	Low risk	No concerns	No concerns	Major concerns	No concerns	Very low
**placebo:ziprasidone**	1	Some concerns	Low risk	No concerns	No concerns	Major concerns	No concerns	Very low
**aripiprazole:clozapine**	0	Some concerns	Low risk	No concerns	Some concerns	Some concerns	No concerns	Low
**aripiprazole:haloperidol**	0	Some concerns	Low risk	No concerns	Some concerns	Some concerns	No concerns	Low
**aripiprazole:olanzapine**	0	Some concerns	Low risk	No concerns	Some concerns	Some concerns	No concerns	Low
**aripiprazole:ziprasidone**	0	Some concerns	Low risk	No concerns	No concerns	Major concerns	No concerns	Very low
**clozapine:olanzapine**	0	Some concerns	Low risk	No concerns	Some concerns	Some concerns	No concerns	Low
**clozapine:placebo**	0	Some concerns	Low risk	No concerns	Major concerns	No concerns	No concerns	Very low
**clozapine:quetiapine**	0	Some concerns	Low risk	No concerns	No concerns	Major concerns	No concerns	Very low
**clozapine:risperidone/paliperidone**	0	No concerns	Low risk	No concerns	Some concerns	Some concerns	No concerns	Low
**clozapine:ziprasidone**	0	Some concerns	Low risk	No concerns	Major concerns	No concerns	No concerns	Very low
**haloperidol:olanzapine**	0	Some concerns	Low risk	No concerns	Some concerns	Some concerns	No concerns	Low
**haloperidol:placebo**	0	Some concerns	Low risk	No concerns	Some concerns	Some concerns	No concerns	Low
**haloperidol:quetiapine**	0	Some concerns	Low risk	No concerns	No concerns	Major concerns	No concerns	Very low

**haloperidol:ziprasidone**	0	Some concerns	Low risk	No concerns	Some concerns	Some concerns	No concerns	Low
**olanzapine:quetiapine**	0	No concerns	Low risk	No concerns	Some concerns	Some concerns	No concerns	Moderate
**olanzapine:risperidone/paliperidone**	0	Some concerns	Low risk	No concerns	Some concerns	Some concerns	No concerns	Low
**olanzapine:ziprasidone**	0	No concerns	Low risk	No concerns	No concerns	Major concerns	No concerns	Very low
**quetiapine:risperidone/paliperidone**	0	Some concerns	Low risk	No concerns	No concerns	Major concerns	No concerns	Very low
**quetiapine:ziprasidone**	0	Some concerns	Low risk	No concerns	No concerns	Major concerns	No concerns	Very low
**risperidone/paliperidone:ziprasidone**	0	Some concerns	Low risk	No concerns	No concerns	Major concerns	No concerns	Very low

eAppendix 7. Sensitivity analysis: antipsychotic-induced physiological changes in children and young people with schizophrenia

There were sufficient studies to perform sensitivity analyses for change in weight, BMI, total cholesterol, HDL cholesterol, LDL cholesterol, triglycerides, glucose, and prolactin levels. Results are described below, a forest plot is also presented showing result for changes in weight, BMI, total cholesterol, triglycerides, glucose, and prolactin. Compared to the main analyses, results of sensitivity analyses were not materially different both in terms of magnitude of antipsychotic-induced physiological changes and the relative degree of physiological changes associated with different antipsychotics.

Changes in body weight and BMI

For change in weight, 12 studies compared 10 different antipsychotics with placebo (1474 and 448 patients, respectively). There was clear evidence of weight gain (MD relative to placebo/kg, 95%CI) with olanzapine (3.91, 2.17 to 5.65) and quetiapine (2.42, 0.64 to 4.21). There was some evidence of weight gain with clozapine (4.11, -0.55 to 8.77), haloperidol (4.15, -2.41 to 10.71), and risperidone/paliperidone (1.11, -0.13 to 2.36), although uncertainty was larger. We found no evidence of weight gain with aripiprazole, blonanserin, lurasidone, ziprasidone or molindone. For BMI, 7 studies compared 6 different antipsychotics with placebo (764 and 113 patients, respectively). There was clear evidence of increase in BMI (MD relative to placebo (kg/m^2), 95%CI) with quetiapine (2.11, 0.58 to 3.64), clozapine (1.61, 0.10 to 3.11), olanzapine (1.59, 1.13 to 2.04), risperidone/paliperidone (0.77, 0.46 to 1.08), and aripiprazole (0.29, 0.07 to 0.51). There was no evidence of increase in BMI observed with molindone.

Changes in lipids

For total-cholesterol, 11 studies compared 10 different antipsychotics with placebo (1522 and 499 patients, respectively). Compared with placebo, there was evidence of an increase in total cholesterol (MD relative to placebo (mmol/L), 95%CI) with quetiapine (0.46, 0.00 to 0.92). There was some evidence of an effect of olanzapine (0.43, -0.08 to 0.95), and very weak evidence (very large uncertainty) of an effect of clozapine (0.40, -0.52 to 1.32) and asenapine (0.24, -0.32 to 0.79). For lurasidone, molindone, ziprasidone, blonanserin and risperidone/paliperidone, there was no evidence. For LDL-cholesterol, 7 studies compared 7 different antipsychotics with placebo (980 and 252 patients, respectively). For HDL-cholesterol, 6 studies compared 6 different antipsychotics with placebo (736 and 238 patients, respectively. There was little to no evidence of any

antipsychotics altering these parameters. For triglycerides, 11 studies compared 10 different antipsychotics with placebo (1522 and 499 patients, respectively). There was clear evidence of an increase in triglyceride levels (MD relative to placebo (mmol/L),95%CI) with clozapine (0.62, 0.04 to 1.20), olanzapine (0.39, 0.13 to 0.65) and risperidone/paliperidone (0.21, 0.02 to 0.40). Furthermore, there was evidence of increased triglycerides with quetiapine (0.13, 0.00 to 0.26), and weaker evidence for blonanserin (0.14, -0.04 to 0.31), and aripiprazole (0.11, -0.01 to 0.24). There was no clear evidence of change in triglycerides with ziprasidone, asenapine, lurasidone and molindone.

Changes in glucose

For glucose, 11 studies compared 10 different antipsychotics with placebo (1522 and 499 patients, respectively). There was strong evidence of an increase in glucose levels (MD relative to placebo (mmol/L), 95%CI) with clozapine (0.81, 0.28 to 1.34), risperidone/paliperidone (0.28, 0.07 to 0.49), olanzapine (0.25, 0.00 to 0.49), and asenapine (0.20, 0.05 to 0.35). There was some evidence of increased glucose with molindone (0.26, -0.04 to 0.57), aripiprazole (0.13, -0.01 to 0.27), and quetiapine (0.12, -0.02 to 0.26). We observed a decrease in fasting-glucose with ziprasidone (-0.33, -0.64 to -0.02), blonanserin (-0.09, -0.24 to 0.07), and lurasidone (-0.09, -0.24 to 0.07), although with considerable uncertainty for the 2 latter antipsychotics.

Changes in prolactin

For prolactin, 11 studies compared 8 different antipsychotics with placebo (1564 and 533 patients, respectively). There was very strong evidence of an increase in prolactin levels (MD relative to placebo (ng/mL), 95%CI) with risperidone/paliperidone (28.10, 16.23 to 39.96). We did not find strong evidence of change in prolactin with aripiprazole, asenapine, molindone, quetiapine, lurasidone, blonanserin or olanzapine.

eFigure 8. Estimates of mean differences of antipsychotic drugs compared with placebo obtained from network meta-analyses. Colours indicate the confidence in the evidence for a given comparison: blue is low, and red is very low. Confidence of outcomes was graded using the Confidence in Network Meta-Analysis application. Grey lines immediately below each coloured line indicate the prediction interval (PI) corresponding to that antipsychotic-placebo comparison

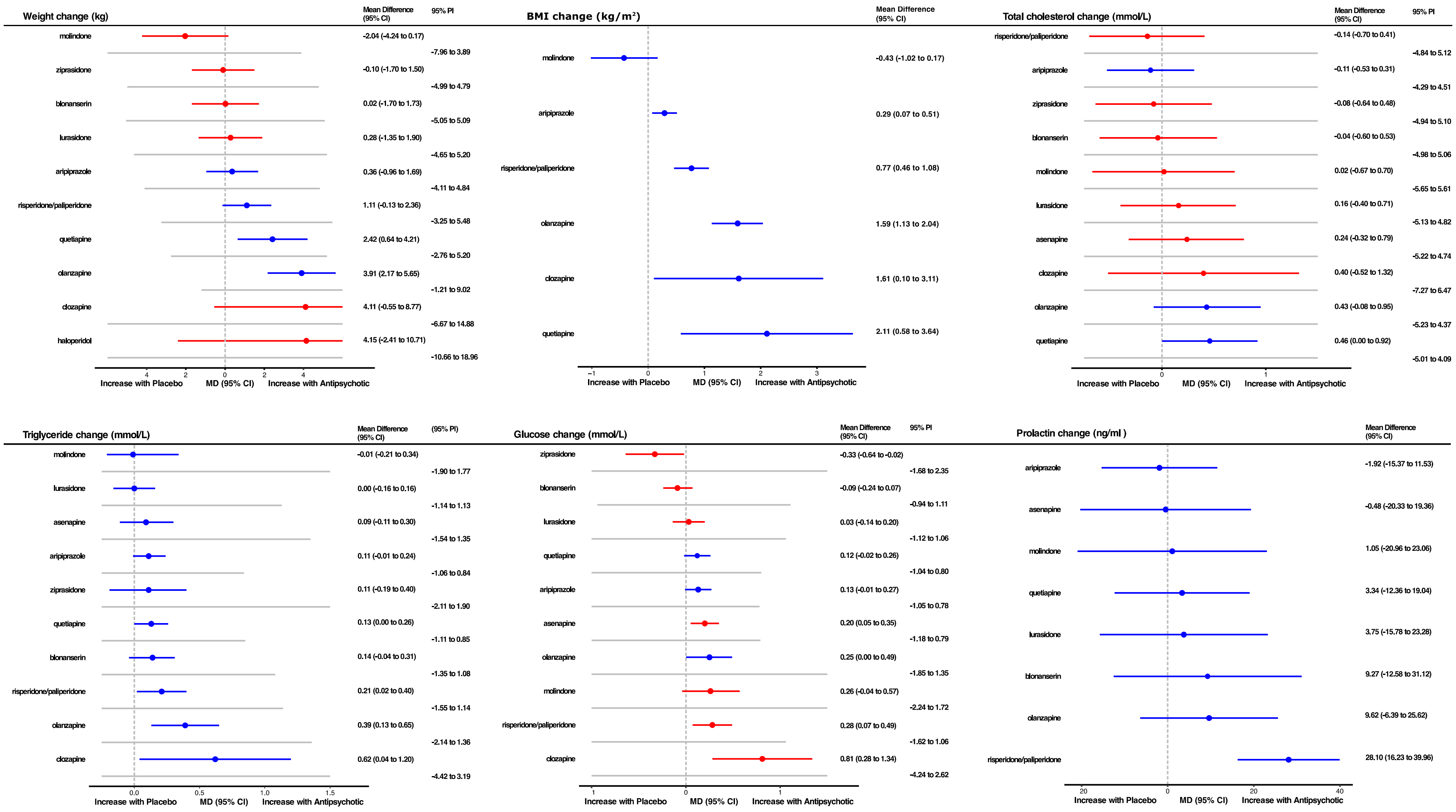

eAppendix 8

QQ plots for pairwise meta-regression models

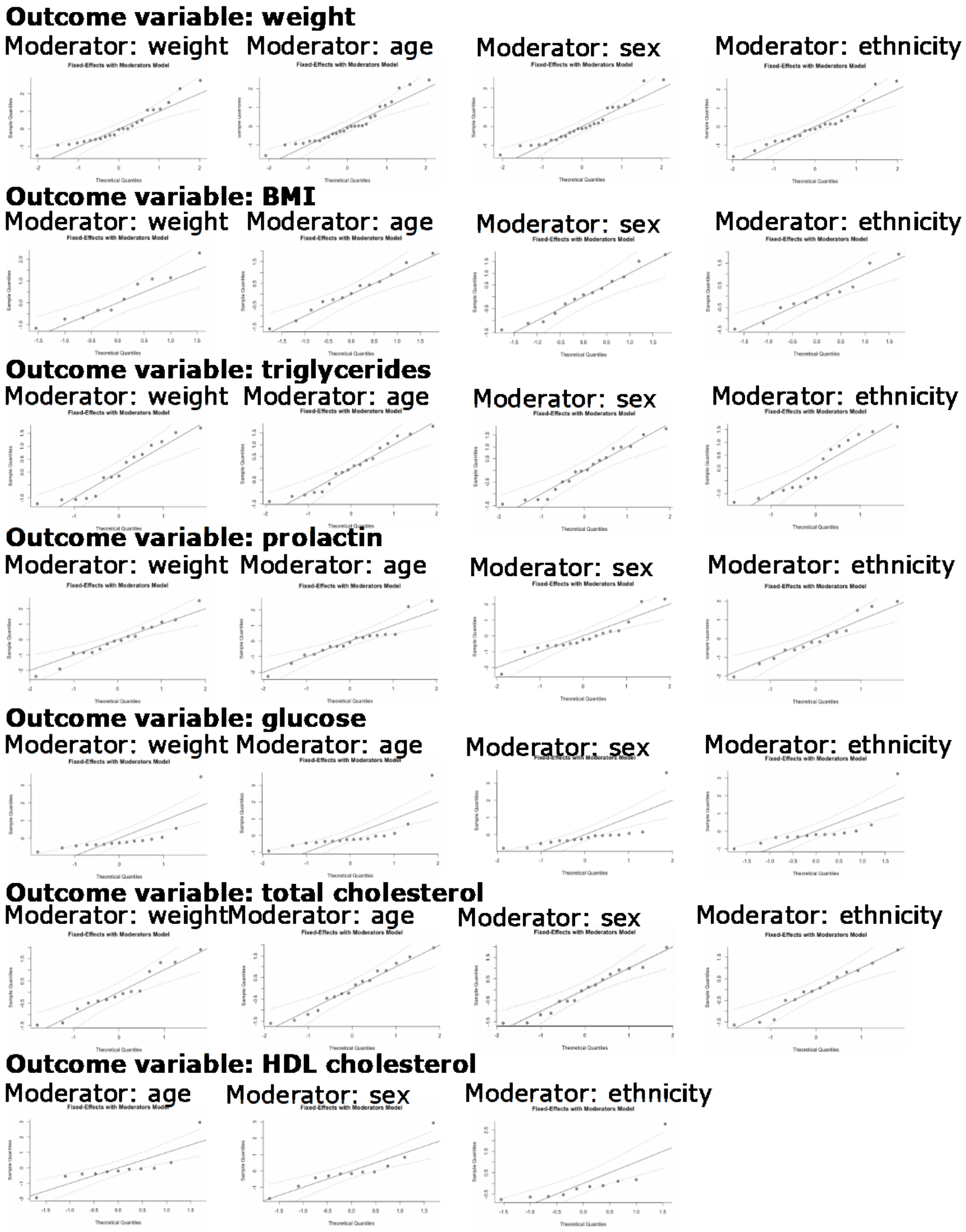

eAppendix 9. Residual versus fitted plots for pairwise meta-regression models

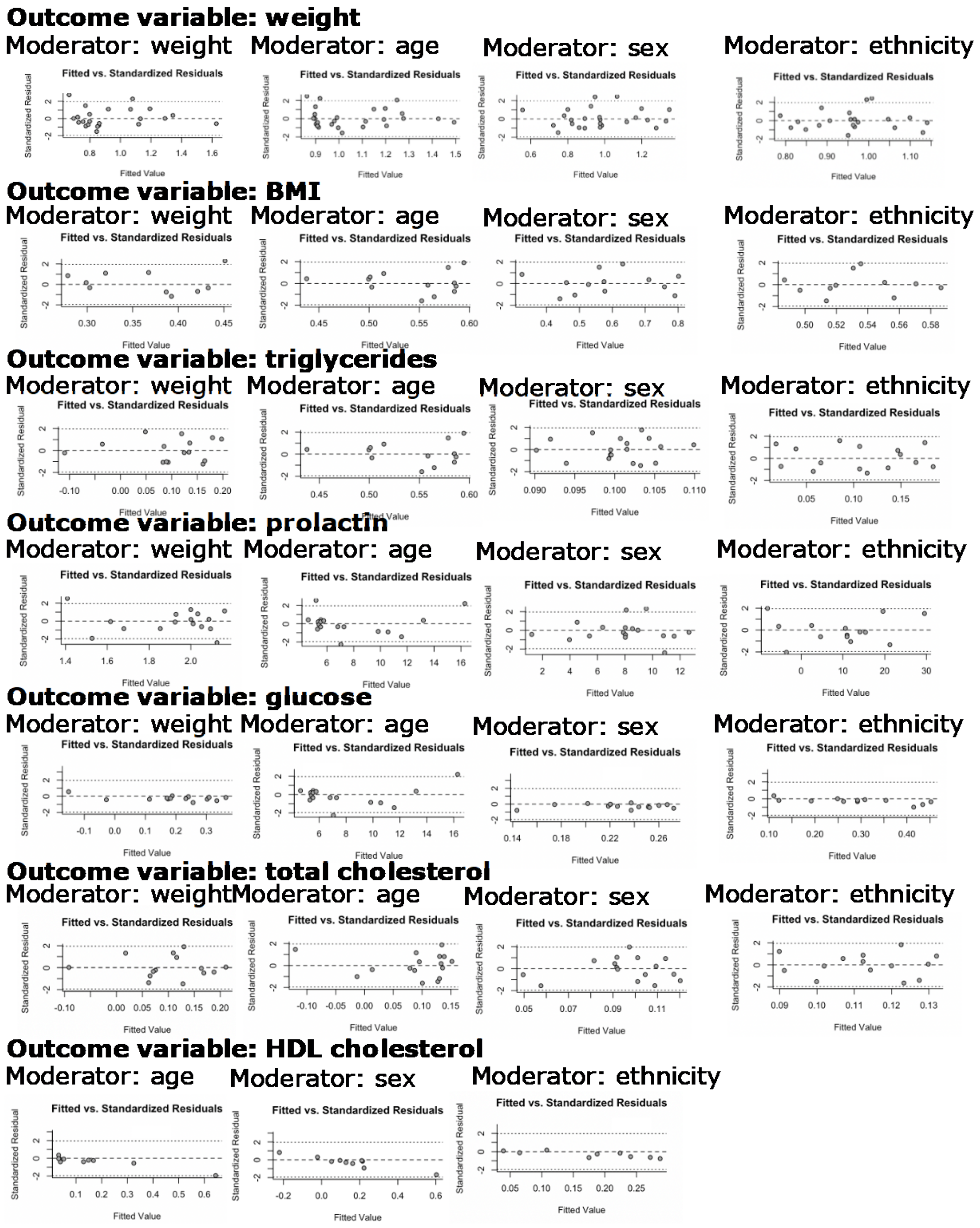

eAppendix 10. Results: Meta-regression on the effect of sex, ethnicity, and baseline body weight on antipsychotic-induced physiological alterations

There were sufficient studies for us to perform meta-regression analyses assessing 4 possible effect modifiers (mean age, mean baseline body weight, proportion of males in study population, proportion of non-Caucasians in study population) of antipsychotic-induced physiological changes, as follows: changes in weight, BMI, triglycerides, prolactin, glucose, total cholesterol, and HDL cholesterol.

Moderator	Number of studies	Estimate, 95% CI	p-value	p_{FDR}
Outcome variable: weight				
**Baseline weight**	23	-0.02 (0.06 to 0.02)	0.31	0.62
**Baseline age**	27	-0.06 (-0.21 to 0.10)	0.48	0.56
**% male**	25	0.01 (-0.02 to 0.04)	0.40	0.90
**% non-Caucasian**	21	-0.01 (-0.06 to 0.04)	0.70	0.83
Outcome variable: BMI				
**Baseline weight**	23	0.01 (-0.03 to 0.04)	0.74	0.89
**Baseline age**	13	0.02 (-0.09 to 0.12)	0.77	0.77
**% male**	13	0.01 (-0.01 to 0.02)	0.31	0.90
**% non-Caucasian**	11	-0.00 (-0.04 to 0.03)	0.83	0.83
Outcome variable: triglycerides				
**Baseline weight**	15	0.01 (-0.00 to 0.03)	0.12	0.45
**Baseline age**	18	0.01 (-0.02 to 0.05)	0.45	0.56
**% male**	18	-0.00 (-0.01 to 0.01)	0.90	0.90
**% non-Caucasian**	15	0.01 (-0.00 to 0.01)	0.12	0.42
Outcome variable: prolactin				
**Baseline weight**	16	0.02 (-0.43 to 0.48)	0.92	0.92
**Baseline age**	17	-1.16 (-4.28 to 1.95)	0.47	0.56
**% male**	17	-0.22 (-0.92 to 0.47)	0.53	0.90
**% non-Caucasian**	14	-1.11 (-2.10 to -0.11)	0.029	0.20
Outcome variable: glucose				
**Baseline weight**	15	0.02 (-0.04 to 0.08)	0.52	0.78
**Baseline age**	16	0.11 (-0.12 to 0.33)	0.37	0.56
**% male**	16	-0.00 (-0.03 to 0.03)	0.87	0.90
**% non-Caucasian**	13	0.01 (-0.03 to 0.06)	0.64	0.83
Outcome variable: total cholesterol				
**Baseline weight**	14	0.01 (-0.01 to 0.03)	0.15	0.45
**Baseline age**	16	0.03 (-0.01 to 0.06)	0.14	0.49
**% male**	16	-0.00 (-0.01 to 0.01)	0.69	0.90
**% non-Caucasian**	14	0.00 (-0.01 to 0.01)	0.75	0.83
Outcome variable: HDL cholesterol				
**Baseline weight**	Insufficient studies (k=9)			
**Baseline age**	11	-0.07 (-0.15 to 0.02)	0.12	0.49
**% male**	11	0.01 (0.00 to 0.03)	0.05	0.35
**% non-Caucasian**	10	0.01 (-0.02 to 0.04)	0.57	0.83

Supplementary References

1. Viechtbauer W. Conducting Meta-Analyses in R with the metafor Package. *Journal of Statistical Software* 2010; **36**(3)**:** 1-48.

2. Riley RD, Thompson JR, Abrams KR. An alternative model for bivariate random-effects meta-analysis when the within-study correlations are unknown. *Biostatistics* 2008; **9**(1)**:** 172-186.

3. Aman MG, De Smedt G, Derivan A, Lyons B, Findling RL. Double-blind, placebo-controlled study of risperidone for the treatment of disruptive behaviors in children with subaverage intelligence. *Am J Psychiatry* 2002; **159**(8)**:** 1337-1346.

4. Buitelaar JK, van der Gaag RJ, Cohen-Kettenis P, Melman CT. A randomized controlled trial of risperidone in the treatment of aggression in hospitalized adolescents with subaverage cognitive abilities. *J Clin Psychiatry* 2001; **62**(4)**:** 239-248.

5. Connor DF, McLaughlin TJ, Jeffers-Terry M. Randomized controlled pilot study of quetiapine in the treatment of adolescent conduct disorder. *J Child Adolesc Psychopharmacol* 2008; **18**(2)**:** 140-156.

6. DelBello MP, Chang K, Welge JA, Adler CM, Rana M, Howe M *et al.* A double-blind, placebo-controlled pilot study of quetiapine for depressed adolescents with bipolar disorder. *Bipolar Disord* 2009; **11**(5)**:** 483-493.

7. DelBello MP, Goldman R, Phillips D, Deng L, Cucchiaro J, Loebel A. Efficacy and Safety of Lurasidone in Children and Adolescents With Bipolar I Depression: A Double-Blind, Placebo-Controlled Study. *J Am Acad Child Adolesc Psychiatry* 2017; **56**(12)**:** 1015-1025.

8. Findling RL, McNamara NK, Branicky LA, Schluchter MD, Lemon E, Blumer JL. A double-blind pilot study of risperidone in the treatment of conduct disorder. *Journal of the American Academy of Child and Adolescent Psychiatry* 2000; **39(4):** 509-516.

9. Findling RL, Robb A, Nyilas M, Forbes RA, Jin N, Ivanova S *et al.* A multiple-center, randomized, double-blind, placebo-controlled study of oral aripiprazole for treatment of adolescents with schizophrenia. *American Journal of Psychiatry* 2008; **165**(11)**:** 1432-1441.

10. Findling RL, Nyilas M, Forbes RA, McQuade RD, Jin N, Iwamoto T *et al.* Acute treatment of pediatric bipolar I disorder, manic or mixed episode, with aripiprazole: A randomized, double-blind, placebo-controlled study. *Journal of Clinical Psychiatry* 2009; **70(10):** 1441-1451.

11. Findling RL, McKenna K, Earley WR, Stankowski J, Pathak S. Efficacy and safety of quetiapine in adolescents with schizophrenia investigated in a 6-week, double-blind, placebo-controlled trial. *Journal of Child and Adolescent Psychopharmacology* 2012; **22**(5)**:** 327-342.

12. Findling RL, Cavus I, Pappadopulos E, Vanderburg DG, Schwartz JH, Gundapaneni BK *et al.* Efficacy, long-term safety, and tolerability of Ziprasidone in children and adolescents with bipolar disorder. *Journal of Child and Adolescent Psychopharmacology* 2013; **23(8):** 545-557.

13. Findling RL, Cavus I, Pappadopulos E, Vanderburg DG, Schwartz JH, Gundapaneni BK *et al.* Ziprasidone in adolescents with schizophrenia: Results from a placebo-controlled efficacy and long-term open-extension study. *Journal of Child and Adolescent Psychopharmacology* 2013; **23**(8)**:** 531-544.

14. Findling RL, Pathak S, Earley WR, Liu S, DelBello MP. Efficacy and safety of extended-release quetiapine fumarate in youth with bipolar depression: An 8 week, double-blind, placebo-controlled trial. *Journal of Child and Adolescent Psychopharmacology* 2014; **24(6):** 325-335.

15. Findling RL, Landbloom RP, Mackle M, Pallozzi W, Braat S, Hundt C *et al.* Safety and Efficacy from an 8 Week Double-Blind Trial and a 26 Week Open-Label Extension of Asenapine in Adolescents with Schizophrenia. *Journal of Child and Adolescent Psychopharmacology* 2015; **25**(5)**:** 384-396.

16. Findling RL, Landbloom RL, Szegedi A, Koppenhaver J, Braat S, Zhu Q *et al.* Asenapine for the Acute Treatment of Pediatric Manic or Mixed Episode of Bipolar i Disorder. *Journal of the American Academy of Child and Adolescent Psychiatry* 2015; **54(12):** 1032-1041.

17. Findling RL, Youngstrom EA, Rowles BM, Deyling E, Lingler J, Stansbrey RJ *et al.* A Double-Blind and Placebo-Controlled Trial of Aripiprazole in Symptomatic Youths at Genetic High Risk for Bipolar Disorder. *Journal of Child and Adolescent Psychopharmacology* 2017; **27(10):** 864-874.

18. Findling RL, Atkinson S, Bachinsky M, Raiter Y, Abreu P, Ianos C *et al.* Efficacy, Safety, and Tolerability of Flexibly Dosed Ziprasidone in Children and Adolescents with Mania in Bipolar I Disorder: A Randomized Placebo-Controlled Replication Study. *Journal of Child and Adolescent Psychopharmacology* 2022; **32(3):** 143-152.

19. Gilbert DL, Batterson JR, Sethuraman G, Sallee FR. Tic reduction with risperidone versus pimozide in a randomized, double-blind, crossover trial. *Journal of the American Academy of Child and Adolescent Psychiatry* 2004; **43(2):** 206-214.

20. Goldman R, Loebel A, Cucchiaro J, Deng L, Findling RL. Efficacy and Safety of Lurasidone in Adolescents with Schizophrenia: A 6-Week, Randomized Placebo-Controlled Study. *J Child Adolesc Psychopharmacol* 2017; **27**(6)**:** 516-525.

21. Haas M, Unis AS, Armenteros J, Copenhaver MD, Quiroz JA, Kushner SF. A 6-week, randomized, double-blind, placebo-controlled study of the efficacy and safety of risperidone in adolescents with schizophrenia. *Journal of Child and Adolescent Psychopharmacology* 2009; **19**(6)**:** 611-621.

22. Haas M, DelBello MP, Pandina G, Kushner S, Van Hove I, Augustyns I *et al.* Risperidone for the treatment of acute mania in children and adolescents with bipolar disorder: A randomized, double-blind, placebo-controlled study. *Bipolar Disorders* 2009; **11(7):** 687-700.

23. Hagman J, Gralla J, Sigel E, Ellert S, Dodge M, Gardner R *et al.* A double-blind, placebo-controlled study of risperidone for the treatment of adolescents and young adults with anorexia nervosa: A pilot study. *Journal of the American Academy of Child and Adolescent Psychiatry* 2011; **50(9):** 915-924.

24. Ichikawa H, Mikami K, Okada T, Yamashita Y, Ishizaki Y, Tomoda A *et al.* Aripiprazole in the Treatment of Irritability in Children and Adolescents with Autism Spectrum Disorder in Japan: A Randomized, Double-blind, Placebo-controlled Study. *Child psychiatry and human development* 2017; **48(5):** 796-806.

25. Kent JM, Kushner S, Ning X, Karcher K, Ness S, Aman M *et al.* Risperidone dosing in children and adolescents with autistic disorder: a double-blind, placebo-controlled study. *J Autism Dev Disord* 2013; **43**(8)**:** 1773-1783.

26. Kowatch RA, Scheffer RE, Monroe E, Delgado S, Altaye M, Lagory D. Placebo-controlled trial of valproic Acid versus risperidone in children 3-7 years of age with bipolar I disorder. *J Child Adolesc Psychopharmacol* 2015; **25**(4)**:** 306-313.

27. Lamberti M, Siracusano R, Italiano D, Alosi N, Cucinotta F, Di Rosa G *et al.* Head-to-Head Comparison of Aripiprazole and Risperidone in the Treatment of ADHD Symptoms in Children with Autistic Spectrum Disorder and ADHD: A Pilot, Open-Label, Randomized Controlled Study. *Paediatr Drugs* 2016; **18**(4)**:** 319-329.

28. Loebel A, Brams M, Goldman RS, Silva R, Hernandez D, Deng L *et al.* Lurasidone for the Treatment of Irritability Associated with Autistic Disorder. *J Autism Dev Disord* 2016; **46**(4)**:** 1153-1163.

29. Kryzhanovskaya L, Schulz SC, McDougle C, Frazier J, Dittmann R, Robertson-Plouch C *et al.* Olanzapine versus placebo in adolescents with schizophrenia: A 6-week, randomized, double-blind, placebo-controlled trial. *Journal of the American Academy of Child and Adolescent Psychiatry* 2009; **48**(1)**:** 60-70.

30. Kumra S, Frazier JA, Jacobsen LK, McKenna K, Gordon CT, Lenane MC *et al.* Childhood-onset schizophrenia: A double-blind clozapine-haloperidol comparison. *Archives of General Psychiatry* 1996; **53**(12)**:** 1090-1097.

31. Kumra S, Kranzler H, Gerbino-Rosen G, Kester HM, DeThomas C, Kafantaris V *et al.* Clozapine and "high-dose" olanzapine in refractory early-onset schizophrenia: A 12-week randomized and double-blind comparison. *Biological Psychiatry* 2008; **63**(5)**:** 524-529.

32. Marcus RN, Owen R, Kamen L, Manos G, McQuade RD, Carson WH *et al.* A placebo-controlled, fixed-dose study of aripiprazole in children and adolescents with irritability associated with autistic disorder. *J Am Acad Child Adolesc Psychiatry* 2009; **48**(11)**:** 1110-1119.

33. McCracken JT, McGough J, Shah B, Cronin P, Hong D, Aman MG *et al.* Risperidone in children with autism and serious behavioral problems. *N Engl J Med* 2002; **347**(5)**:** 314-321.

34. Miral S, Gencer O, Inal-Emiroglu FN, Baykara B, Baykara A, Dirik E. Risperidone versus haloperidol in children and adolescents with AD : a randomized, controlled, double-blind trial. *Eur Child Adolesc Psychiatry* 2008; **17**(1)**:** 1-8.

35. Nicol GE, Yingling MD, Flavin KS, Schweiger JA, Patterson BW, Schechtman KB *et al.* Metabolic Effects of Antipsychotics on Adiposity and Insulin Sensitivity in Youths: A Randomized Clinical Trial. *JAMA Psychiatry* 2018; **75**(8)**:** 788-796.

36. Pagsberg AK, Jeppesen P, Klauber DG, Jensen KG, Ruda D, Stentebjerg-Olesen M *et al.* Quetiapine extended release versus aripiprazole in children and adolescents with first-episode psychosis: the multicentre, double-blind, randomised tolerability and efficacy of antipsychotics (TEA) trial. *The Lancet Psychiatry* 2017; **4**(8)**:** 605-618.

37. Pathak S, Findling RL, Earley WR, Acevedo LD, Stankowski J, Delbello MP. Efficacy and safety of quetiapine in children and adolescents with mania associated with bipolar I disorder: a 3-week, double-blind, placebo-controlled trial. *J Clin Psychiatry* 2013; **74**(1)**:** e100-109.

38. Safavi P, Hasanpour-Dehkordi A, AmirAhmadi M. Comparison of risperidone and aripiprazole in the treatment of preschool children with disruptive behavior disorder and attention deficit-hyperactivity disorder: A randomized clinical trial. *J Adv Pharm Technol Res* 2016; **7**(2)**:** 43-47.

39. Saito T, Sugimoto S, Sakaguchi R, Nakamura H, Ishigooka J. Efficacy and safety of blonanserin oral tablet in adolescents with schizophrenia: A 6-week, randomized placebo-controlled study. *Journal of Child and Adolescent Psychopharmacology* 2022; **32**(1)**:** 12-23.

40. Sallee FR, Kurlan R, Goetz CG, Singer H, Scahill L, Law G *et al.* Ziprasidone Treatment of Children and Adolescents With Tourette's Syndrome: A Pilot Study. *Journal of the American Academy of Child & Adolescent Psychiatry* 2000; **39**(3)**:** 292-299.

41. Sallee F, Kohegyi E, Zhao J, McQuade R, Cox K, Sanchez R *et al.* Randomized, Double-Blind, Placebo-Controlled Trial Demonstrates the Efficacy and Safety of Oral Aripiprazole for the Treatment of Tourette's Disorder in Children and Adolescents. *J Child Adolesc Psychopharmacol* 2017; **27**(9)**:** 771-781.

42. Savitz AJ, Lane R, Nuamah I, Gopal S, Hough D. Efficacy and safety of paliperidone extended release in adolescents with schizophrenia: A randomized, double-blind study. *Journal of the American Academy of Child and Adolescent Psychiatry* 2015; **54**(2)**:** 126-137.e121.

43. Shaw P, Sporn A, Gogtay N, Overman GP, Greenstein D, Gochman P *et al.* Childhood-onset schizophrenia: A double-blind, randomized clozapine-olanzapine comparison. *Archives of General Psychiatry* 2006; **63**(7)**:** 721-730.

44. Shea S, Turgay A, Carroll A, Schulz M, Orlik H, Smith I *et al.* Risperidone in the treatment of disruptive behavioral symptoms in children with autistic and other pervasive developmental disorders. *Pediatrics* 2004; **114**(5)**:** e634-641.

45. Sikich L, Frazier JA, McClellan J, Findling RL, Vitiello B, Ritz L *et al.* Double-blind comparison of first- and second-generation antipsychotics in early-onset schizophrenia and schizoaffective disorder: Findings from the treatment of early-onset schizophrenia spectrum disorders (TEOSS) study. *American Journal of Psychiatry* 2008; **165**(11)**:** 1420-1431.

46. Singh J, Robb A, Vijapurkar U, Nuamah I, Hough D. A randomized, double-blind study of paliperidone extended-release in treatment of acute schizophrenia in adolescents. *Biological Psychiatry* 2011; **70**(12)**:** 1179-1187.

47. Snyder R, Turgay A, Aman M, Binder C, Fisman S, Carroll A. Effects of Risperidone on Conduct and Disruptive Behavior Disorders in Children With Subaverage IQs. *Journal of the American Academy of Child & Adolescent Psychiatry* 2002; **41**(9)**:** 1026-1036.

48. Mauricio Tohen MD, Dr.P.H. ,, Ludmila Kryzhanovskaya MD, Ph.D. ,, Gabrielle Carlson MD, Melissa DelBello MD, M.S. ,, Janet Wozniak MD, Robert Kowatch MD *et al.* Olanzapine Versus Placebo in the Treatment of Adolescents With Bipolar Mania. *American Journal of Psychiatry* 2007; **164**(10)**:** 1547-1556.

49. Yoo HK, Joung YS, Lee JS, Song DH, Lee YS, Kim JW *et al.* A multicenter, randomized, double-blind, placebo-controlled study of aripiprazole in children and adolescents with Tourette's disorder. *J Clin Psychiatry* 2013; **74**(8)**:** e772-780.
